# Structure-Based Molecular Networking for the Discovery of Anti-HBV Compounds from *Saussurea lappa* (Decne.) C.B Clarke

**DOI:** 10.3390/molecules27062023

**Published:** 2022-03-21

**Authors:** Tao Wu, Xin-Jian Yan, Tian-Rong Yang, Yun-Fen Wang, Jing-Yi He, Yang Feng, Li-Hua Su, Hao Chen, Min Xu

**Affiliations:** Center for Pharmaceutical Sciences, Faculty of Life Science and Technology, Chenggong Campus, Kunming University of Science and Technology, Kunming 650500, China; 20172118076@stu.kust.edu.cn (T.W.); yanxinjian@stu.kust.edu.cn (X.-J.Y.); 20202118059@stu.kust.edu.cn (T.-R.Y.); wyf_333@163.com (Y.-F.W.); hjingyi0302@163.com (J.-Y.H.); fengy@kust.edu.cn (Y.F.); 20210047@kut.edu.cn (L.-H.S.)

**Keywords:** *Saussurea lappa*, sesquiterpenes, anti-HBV activity, structure-guided isolation

## Abstract

It is a crucial to find target compounds in natural product research. This study presents a concept of structure-guided isolation to find candidate active molecules from herbs. We establish a process of anti-viral sesquiterpene networking. An analysis of the networking suggested that new anti-HBV sesquiterpene may be attributable to eudesmane-, guaiane-, cadinane-, germacane- and bisabolane-type sesquiterpenes. In order to evaluate the efficiency of the structure-based molecular networking, ethanol extract of *Saussurea lappa* (Decne.) C.B Clarke was investigated, which led to the isolation of two guaiane-type (**1** and **14**), ten eudesmane-type (**2**–**5** and **8**–**13**), two chain (**6** and **7**) and one germacrane-type (**15**) sesquiterpenes, including seven new ones, lappaterpenes A–G (**1**–**7**), which are reported on herein. The absolute configurations of the new compounds were established by coupling constants, calculated ECD and ROESY correlations, as well as comparisons of optical rotation values with those of known compounds. The absolute configuration of compound **2** was further confirmed by X-ray diffraction. Compounds **1**–**15** were evaluated for their potency against hepatitis B virus. Compounds **4**, **6**, **7** and **9** showed effect on HBsAg with inhibition ratios of more than 40% at 30 μM concentrations. Compounds **14** and **15** inhibited HBsAg secretion with the values of IC_50_ 0.73 ± 0.18 and 1.43 ± 0.54 μM, respectively. Structure-based molecular networking inspired the discovery of target compounds.

## 1. Introduction

Natural product structures play a significant role in drug discovery and development [1,2]. Bioactive natural products offer opportunities to discover novel targets and mechanisms for treating human diseases [3]. However, there is a lack of effective methods to find target compounds. Previously, ethnopharmacological knowledge or screening of extract for bioactivity and bioassay-guided isolation have inspired the discovery of active natural products [4,5]. In recent years, bioactivity-based molecular networking has significantly increased the efficiency with which active natural products as potential drug leads have been discovered [6,7].

As part of an ongoing effort to find anti-viral sesquiterpenes in herbs in Yunnan province in China [8,9,10], herein we establish a new strategy, i.e., structure-based molecular networking, to investigate antiviral sesquiterpenes. Firstly, we collected antiviral sesquiterpenes from ZINC and CHEMBL. Secondly, based on the skeletons of the sesquiterpenes, we calculated the degrees of similarity of those sesquiterpenes by ChemmineR and ChemmineBO [11]. Lastly, we constructed a structure-based molecular network and divided the sesquiterpenes into communities by cluster_louvain from igraph [12], which led to the identification of key nodes (i.e., representative sesquiterpenes) in sesquiterpene communities. A cluster analysis of the sesquiterpene network suggested that new anti-HBV sesquiterpenes may be attributable to eudesmane- (degree = 27), guaiane- (degree = 21), cadinane- (degree = 20), germacane- (degree = 18) and bisabolane-type (degree = 16) sesquiterpenes (Table S1). Previous studies showed that bisabolane-type sesquiterpenes have good anti-HBV activity [13]. 

In order to evaluate the efficiency of the structure-based molecular networking, ethanol extract of *Saussurea lappa* (Decne.) C.B Clarke was investigated. *S. lappa* belongs to *Saussurea*, a large genus of the Asteraceae family including more than 400 species distributed worldwide [2], more than 40 of which have been used in traditional and alternative medicine [14]. Nowadays, the plant mainly is cultivated in Yunnan province in China. The roots of *S. lappa* are recorded in Chinese Pharmacopoeia (2020 Edition) [15]. It is also a common Tibetan medicine and has been used to treat stomach pain and blood disorders. In Mongolian medicine, the roots of *S. lappa* have been used to treat lung abscesses and phlegm [16]. Some eudesmane-, guaiane- and germacane-type sesquiterpenes have been isolated from *S. lappa*. These compounds showed potential effects on chronic superficial gastritis, ulcer, cancer, bacterial, fungal and viral diseases [16,17,18]. 

**Figure 1:**
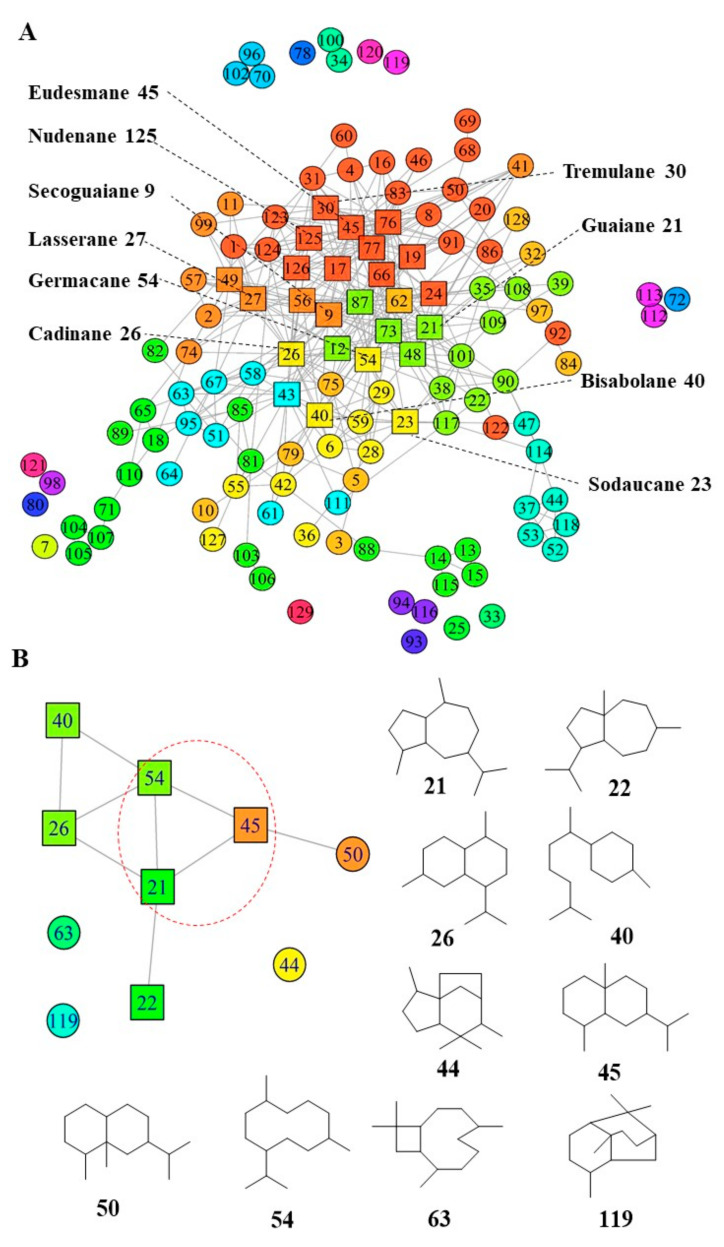

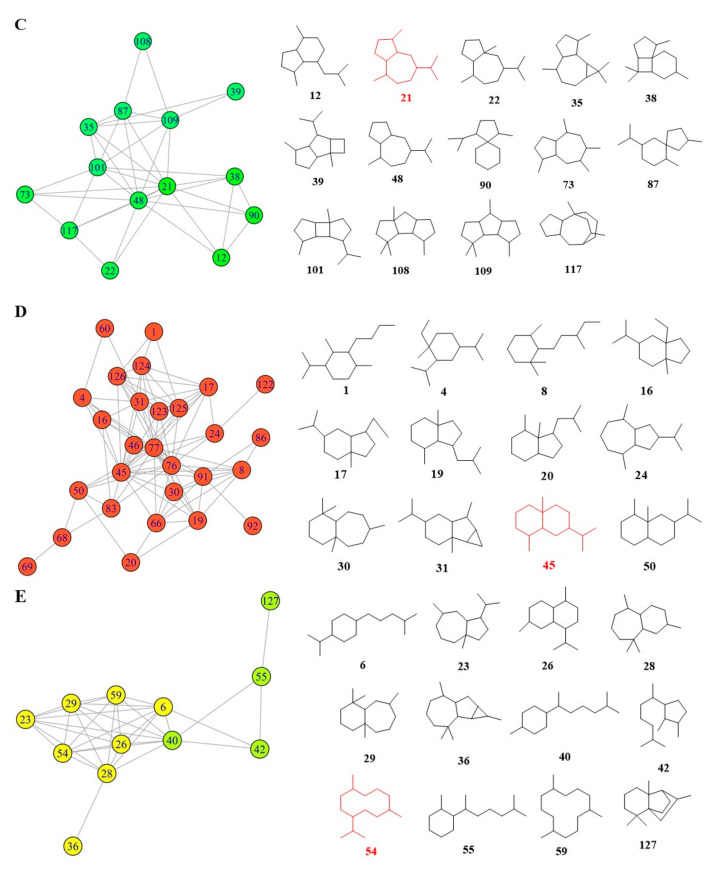
Network analysis identifying key sesquiterpenes. (**A**) Sesquiterpenes network graph; square nodes represent higher values of degree than other nodes (shown as circles). The sesquiterpenes were clustered by the cluster_louvain method, and their communities were colored by rainbow package (start: red, middle: green, end: green). (**B**) Community of sesquiterpenes with anti-HBV activity. Nodes 21, 45 and 54 are key sesquiterpenes. (**C**) The community with node 21 (guaiane). (**D**) The community with node 45 (eudesmane). (**E**) The community with node 54 (germacrane).

Herein we report seven new sesquiterpenes from the roots of *S. lappa*, namely, lappaterpenes A–G (**1**–**7**) (Figure 1), as well as eight known sesquiterpenes, 11-hydroxy-4*α*-methoxyselinane (**8**) [19], ilicic aldehyde (**9**) [20], 4*α*-hydroxy-4*β*-methyldihydrocostol (**10**) [21], arbusculin E methyl ester (**11**) [22], 5*α*-hydroxy-*β*-costol (**12**) [23], arbusculin A (**13**) [24,25], dehydrocostus lactone (**14**) [24], and costunolide (**15**) [26]. Through X-ray diffraction and calculated ECD, the absolute configurations of Compounds **1**−**5** were confirmed. The absolute configurations of other two new compounds (**6** and **7**) were established by comparing the optical rotation values with those of known compounds, as well as coupling constants and ROESY correlations. All compounds were tested for anti-HBV activities. Compounds **4**, **6**, **7** and **9** showed effect on HBsAg, with inhibition ratios of more than 40% at 30 μM concentrations. Compounds **14** and **15** inhibited HBsAg secretion at IC_50_ values of 0.73 ± 0.18 and 1.43 ± 0.54 μM, respectively. Compounds **14** and **15** also showed potential cytotoxic activity in HepG2, while Compounds **4**, **6**, **7**, and **9** showed no cytotoxic activity at 30 μM. Structure-based molecular networking inspired the discovery of active natural products.

## 2. Results

### 2.1. Development of Structure-Based Molecular Networking

ChemmineR and ChemmineOB were applied to calculate similarities among sesquiterpenes. When a threshold value of similarity is 90%, similar sesquiterpenes can be identified and clustered by cluster_louvain to categorize the compounds into different communities. Key nodes with the highest degree values, and other similar nodes, are displayed as square nodes (Figure 1A). The top ten square nodes are listed in Figure 1A and Table S1. In the molecular networking, anti-HBV sesquiterpenes (Table S2) were extracted from Figure 1A; in Figure 1B, nodes 21 (Guaiane, degree = 21), 45 (Eudesmane, degree = 27) and 54 (Germacrane, degree = 18) are key nodes. Node 21 represents guaiane, located in the green community in Figure 1A; its community and related sesquiterpenes are illustrated in Figure 1C. Node 45 represents eudesmane, located in the red community in Figure 1A; its community and related sesquiterpenes are illustrated in Figure 1D. Node 54 represents germacrene, located in the yellow community in Figure 1A; related sesquiterpenes are illustrated in Figure 1E. The sources of germacrene-, guaiane- and eudesmane-type sesquiterpenes were searched in our database, which showed that these three kinds of sesquiterpene skeletons were simultaneously enriched in *Saussurea* spp. (Table S2). 

### 2.2. Evaluation of Workflow Procedure

**Figure 2:**
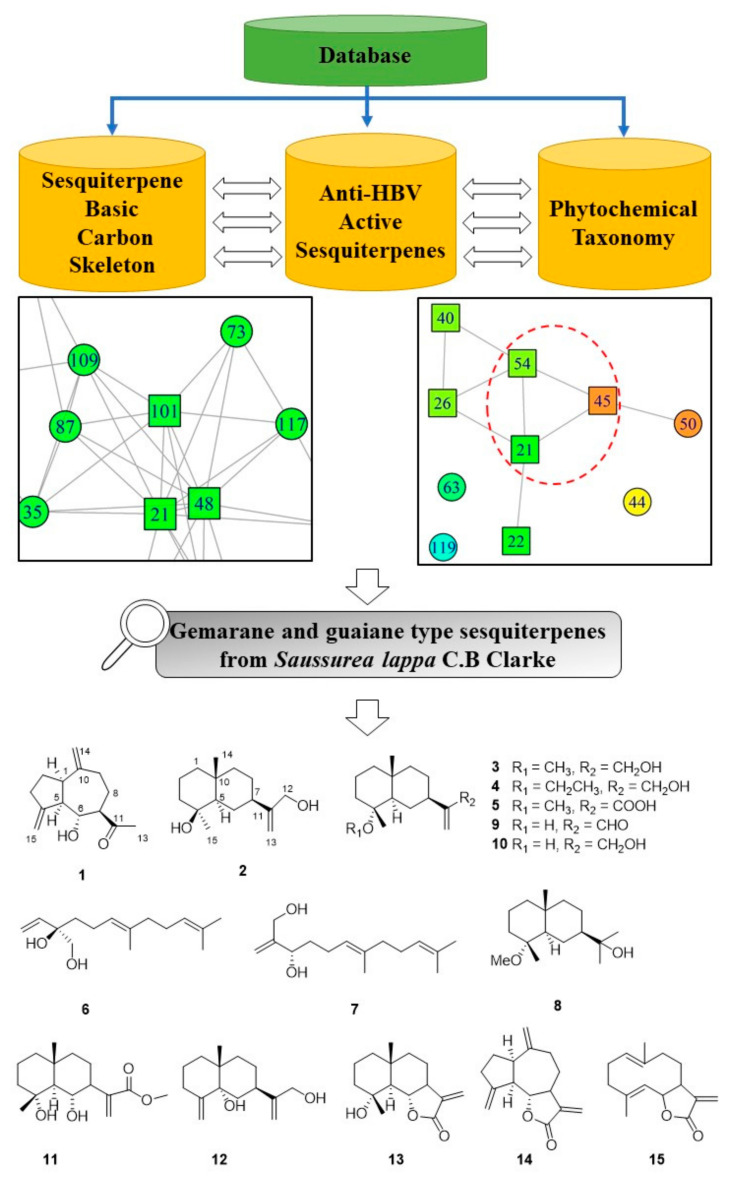
New Compounds **1**–**15** from the roots of *S. lappa*.

To evaluate the efficiency of the structure-based molecular networking method, ethanol extract of *S. lappa* was investigated, which led to the isolation of seven new sesquiterpenes, as well as eight known sesquiterpenes (shown in Figure 2). The known compounds were identified as 4*α*-methoxyselinane (**8**) [19], ilicic aldehyde (**9**) [20], 4*α*-hydroxy-4*β*-methyldihydrocostol (**10**) [21], arbusculin E methyl ester (**11**) [22], 5*α*-hydroxy-*β*-costol (**12**) [23], arbusculin A (**13**) [25], dehydrocostus lactone (**14**) [24], and costunolide (**15**) [26], respectively. By X-ray diffraction and calculated ECD, the absolute configurations of Compounds **1**–**5** were confirmed, while those of Compounds **6** and **7** were established by comparing optical rotation values with those of known compounds, as well as coupling constants and ROESY correlations.

**Figure 3:**
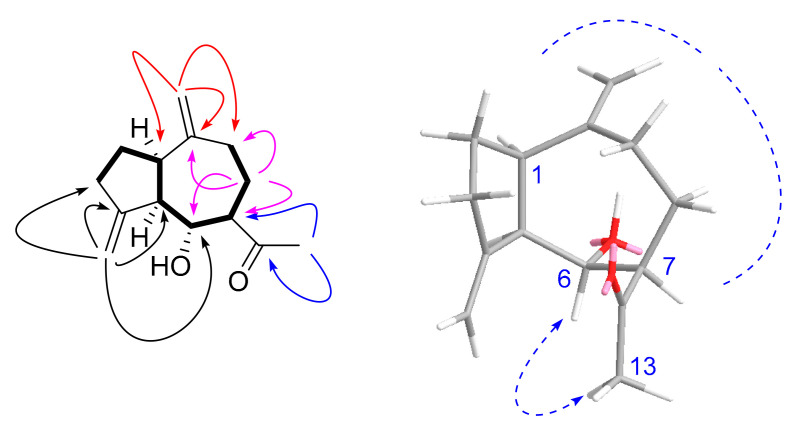
Key HMBC (→), ^1^H-^1^H COSY (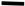) and ROESY (↔) correlations for Compound **1**.

**Figure 4:**
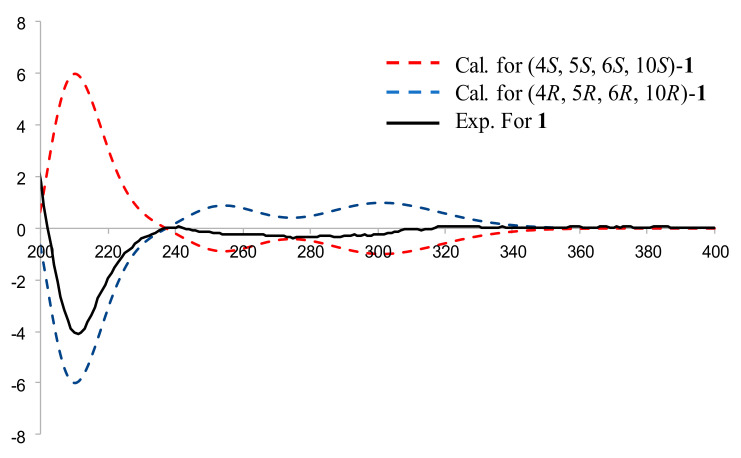
Comparison of the calculated ECD spectrum for (4*S*, 5*S*, 6*S*, 10*S*)-**1** at B3LYP/6-31G(d) level with the experimental spectrum of **1** in MeOH. σ = 0.3 eV, shift = −10 nm.

Compound **1**, a pale-yellow oily substance, ${\left[\alpha \right]}_{D}^{23.1}$ −30.1 ± 0.12 (c 0.3, MeOH), had the molecular formula of C_14_H_20_O_2_ (∆mmu 0.13), as deduced from the (+) HRESIMS (Calcd. for C_14_H_20_O_2_Na, *m*/*z* 243.2699), ^13^C NMR and HSQC data. The spectrum for ^1^H NMR (MeOH-*d*_4_) (shown in Table 1) revealed one singlet methyl (δ_H_ 2.15) and two terminal olefinic protons (δ_H_ 5.01, 5.04, 4.77 and 4.86). The ^13^C NMR (MeOH-*d*_4_) and HSQC spectra (showed in Table 1) suggested 14 carbons including one methyl (δ_C_ 27.8), six methylenes including two alkenyls (δ_C_ 109.5 and 110.0), four methines (δ_C_ 47.1, 54.7, 61.4, and 69.5) and three quaternary carbons including a ketone carbonyl (δ_C_ 212.9) and two alkenyls (δ_C_ 151.7 and 151.9). The aforementioned NMR data of **1** were similar to those of guaiane-type sesquiterpene watsonol A [27]. However, they presented additional signals of methyl ketone. Furthermore, HMBC correlations (Figure 3) of the methyl (δ_H_ 2.18, Me-13) to the additional carbonyl (δ_C_ 212.9, C-11) and methine (δ_C_ 61.4, C-7) confirmed the additional methyl ketone linked to C-7 of **1**, as well as HMBC correlations of alkenyl (δ_H_ 5.04, 5.07, H-14) to C-1/C-9/C-10, alkenyl (δ_H_ 4.88, H-15) to C-3/C-4/C-5 (shown in Figure 3). Thus, the planar structure of **1** was assigned as shown in Figure 2. The relative configuration was further determined by ROESY correlations. The ROESY correlations of H-7/H-1 (shown in Figure 3) indicated that H-1, H-5 and H-7 were α-oriented, while the ROESY correlations of H-6/Me-13 (shown in Figure 3) indicated that HO-6 were α-oriented. In order to further clarify the absolute configuration of **1**, the low energy conformers of the compound were optimized by applying the DFT method at the B3LYP/6-31G(d) level (in MeOH). The results showed that the calculated ECD spectrum (shown in Figure 4) of (4*R*, 5*R*, 6*R*, 10*R*)-**1** agreed well with experimental data, which indicated that the absolute configuration of **1** was 4*R*, 5*R*, 6*R*, 10*R*. As a result, the structure of **1** was assigned and the compound was named lappaterpene A.

**Figure 5:**
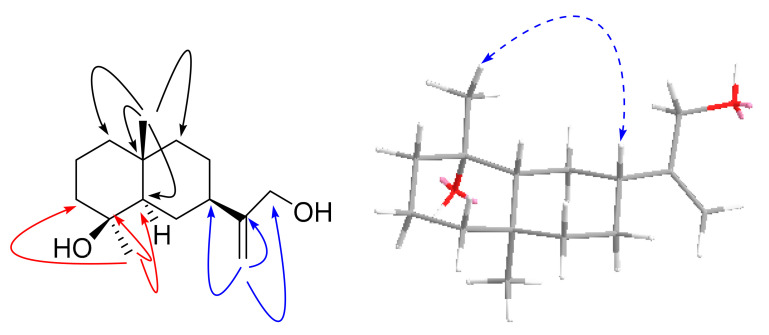
Key HMBC (→) and ROESY (↔) correlations for Compound **2**.

**Figure 6:**
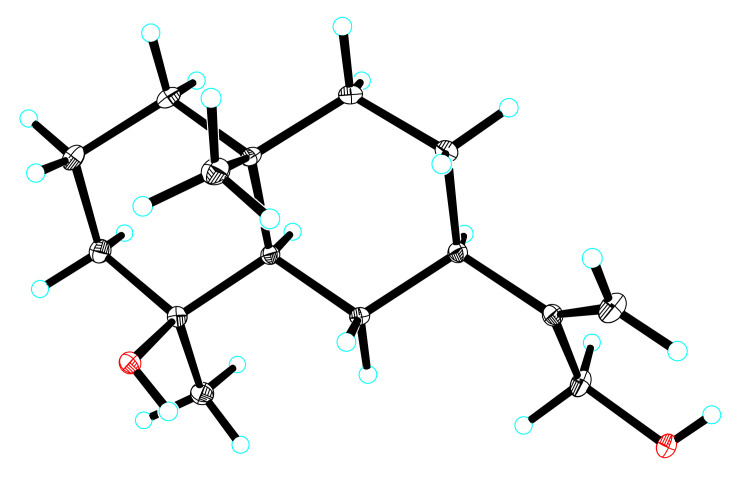
Single crystal X-ray structure of Compound **2**.

Compound **2**, a colorless needle crystal, ${\left[\alpha \right]}_{D}^{23.2}$ 12.2 ± 0.31 (c 0.15, MeOH), had the molecular formula of C_15_H_26_O_2_ (∆mmu 0.20), as deduced from the HRESIMS (Calcd. for C_15_H_26_O_2_Na, *m*/*z* 261.1826), ^13^C NMR and HSQC data. The ^1^H NMR (MeOH-*d*_4_) data for **2** (shown in Table 1) showed two singlet methyls (δ_H_ 1.13, 1.07) and a terminal olefinic proton (δ_H_ 5.02, 4.91). The ^13^C NMR (MeOH-*d*_4_) data for **2** (shown in Table 1) suggested 15 carbons including two methyls (δ_C_ 29.1 and 17.9), eight methylenes including an oxymethylene (δ_C_ 63.8) and a terminal olefin (δ_C_ 106.6), two methines (δ_C_ 52.1 and 42.3) and three quaternary carbons including one oxy-quaternary carbon (δ_C_ 71.0) and one alkenyl (δ_C_ 154.2). The aforementioned NMR data of **2** were similar to those of 4*α*-hydroxy-4*β*-methyldihydrocostol (**10**) [21], an eudesmane sesquiterpene; however, they differed in that C-2, C-3, C-4 and C-5 of Compound **2** were shifted to high field by 1.8, 1.8, 0.5 and 2.3 ppm, respectively, and C-1 and C-15 were shifted to low field by 1.0, and 7.9 ppm, respectively. The above data suggest that Compound **2** may be an epimer of Compound **10**. Furthermore, HMBC correlations of methyl (δ_H_ 1.07, Me-15) to C-3/C-4/C-5, methyl (δ_H_ 1.13, Me-14) to C-1/C-5/C-9/C-10 and methylene (δ_H_ 4.06, CH_2_-13) to C-7/C-11/C-12 (shown in Figure 5), as well as other key HMBC related signals, confirmed the planar structure of Compound **2** (shown in Figure 5). The relative configuration of **2** was established using the ROESY spectrum. The ROESY correlation of H-5 with Me-15/H-7 indicated that Me-15 and H-7 were α-oriented. (shown in Figure 5). Furthermore, a crystal X-ray diffraction experiment with Cu Kα radiation further allowed the unambiguous assignment of the absolute configuration of **2** (shown in Figure 6) (deposition No. CCDC: 2061652) as 4*S*, 5*R*, 7*R*, 10*R* [the Flack parameter is 0.03 (5) and the Hooft parameter is 0.10 (4) for 2102 Bijvoet pairs] [28,29]. Compound **2** was only reported as a bioconversion product by PCT patent plant enzymes [30]. However, its absolute configuration was not determined and detailed NMR data were unavailable. Herein we report compound **2** as a new natural product which we named lappaterpene B.

**Figure 7:**
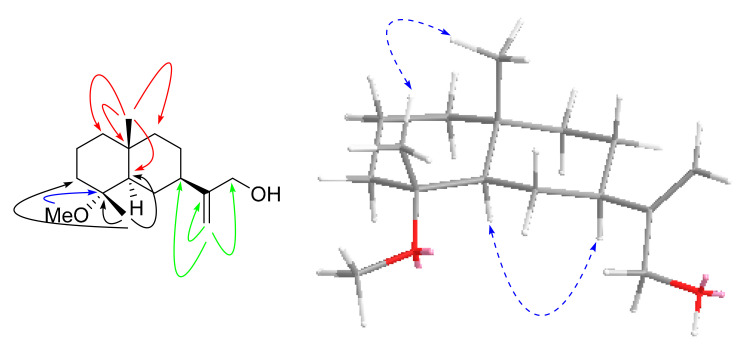
Key HMBC (→) and ROESY (↔) correlations for Compound **3**.

**Figure 8:**
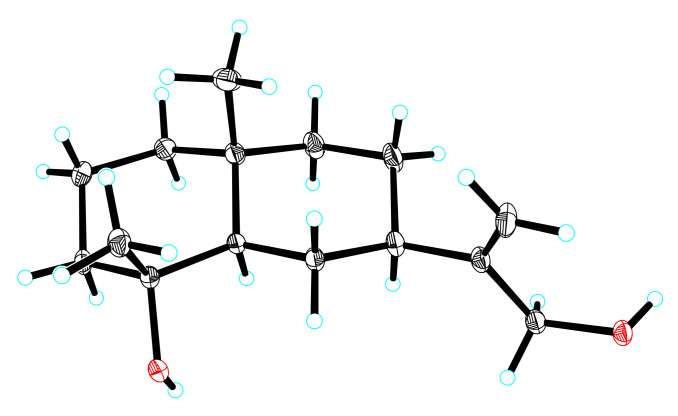
Single crystal X-ray structure of Compound **10**.

**Figure 9:**
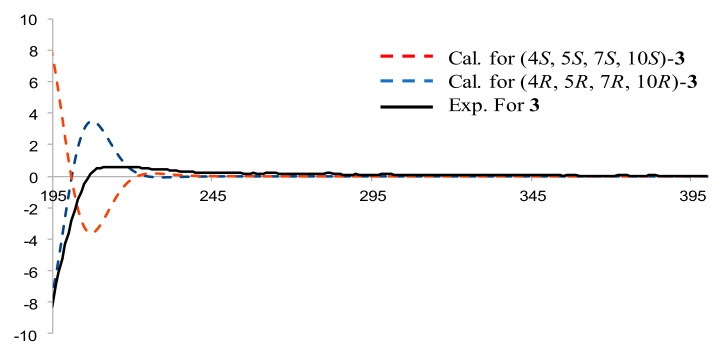
Comparison of the calculated ECD spectrum for (4*R*, 5*R*, 7*R*, 10*R*) −**3** at B3LYP/6-31G(d) level with the experimental spectrum of **3** in MeOH. σ = 0.3 eV, shift = +22 nm.

Compound **3**, a pale-yellow oily substance, ${\left[\alpha \right]}_{D}^{22.2}$ −58.4 ± 0.10 (c 0.15, MeOH), had the molecular formula of C_16_H_28_O_2_ (∆mmu 0.01), as deduced from the HRESIMS (Calcd. for C_16_H_28_O_2_Na, *m*/*z* 275.2012), ^13^C NMR and HSQC data. The ^1^H and ^13^C NMR (MeOH-*d*_4_) data for **3** (shown in Table 2) were similar to those of 4*α*-hydroxy-4*β*-methyldihydrocostol (**10**) [21], but the difference is the molecular weight, which has an extra methoxy group. Furthermore, HMBC correlations of additional methoxyl (δ_H_ 3.20, OCH_3_) to C-4 (δ_C_ 76.4) further confirmed the substituted position of the methoxyl, as well as the HBMC correlations of methyl (δ_H_ 0.97, H_3_-15) to C-3/C-4/C-5, methyl (δ_H_ 1.08, H_3_-14) to C-1/C-5/C-9/C-10, and methylene (δ_H_ 5.01, 4.88, H_2_-13) to C-7/C-11/C-12 (shown in Figure 7). Thus, the planar structure of **3** was assigned as shown in Figure 2. The relative configuration was further determined by ROESY correlations of Me-1/Me-15 and H-7/H-5 (shown in Figure 7). In order to evaluate Compound **3**, we obtained 4*α*-hydroxy-4*β*-methyldihydrocostol (**10**) crystals, a major compound of *Saussurea* spp. A crystal X-ray diffraction experiment with Cu Kα radiation further confirmed the absolute configuration of **10** (deposition No. CCDC: 2026706) (shown in Figure 8) as 4*R*, 5*R*, 7*R*, 10*R* [the Flack parameter is −0.01 (7); the Hooft parameter was 0.10 (4) for 2102 Bijvoet pairs] [28,29]. On this basis, the absolute configuration of **3** was shown to be 4*R*, 5*R*, 7*R*, 10*R*. In order to further clarify the absolute configuration of **3**, the low energy conformers of the compound were optimized by applying the DFT method at the B3LYP/6-31G(d,p) level (in MeOH). The results showed that the calculated ECD spectrum (shown in Figure 9) of (4*R*, 5*R*, 7*R*, 10*R*)-**3** agreed well with the experimental data. The aforementioned data allowed the unambiguous assignment of **3** as 4*R*, 5*R*, 7*R*, 10*R*. As a result, the structure of **3** was assigned and the compound was named lappaterpene C.

**Figure 10:**
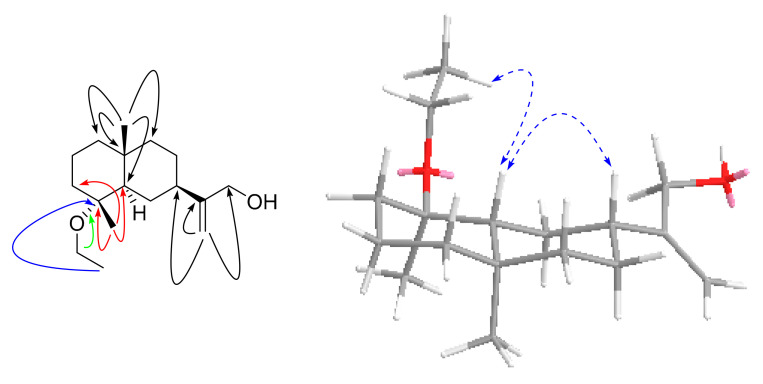
Key HMBC (→) and ROESY (↔) correlations for Compound **4**.

**Figure 11:**
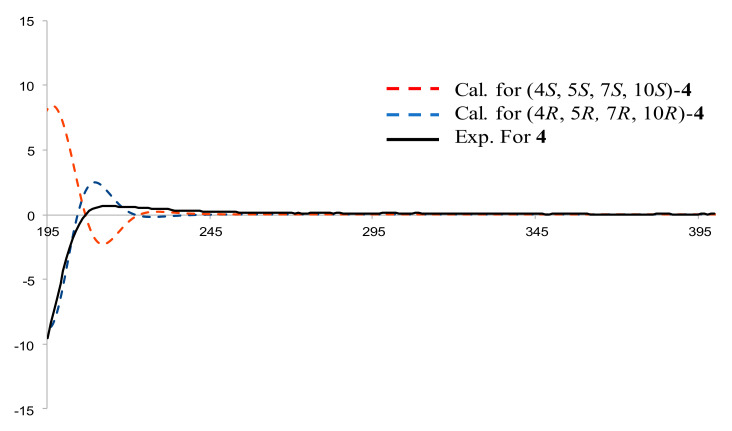
Comparison of the calculated ECD spectrum for (4*R*, 5*R*, 7*R*, 10*R*) −**4** at B3LYP/6-31G(d) level with the experimental spectrum of **4** in MeOH. σ = 0.3 eV, shift = +22 nm.

Compound **4**, a pale-yellow oily substance, ${\left[\alpha \right]}_{D}^{22.6}$ −24.7 ± 0.07 (c 0.25, MeOH), had the molecular formula of C_17_H_30_O_2_ (∆mmu 0.20), as deduced from the HRESIMS (Calcd. for C_17_H_30_O_2_Na, *m*/*z* 289.3594), ^13^C NMR and HSQC data. The ^1^H and ^13^C NMR (MeOH-*d*_4_) data for **4** (shown in Table 2) were similar to those of Compound **3**, but differed in molecular weight and through the presence of an additional ethoxyl group. HMBC correlations of oxy-methylene (δ_H_ 3.38, 3.43, CH_2_) to C-4 (δ_C_ 76.3) and (δ_H_ 1.11, Me) to oxy-methylene (δ_C_ 54.6, CH_2_) further confirmed the substituted position of the ethyoxyl, as well as the other key HBMC correlations (shown in Figure 10). Thus, the planar structure of **4** was assigned as shown in Figure 1. The relative configuration was further determined by ROESY correlations of H-5/H-7 and Me-15/Me-14 (shown in Figure 10). In order to further clarify the absolute configuration of **4**, the low energy conformers of the compound were optimized by applying the DFT method at the B3LYP/6-31G(d,p) level (in MeOH). The results showed that the calculated ECD spectrum (shown in Figure 11) of (4*R*, 5*R*, 7*R*, 10*R*)-**4** agreed well with the experimental one, indicating that the absolute configuration of **4** was 4*R*, 5*R*, 7*R*, 10*R*. As a result, the structure of **4** was assigned and the compound was named lappaterpene D. 

**Figure 12:**
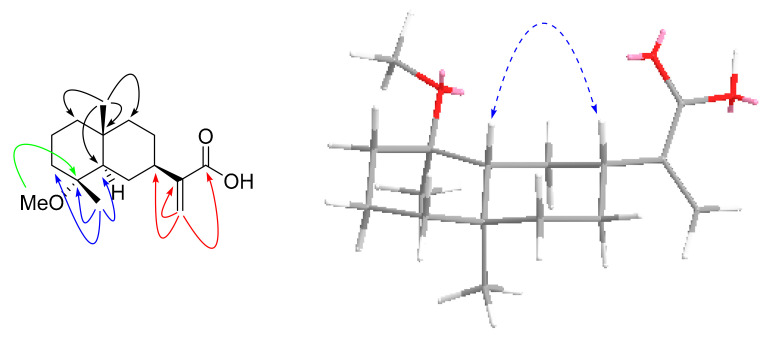
Key HMBC (→) and ROESY (↔) correlations for Compound **5**.

**Figure 13:**
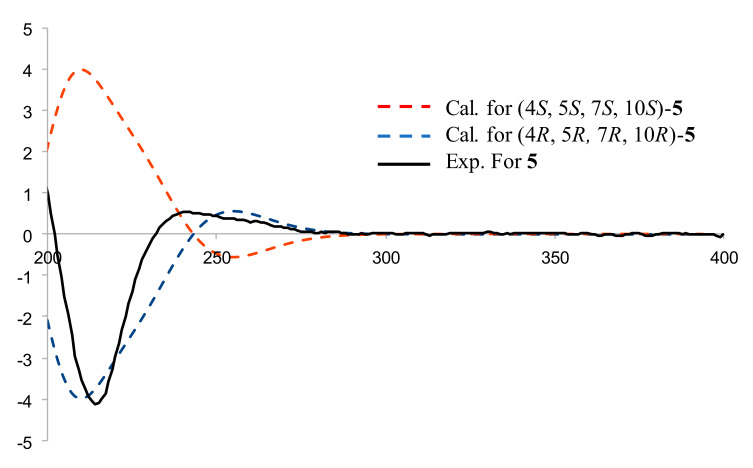
Comparison of the calculated ECD spectrum for (4*R*, 5*R*, 7*R*, 10*R*)-**5** at B3LYP/6-31G(d) level with the experimental spectrum of **5** in MeOH. σ = 0.3 eV, shift = −20 nm.

Compound **5**, a pale-yellow oily substance, ${\left[\alpha \right]}_{D}^{22.8}$ −29.4 ± 0.11 (c 0.15, MeOH), had the molecular formula of C_16_H_26_O_3_ (∆mmu 0.03), as deduced from the HRESIMS (Calcd. for C_16_H_26_O_3_Na, *m*/*z* 289.1780), ^13^C NMR and HSQC data. The ^1^H NMR and ^13^C NMR (MeOH-*d*_4_) data for **5** (shown in Table 2) were similar to those of Compound **3**, but differed in molecular weight and through the presence of additional carbonyl group. HMBC correlations of methylene (δ_H_ 5.52, 6.07, C-13) to additional carbonyl (δ_C_ 169.8, C-12) and methine (δ_C_ 40.3, C-7) confirmed the additional carbonyl linked to C-11 of **5** (shown in Figure 12). The ROESY correlations of H-5 with H-7 (shown in Figure 12) revealed the relative configuration of **5**. In order to further clarify the absolute configuration of **5**, the low energy conformers of **5** were optimized by applying the DFT method at the B3LYP/6-31G(d,p) level (in MeOH). The results showed that the calculated ECD spectrum (shown in Figure 13) of (4*R*, 5*R*, 7*R*, 10*R*)-**5** agreed well with the experimental one, indicating the absolute configuration of **5** to be 4*R*, 5*R*, 7*R*, 10*R*. As a result, the structure of **5** was assigned and the compound was named lappaterpene E. 

**Figure 14:**
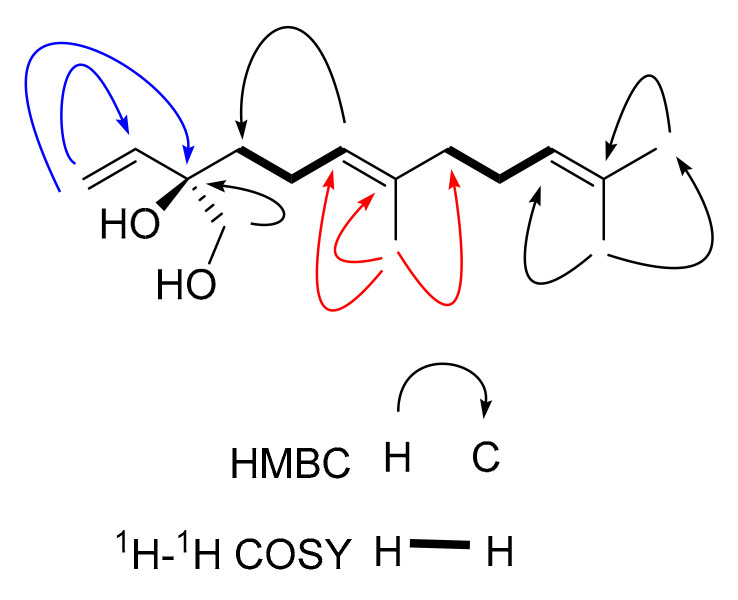
Key HMBC (→) and ^1^H-^1^H COSY (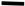) correlations for Compound **6**.

Compound **6**, a pale-yellow oily substance, ${\left[\alpha \right]}_{D}^{22.5}$ +0.32 ± 0.19 (c 0.15, MeOH), had the molecular formula of C_15_H_26_O_2_ (∆mmu 0.21), as deduced from the HRESIMS (Calcd. for C_15_H_26_O_2_Na, *m*/*z* 261.2774), ^13^C NMR and HSQC data. The ^1^H NMR (MeOH-*d*_4_) data for **6** (shown in Table 3) showed three methyls (δ_H_ 1.59, 1.59, 1.66) and five olefinic protons (δ_H_ 5.16, 5.29, 5.88, 5.12, 5.08). The ^13^C NMR (MeOH-*d*_4_) data for **6** (shown in Table 3) suggested 15 carbons including three methyls (δ_C_ 14.6, 16.4, 24.5), five methylenes (δ_C_ 36.5, 21.5, 39.5, 26.4, and 68.2), one quaternary carbon (δ_C_ 75.4) and three sets of alkenyls (δ_C_ 113.0, 141.2, 124.4, 134.5, 124.0, and 130.7). The aforementioned NMR data combined with mass suggested that Compound **6** was a chain-like sesquiterpene derivative. Furthermore, ^1^H-^1^H COSY correlations of H-4/H-5/H-6 and H-8/H-9/H-10 connected two molecular fragments (shown in Figure 14), as well as HMBC correlations of methyl (δ_H_ 1.59, H-13) to C-6/C-7/C-8, methyl (δ_H_ 1.59, H-14) to C-10/C-15, methylene (δ_H_ 3.45, C-12) to C-3, the methyl (δ_H_ 1.66, C-15) to C-11, methylene (δ_H_ 5.16, 5.29, C-1) to C-2/C-3 and methane (δ_H_ 5.12, H-6) to C-4 (shown in Figure 14). The ROESY correlations of H-6 with CH_2_-8 indicated that the double bond of in the molecule was a *cis* bond. Furthermore, since the crystal and suitable CD spectrum could not be obtained, we compared the optical rotation value of Compound **6** with that of (2*R*,6*E*)-5,9-undecadiene-1,2-diol, 2,6,10-trimethyl (${\left[\alpha \right]}_{D}^{23.4}$ +6) [31]. The result suggested that the absolute configuration of the C-3 was *R*. As a result, the structure of **6** was assigned and the compound was named lappaterpene F. 

**Figure 15:**
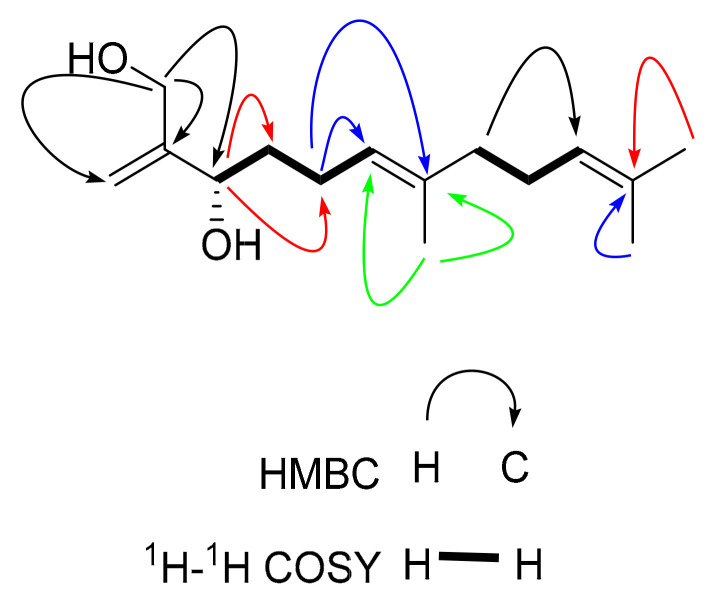
Key HMBC (→) and ^1^H-^1^H COSY (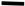) correlations for Compound **7**.

Compound **7**, a pale-yellow oily substance, ${\left[\alpha \right]}_{D}^{23.4}$ +3.18 ± 0.09 (c 0.25, MeOH), had a molecular formula of C_15_H_26_O_2_ (∆mmu 0.04), as deduced from the HRESIMS (Calcd. for C_15_H_26_O_2_Na, *m*/*z* 261.3211), ^13^C NMR and HSQC data. The ^1^H NMR (MeOH-*d*_4_) data for **7** (shown in Table 3) showed three methyls (δ_H_ 1.62, 1.61, 1.68) and three alkenyls including a terminal olefin (δ_H_ 5.09, 4.91). The ^13^C NMR (MeOH-*d*_4_) data for **7** (shown in Table 3) suggested 15 carbons including three methyls (δ_C_ 14.7, 16.4, 24.5), five methylenes including one oxymethylene (δ_C_ 65.1), one oxymethine (δ_C_ 75.2) and three sets of alkenyls (δ_C_ 109.7, 149.0, 124.0, 134.9, 123.9, 130.7). The aforementioned 1D NMR data combined with mass suggested that Compound **7** was a chain-type sesquiterpene derivative. Furthermore, ^1^H-^1^H COSY correlations of H-3/H-4/H-5/H-6 and H-8/H-9/H-10 to connect two molecular fragments (shown in Figure 15). In addition, HMBC correlations were determined of methylene (δ_H_ 5.09 and 4.91, H-12) to C-1/C-2/C-3, methine (δ_H_ 4.10, H-3) to C-4/C-5, methyl (δ_H_ 1.62, H-13) and methylene (δ_H_ 2.08 and 1.98, H-5) to C-6/C-7, methylene (δ_H_ 2.00, H-8) to C-10, methyl (δ_H_ 1.61, H-14) and methyl (δ_H_ 1.68, H-15) to C-11 (shown in Figure 15). Thus, the planar structure of **7** was assigned as shown in Figure 2. However, neither the crystal form not a suitable CD spectrum of **7** could be obtained. It is of note that Compound **7** shared same chiral centers with those of lysine [27]. The optical rotation value of Compound **7** with the value of +3.18 was similar to that of L-lysine, with the value of +10.2, but not to that of D-lysine, with the value of −12.8 [27]. As a result, the absolute configuration of the hydroxyl at the C-3 was speculated to be *S*. The structure of **7** was assigned and the compound was named lappaterpene G.

### 2.3. Anti-HBV Activities and SARs of Sesquiterpene Derivatives

*S. lappa* is a famous medicinal plant growing in the Himalayan region. It is now mainly is cultured in Yunnan and Sichuan provinces in China. The roots of *S. lappa* have been used to treat viral diseases in Ayurveda, Unani and Siddha as well as in traditional Chinese medicine. The plants have also been used in Tibet and other minority regions in China. Herein we reported the isolation and determination of seven new sesquiterpenes (**1**–**7**), together with eight known ones (**8**–**15**). All compounds were tested for their anti-HBV and cytotoxic activities. An anti-HBV assay suggested that known compounds dehydrocostus lactone (**14**) and costunolide (**15**) showed potent effects on HBsAg and HBeAg (displayed in Table 3), which agreed with the preliminary screening results. Active compounds (**14** and **15**) featured a *α*,*β*-unsaturated-lactone, and both showed inhibition toward both HBsAg and HBeAg. However, it was of note that the eudesmane sesquiterpene (**13**) bearing with *α*,*β*-unsaturated-lactone showed no activity against HBV at 30 µM, while new eudesmane sesquiterpene**s 4** and **9**, in which *α*,*β*-unsaturated-lactone was broken, showed some effect on HBsAg, with an inhibition ratio of more than 40% at 30 μM. Although further research will be required to evaluate the mechanism of HBV inhibition and the structure–activity relationships (SARs) of these compounds, the results suggest that germacrane and guaiane sesquiterpenes may be the anti-HBV active chemical constituents of *S. lappa*. This research is the first to report that eudesmane sesquiterpenes without *α*,*β*-unsaturated-lactone show moderate anti-HBV activity. 

## 3. Discussion

The loss of the bioactive compounds during isolation is a common problem in natural product research. Herein we present the concept of structure-guided isolation to find candidate active molecules directly from herbs. We established a library of antiviral sesquiterpenes which included structures, skeleton type, bioactivities and network of sesquiterpenes relationships to illustrate key node (i.e., representative sesquiterpenes). By employing similarity calculations, we constructed a sesquiterpene molecular network and categorized molecular clusters. Then, we used the bioactivity characteristics to filter the molecular clusters and predict the bioactivity of the sesquiterpenes in the clusters. We applied this workflow to discover antiviral compounds from an extract of *S. lappa*. It can be expected that this approach, i.e., structure-based molecular networking, will lead to the discovery of active natural products. It can be based on biologically active molecular networks for the analysis of bioassay-guided separation

## 4. Materials and Methods

### 4.1. General Experimental Techniques

Optical rotations were measured with a Jasco P-1020 digital polarimeter. IR spectra were measured on a Thermo NICOLET iS10 with KBr pellets. UV spectra were recorded on a Shimadzu UV-2700 spectrophotometer. CD spectra were measured on an Applied Photophysics Chirascan instrument. X-ray diffraction was measured on a Bruker D8 Quest instrument. ESIMS and HRESI-MS were run on an Agilent 1290 UPLC spectrometer and Agilent 6500 series Q-TOF spectrometer, respectively. NMR spectra were measured in CD_3_OD solution and recorded on a Bruker Avance III HD-600 or AV 800 spectrometer at 25 °C, using TMS as an internal standard. Chemical shifts were reported in units of δ (ppm), and coupling constants (*J*) were expressed in Hz. Column chromatography (CC) was carried out over silica gel (200–300 or 500–800 mesh, Qingdao Marine Chemical Factory), Sephadex LH-20 (25–100 μm, Pharmacia Fine Chemical Co., Ltd., Tokyo, Japan), MCI-gel CHP-20P (75–150 µm, Mitsubishi Chemical Industry, Ltd., Guangzhou, China), Rp-18 (40–63 μm, Merck, Shanghai, China). Precoated silica gel plates (Qingdao Haiyang Chemical Co., Qingdao, China) were used for TLC. Detection was done under UV light (254 nm and 365 nm) and by spraying the plates with 10% sulfuric acid followed by heating. A Waters 1525/2998 liquid chromatography machine (Waters Technologies, Wexford, Ireland) was used for HPLC. An ACE C_18_-PFP and Waters sunfire-C_18_ column 5 μm 143 Å column (250 mm × 10 mm) were used for semipreparative HPLC separations. 

### 4.2. Sesquiterpene Network

A dataset of 11,741 sesquiterpenes was collected from the ChEMBL database, Binding DB, and publications (Supplementary Material). Duplicate compounds were removed. Among them, anti-HBV activity was represented according to IC_50_ value; 152 compounds (agonists) in the data set were active (IC_50_ < 10 µM), and were tagged with “1”. Other compounds were marked with “0” (inactive). 

Skeleton type was determined for each sesquiterpene. Similarities among sesquiterpenes were calculate by the ChemmineR and ChemmineBO to afford a matrix of similarity. Based on a threshold value of similarity (90%), a network of sesquiterpenes was generated with “ggnet” in R, and nodes in the network were divided into different communities by cluster_louvain.

### 4.3. Plant Material

The roots of *S. lappa* were collected in Lijiang county, Yunnan province, China, and identified by Associate Professor Wu Zhikun (School of Pharmacy, Guizhou University of Chinese Medicine). A voucher (KUMST-BS-0007) specimen was deposited in the Laboratory of Chemical Biology for Natural Medicines, School of Life Science and Technology, Kunming University of Science and Technology. 

### 4.4. Extraction and Isolation

The air-dried and powdered roots of *S. lappa* (119.0 kg) were extracted by EtOH at room temperature. After removal of the solvent under reduced pressure, the crude extract (13.4 kg) was suspended in EtOAc (10 L) and partitioned with H_2_O (5 × 10 L). The EtOAc extract (4.4 kg) was subjected to MCI-gel CHP-20P column, eluting with a MeOH–H_2_O (40–100%) to give fourteen fractions, i.e., A–N. Fr.K (53.7 g) was subjected to a MCI-gel CHP-20P column using a step gradient of MeOH–H_2_O (35–100%) to afford eighteen fractions. Fr.K1–Fr.K18. Fr.K11 (19.3 g) was separated by RP-18 using a step gradient of MeOH–H_2_O (40–100%) to afford nine subfractions, i.e., Fr.K11a–Fr.K11i. Fr.K11d (7.3 g) was subjected to a silica gel column using a step gradient of *n*-hexane–EtOAc (96:4 to 80:20) to afford fourteen fractions, i.e., Fr.K11d1–Fr.K11d14. Fr.K11d4 (221.0 mg) was separated by RP-18 using a step gradient of MeOH–H_2_O (30–45%) to afford seven subfractions, i.e., Fr.K11d4a–Fr.K11d4g. Fr.K11d4c (69.9 mg) was purified with p–HPLC (CH_3_CN–H_2_O, 30:70, *v*/*v*) to afford **1** (25.7 mg). Fr.K12 (12.5 g) was separated by RP-18 using a step gradient of MeOH–H_2_O (50–100%) to afford nine subfractions, i.e., Fr.K12a–Fr.K12i. Fr.K12e (1.7 g) was subjected to a silica gel column using a step gradient of *n*-hexane–EtOAc (96:4 to 80:20) to afford six fractions, i.e., Fr.K12e1–Fr.K12e6. Fr.K12e3 (483.0 mg) was purified with p–HPLC (CH_3_CN–H_2_O, 52:48, *v*/*v*) to afford **9** (0.5 mg), **8** (5.3 mg). Fr.K12f (800.5 mg) was subjected to a silica gel column using a step gradient of *n*-hexane–EtOAc (96:4 to 80:20) to afford eleven fractions, i.e., Fr.K12f1–Fr.K12f11. Fr.K12f4 (23.8 mg) was purified with p–HPLC (CH_3_CN–H_2_O, 45:55, *v*/*v*) to afford **3** (10.6 mg), **5** (4.7 mg). Fr.K12f6 (22.1 mg) was purified with p–HPLC (CH_3_CN–H_2_O, 45:55, *v*/*v*) to afford **6** (12.2 mg). Fr.K12f8 (72.5 mg) was purified with p–HPLC (CH_3_CN–H_2_O, 45:55, *v*/*v*) to afford **7** (54.5 mg). Fr.K12g (494.1 mg) was subjected to a silica gel column using a step gradient of petroleum ether–EtOAc (97:3 to 91:9) to afford eight fractions, i.e., Fr.K12g1–Fr.K12g8. Fr.K12g4 (106.8 mg) was purified with p–HPLC (CH_3_CN–H_2_O, 60:40, *v*/*v*) to afford **4** (87.2 mg). Fr.I (88.7 g) was separated by RP–18 using a step gradient of MeOH–H_2_O (30–100%) to afford eighteen subfractions, i.e., Fr.I1–Fr.I18. Fr.I4 (734.0 mg) was separated by sephadex LH–20 with MeOH (100%) to give fourteen subfractions, i.e., Fr.I4a–Fr.I4n. Fr.I4a (87.6 mg) was separated by RP–18 using a step gradient of MeOH–H_2_O (30–90%) to afford twelve subfractions, i.e., Fr.I4a1–Fr.I4a12. Fr.I4a7 (2.1 mg) was purified with p–HPLC (CH_3_CN–H_2_O, 34:66, *v*/*v*) to afford **11** (0.4 mg). Fr.I7 (6.7 g) was separated by sephadex LH–20 with MeOH (100%) to give eight subfractions, i.e., Fr.I7a–Fr.I7h. Fr.I7a (498.0 mg) was separated by RP–18 using a step gradient of MeOH–H_2_O (30–90%) to afford fourteen subfractions, i.e., Fr.I7a1–Fr.I7a14. Fr.I7a6 (143.1 mg) was purified by p–HPLC followed with PTLC to afford **13** (2.6 mg). Fr.I7a8 (17.2 mg) was purified by p–HPLC followed with PTLC to afford **15** (0.6 mg), **14** (1.4 mg). Fr.I10 (9.8 g) was separated by RP–18 using a step gradient of MeOH–H_2_O (45–90%) to afford nine subfractions, i.e., Fr.I10a–Fr.I10i. Fr.I10g was subjected to a silica gel column using a step gradient of petroleum ether–EtOAc (8:1 to 2:1) to afford **10** (5.0 g). Fr.I10e was subjected to a silica gel column using a step gradient of petroleum ether–EtOAc (12:1 to 0:1) to afford thirteen fractions, i.e., Fr.I10e1–Fr.I10e13. Fr.I10e5 (222.0 mg) was purified with p–HPLC (CH_3_CN–H_2_O, 40:60, *v*/*v*) to afford **2** (180.3 mg). Fr.I10e8 (534.6 mg) was purified with p–HPLC (CH_3_CN–H_2_O, 39:61, *v*/*v*) to afford **12** (0.7 mg).

*Lappaterpene A* (**1**): pale yellow oily substance; ${\left[\alpha \right]}_{D}^{23.1}$ −30.1 ± 0.12 (*c* 0.3, MeOH); UV (MeOH) *λ*_max_ (log *ε*): 196 (0.469) nm; IR (KBr) *ν*_max_: 3525, 3078, 2981, 2934, 2861 cm**^−^**^1^; ^1^H and ^13^C NMR data, see Table 1; HREIMS: *m*/*z* 243.1367 [M + Na]^+^ (calcd for C_14_H_20_O_2_Na).

*Lappaterpene B* (**2**): needle colorless crystal; ${\left[\alpha \right]}_{D}^{23.2}$ 12.2 ± 0.31 (*c* 0.15, MeOH); UV (MeOH) *λ*_max_ (log *ε*): 196 (0.260) nm; IR (KBr) *ν*_max_: 3413, 3082, 2973, 2928, 2868 cm**^−^**^1^; ^1^H and ^13^C NMR data, see Table 1; HREIMS: *m/z* 261.1816 [M + Na]^+^ (calcd for C_15_H_26_O_2_Na).

*Lappaterpene C* (**3**): pale yellow oily substance; ${\left[\alpha \right]}_{D}^{22.2}$ −58.4 ± 0.10 (*c* 0.15, MeOH); UV (MeOH) *λ*_max_ (log *ε*): 282 (0.013), 196 (0.365), 253 (0.009), 192 (0.224) nm; IR (KBr) *ν*_max_: 3413, 3081, 2972, 2931, 2866 cm**^−^**^1^; ^1^H and ^13^C NMR data, see Table 1; HREIMS: *m/z* 275.2012 [M + Na]^+^ (calcd for C_16_H_28_O_2_Na).

*Lappaterpene D* (**4**): pale yellow oily substance; ${\left[\alpha \right]}_{D}^{22.6}$ −24.7 ± 0.07 (*c* 0.25, MeOH); UV (MeOH) *λ*_max_ (log *ε*): 196 (0.260) nm; IR (KBr) *ν*_max_: 3413, 3082, 2973, 2928, 2868 cm**^−^**^1^; ^1^H and ^13^C NMR data, see Table 1; HREIMS: *m/z* 289.3594 [M + Na]^+^ (calcd for C_17_H_30_O_2_Na). 

*Lappaterpene E* (**5**): pale yellow oily substance; ${\left[\alpha \right]}_{D}^{22.8}$ −29.4 ± 0.11 (*c* 0.15, MeOH); UV (MeOH) *λ*_max_ (log *ε*): 196 (0.636) nm; IR (KBr) *ν*_max_: 3436, 3103, 2972, 2932, 2867 cm**^−^**^1^; ^1^H and ^13^C NMR data, see Table 1; HREIMS: *m/z* 289.1780 [M + Na]^+^ (calcd for C_16_H_26_O_3_Na).

*Lappaterpene F* (**6**): pale yellow oily substance; ${\left[\alpha \right]}_{D}^{22.5}$ +0.32 ± 0.19 (*c* 0.15, MeOH); UV (MeOH) *λ*_max_ (log *ε*): 196 (0.413) nm; IR (KBr) *ν*_max_: 3408, 3087, 2967, 2926, 2874 cm**^−^**^1^; ^1^H and ^13^C NMR data, see Table 2; HREIMS: *m/z* 261.1856 [M + Na]^+^ (calcd for C_15_H_26_O_2_Na). 

*Lappaterpene G* (**7**): pale yellow oily substance; ${\left[\alpha \right]}_{D}^{23.4}$ +3.18 ± 0.09 (*c* 0.25, MeOH); UV (MeOH) *λ*_max_ (log *ε*): 196 (0.405), 192 (0.311) nm; IR (KBr) *ν*_max_: 3386, 3085, 2966, 2924, 2857 cm**^−^**^1^; ^1^H and ^13^C NMR data, see Table 2; HREIMS: *m/z* 261.1841 [M + Na]^+^ (calcd for C_15_H_26_O_2_Na).

### 4.5. ECD Calculation

The aglycons of the compounds were used as the chemical models to carry out ECD calculations. A conformation analysis was carried out using molecular mechanics MMFF. The resulting conformers (<15 KJ/mol) were optimized using DFT at the B3LYP-SCRF/6-311G(d,p) level using the integral equation formalism variant of the polarizable continuum model (IEF-PCM). All the calculations were run with Gaussian 09 [32]. The free energies and vibrational frequencies were calculated at the same level to confirm their stability, and no imaginary frequencies were found. The optimized low energy conformers with energy < 2 Kcal/mol were considered for ECD calculations. The TD-DFT/B3LYP-SCRF/6-311G(d,p) method was applied to calculate the excited energies, oscillator strength and rotational strength. The excited energies and rotational strength were used to simulate ECD spectra of each conformer by introducing the Gaussian Function. The final ECD spectrum of each compound was obtained by averaging all the simulated ECD spectra of all conformers according to their excited energies and Boltzmann distribution.

### 4.6. Anti-HBV Activity Evaluation

HepG2.2.15 cells, a human cancer cell line, were obtained from China Center for Type Culture Collection (Wuhan, China) and maintained in supplemented with 10% fetal bovine serum (meilunbio, Dalian, China), and 380 µg/mL G418 in a humidified 5% CO_2_ atmosphere at 37 °C. The inhibition to HBsAg and HBeAg was detected by ELISA.

HepG2.2.15 cells were seeded in 96-well plates and treated with compounds for 6 days. On day 3, the culture medium containing compounds was collected and replaced. The levels of HBsAg and HBeAg from cell culture supernatant were measured by HBsAg and HBeAg ELISA kits (Kehua, Shanghai, China), according to the manufacturer’s instructions. Lamivudine (Adamas, Shanghai, China) was tested as the positive control for anti HBV.

### 4.7. Cytotoxic Activity Evaluation

HepG2 cells were plated in 96-well plates in 100 μL medium (meilunbio, Dalian, China), to which the test samples were added at varied concentrations. After 72 h of incubation, MTT [[3-(4,5-dimethylthiazol-2-yl)-2,5-diphenyl tetrazolium bromide] solution [0.5 mg/mL in phosphate buffered saline (PBS)] was added (20 μL/well) [33], and the incubation continued for another 4 h to give a formazan product. In each well, 200 μL DMSO was added after the medium had been removed. Then, the formazan product is completely dissolved by sufficient oscillation. The absorbance of the solution was measured at 490 nm using a microplate reader (Tecan, Mendov, Switzerland). MTT is reduced by dehydrogenase activities in cells to give a purple formazan dye. The amount of the formazan dye generated by dehydrogenases in cells is directly proportional to the number of living cells. Compound concentrations reducing the viability of HepG2 cells culture by 50% (CC_50_) were calculated by regression analysis of the dose-response curves.

## Figures and Tables

**Table 1:** ^1^H and ^13^C NMR data for Compounds **1** and **2** (MeOH-*d*_4_), 600 MHz, *J* in Hz).

	1		2	
Position	*δ*_C_	*δ*_H_ (*J* in Hz)	*δ*_C_	*δ*_H_ (*J* in Hz)
1a	47.1 (CH)	2.86 (ddd, 8.5, 6.2, 2.1)	41.8 (CH_2_)	1.36 ^a^
1b	-	-	-	1.09 ^b^
2a	28.5 (CH_2_)	1.78 (ddd, 12.9, 9.8, 8.5)	17.9 (CH_2_)	1.36 ^a^
2b	-	2.07 (ddd, 12.9, 6.2, 3.6)	-	1.88 (m)
3a	30.3 (CH_2_)	2.40 (ddd, 17.9, 9.8, 3.6)	40.9 (CH_2_)	1.66 (brd, 15.9)
3b		2.43 ^b^	-	1.36 ^a^
4	151.7 (C)		71.0 (C)	-
5	54.7 (CH)	2.71 ^a^	52.1 (CH)	1.09 ^b^
6a	69.5 (CH)	3.55 (t, 9.6)	26.3 (CH_2_)	1.44 (d, 13.1)
6b	-	-	-	1.74 (ddd, 13.1, 4.1, 2.0)
7	61.4 (CH)	2.71 ^a^	42.3 (CH)	2.02 (dd, 12.2, 4.1)
8a	31.1 (CH_2_)	1.94 (dddd, 13.6, 5.9, 4.5, 3.1)	27.4 (CH_2_)	1.51 (brdd, 12.7, 3.8)
8b		1.51 (dtd, 13.6, 11.9, 3.4)	-	1.89 (m)
9a	34.3 (CH_2_)	2.12 (ddd, 12.3, 4.5, 3.4)	43.9 (CH_2_)	1.34 (dd, 12.8, 3.8)
9b	-	2.43 ^b^	-	1.18 (dd, 12.8, 4.2)
10	151.9 (C)	-	33.6 (C)	-
11	212.9 (C)	-	154.2 (C)	-
12	-	-	63.8 (CH_2_)	4.06 (brd, 1.3)
13a	27.8 (CH_3_)	2.18 (s)	106.6 (CH_2_)	5.02 (d, 2.0)
13b	-	-	-	4.92 (d, 2.0)
14a	109.5 (CH_2_)	5.04 (d, 2.6)	17.9 (CH_3_)	1.13 (s)
14b	-	5.07 (d, 2.6)	-	
15a	110.0 (CH_2_)	4.88 (d, 1.8)	29.1 (CH_3_)	1.07 (s)
15b		4.77 (d, 1.8)		

*J*-values are in parentheses and are reported in Hz; chemical shifts are given in ppm; assignments were confirmed by COSY, 1D HSQC, and HMBC experiments. ^a^ Overlapped with each other; ^b^ Overlapped with each other.

**Table 2:** ^1^H and ^13^C NMR data for Compounds **3**–**5** (MeOH-*d*_4_, 600 MHz, *J* in Hz).

	3		4		5	
Position	*δ*_C_	*δ*_H_ (*J* in Hz)	*δ*_C_	*δ*_H_ (*J* in Hz)	*δ*_C_	*δ*_H_ (*J* in Hz)
1a	40.7 (CH_2_)	1.38 ^a^	40.7 (CH_2_)	1.50–1.36 ^a^	40.6 (CH_2_)	1.44 (m)
1b	-	1.09 ^b^	-	1.10 ^b^	-	1.22 (brdd, 12.2, 5.3)
2a	19.3 (CH_2_)	1.65 ^c^	19.4 (CH_2_)	1.56 ^c^	19.3 (CH_2_)	1.59 ^b^
2b	-	-	-	-	-	-
3a	36.2 (CH_2_)	1.77 ^e^	36.9 (CH_2_)	1.77 (ddd, 12.2, 3.2, 1.5)	35.9 (CH_2_)	1.50–1.43 ^a^
3b	-	1.44 ^d^	-	1.50–1.36 ^a^	-	1.72 (ddd, 12.2, 3.3, 1.5)
4	76.4 (C)	-	76.3 (C)	-	76.4 (C)	
5	51.1 (CH)	1.38 ^a^	51.2 (CH)	1.50–1.36 ^a^	50.5 (CH)	1.50–1.43 ^e^
6a	26.3 (CH_2_)	1.77 ^e^	26.3 (CH_2_)	1.50–1.36 ^a^	26.2 (CH_2_)	1.22 (m)
6b	-	1.22 (m)	-	1.21 (m)	-	1.81 (m)
7	41.9 (CH)	2.08 (ddd, 15.5, 10.5, 3.8)	41.9 (CH)	2.03 (ddd, 12.9, 4.1, 2.2)	40.3 (CH)	2.49 (m)
8a	27.2 (CH_2_)	1.65 ^c^	27.3 (CH_2_)	1.56 ^c^	27.2 (CH_2_)	1.59 ^b^
8b	-	1.40 (brdd, 12.3, 2.6)	-	1.50–1.36 ^a^	-	1.50–1.43 ^a^
9a	45.0 (CH_2_)	1.25 (brdd, 13.2, 3.9)	45.1 (CH_2_)	1.26 (brdd, 13.5, 4.4)	44.9 (CH_2_)	1.32 (m)
9b	-	1.44 ^d^	-	1.50–1.36 ^a^	-	1.50–1.43 ^a^
10	34.3 (C)	-	34.3 (C)	-	34.2 (C)	-
11	154.1 (C)	-	154.1 (C)	-	154.1 (C)	-
12	63.8 (CH_2_)	4.05 (brd, 1.4)	63.8 (CH_2_)	4.07 (brs)	169.8 (C)	-
13a	106.5 (CH_2_)	5.01 (d, 1.6)	106.5 (CH_2_)	5.04 (d, 1.6)	120.5 (CH_2_)	5.52 (brs)
13b	-	4.88 (d, 1.4)	-	4.91 (d, 1.6)	-	6.07 (brs)
14a	18.2 (CH_3_)	1.09 ^b^	18.3 (CH_3_)	0.97 (s)	18.3 (CH_3_)	0.97 (s)
14b	-	-	-	-	-	
15a	17.6 (CH_3_)	0.97 (s)	18.4 (CH_3_)	1.10 ^b^	18.1 (CH_3_)	1.08 (s)
15b						
OCH_3_	46.6	3.20 (s)	-	-	46.5	3.14 (s)
CH_2_			54.6 (CH_2_)	3.38 (m)		
			-	3.43 (m)		
CH_3_			15.2 (CH_3_)	1.09 (t, 8.3)		

*J*-values are in parentheses and reported in Hz; chemical shifts are given in ppm; assignments were confirmed by COSY, 1D HSQC, and HMBC experiments. ^a^ Overlapped with each other; ^b^ Overlapped with each other; ^c^ Overlapped with each other; ^d^ Overlapped with each other; ^e^ Overlapped with each other.

**Table 3:** ^1^H and ^13^C NMR data for Compounds **6** and **7** (in MeOH-*d*_4_).

6				7			
Position	Type	*δ*_C_	*δ*_H_ (*J* in Hz)	Position	Type	*δ*_C_	*δ*_H_ (*J* in Hz)
1	CH_2_	113.0	5.16 (dd, 10.9, 1.8)	1	CH_2_	65.1	3.58 (dd, 11.3, 4.1)
			5.29 (dd, 17.4, 1.8)				3.45 (dd, 11.3, 7.5)
2	CH	141.2	5.88 (dd, 17.4, 10.9)	2	C	149.0	
3	C	75.4		3	CH	75.2	4.10 (dd, 7.5, 4.1)
4	CH_2_	36.5	2.54 (m)	4	CH_2_	32.0	2.11 (m)
5	CH_2_	21.5	2.06 (m)				2.03 (m)
			1.98 (m)	5	CH_2_	26.2	2.08 (m)
6	CH	124.4	5.12 (m)				1.98 (m)
7	C	134.5		6	CH	124.0	5.15 (td, 7.0, 1.3)
8	CH_2_	39.5	1.98 (m)	7	C	134.9	
9	CH_2_	26.4	2.06 (m)	8	CH_2_	39.5	2.00 (m)
10	CH	124.0	5.08 (tdd, 5.7, 2.9, 1.5)	9	CH_2_	26.3	2.06 (m)
11	C	130.7					2.18 (m)
12	CH_2_	68.2	3.45 (dd, 10.8, 16.8)	10	CH	123.9	5.09 (td, 12.8, 2.9)
13	CH_3_	14.6	1.59 (s)	11	C	130.7	
14	CH_3_	16.4	1.59 (s)	12	CH_2_	109.7	5.09 (s)
15	CH_3_	24.5	1.66 (s)				4.91 (s)
				13	CH_3_	14.7	1.62 (s)
				14	CH_3_	16.4	1.61 (s)
				15	CH_3_	24.5	1.68 (s)

## Data Availability

Not applicable.

## Supplementary Materials

The following supporting information can be downloaded at: https://www.mdpi.com/article/10.3390/molecules27062023/s1, Tables S1–S3, Figures S1–S75, HRESI, CD, IR and NMR spectra of Compounds **1**–**7**; Tables S1–S3: The top ten square nodes, Classification of medicinal plants, Classification of *Saussurea* spp.

## Sample Availability

Samples of the compounds are available from the authors.
