# Supplementary material for: Structure-Based Molecular Networking for the Discovery of Anti-HBV Compounds from Saussurea lappa (Decne.) C.B Clarke
Source: Molecules. 2022 Mar 21;27(6):2023. doi: 10.3390/molecules27062023 (PMC8955460; doi:10.3390/molecules27062023)
Supplement: Supplementary file 1 [file molecules-27-02023-s001.zip › molecules-1640004-supplementary.pdf]

SUPPLEMENTARY MATERIAL

Structure-Based Molecular Networking for the Discovery of Anti-HBV compounds from *Saussurea lappa Saussurea lappa* (Decne.) C.B Clarke

Tao Wu,[1,2] Xin-Jian Yan,[1,2] Tian-Rong Yang,[1,2] Yun-fen Wang,[1]Jing-Yi He,[1] Yang Feng,[1] Hao Chen,[1,*] and Min Xu[1,*]

[1] Center for Pharmaceutical Sciences, Faculty of Life Science and Technology, Kunming University of Science and Technology, Chenggong Campus, Kunming 650500, P. R. China

[2] These authors contributed equally to this work

* Correspondence: chenhao@kust.edu.cn (H.C.), xumin@kust.edu.cn (M.X.);

Tel: +86 871 65920253

Supplementary material

Content	Page
Table S1. The top ten square nodes.	4
Table S2. Classification of medicinal plants.	5
Table S3. Classification of Saussurea DC	6
Figure S1 1H NMR spectrum of compound 1 (CD3OD, 600 MHz)	7
Figure S2. 13C NMR spectrum of compound 1 (CD3OD, 150 MHz)	8
Figure S3. ROESY spectrum of compound 1 (CD3OD, 600 MHz)	9
Figure S4. 1H-1H COSY spectrum of compound 1 (CD3OD, 600 MHz)	10
Figure S5. HSQC spectrum of compound 1 (CD3OD, 600 MHz)	11
Figure S6. HMBC spectrum of compound 1 (CD3OD, 600 MHz)	12
Figure S7. HRESI spectrum of compound 1	13
Figure S8. CD and UV spectrum of compound 1	14
Figure S9. IR spectrum of compound 1	15
Figure S10. [α] 25D of compound 1	16
Figure S11. 1H NMR spectrum of compound 2 (CD3OD, 600 MHz)	17
Figure S12. 13C NMR spectrum of compound 2 (CD3OD, 150 MHz)	18
Figure S13. ROESY spectrum of compound 2 (CD3OD, 600 MHz)	19
Figure S14. 1H-1H COSY spectrum of compound 2 (CD3OD, 600 MHz)	20
Figure S15. HSQC spectrum of compound 2 (CD3OD, 600 MHz)	21
Figure S16. HMBC spectrum of compound 2 (CD3OD, 600 MHz)	22
Figure S17. HRESI spectrum of compound 2	23
Figure S18. CD and UV spectrum of compound 2	24
Figure S19. IR spectrum of compound 2	25
Figure S20. [α] 25D of compound 2	26
Figure S21. X-ray crystallographic analysis for compound 2	27-29
Figure S22. 1H NMR spectrum of compound 3 (CD3OD, 600 MHz)	30
Figure S23. 13C NMR spectrum of compound 3 (CD3OD, 150 MHz)	31
Figure S24. ROESY spectrum of compound 3 (CD3OD, 600 MHz)	32
Figure S25. 1H-1H COSY spectrum of compound 3 (CD3OD, 600 MHz)	33
Figure S26. HSQC spectrum of compound 3 (CD3OD, 600 MHz)	34
Figure S27. HMBC spectrum of compound 3 (CD3OD, 600 MHz)	35
Figure S28. HRESI spectrum of compound 3	36
Figure S29. CD and UV spectrum of compound 3	37
Figure S30. IR spectrum of compound 3	38
Figure S31. [α] 25D of compound 3	39
Figure S32. 1H NMR spectrum of compound 4 (CD3OD, 600 MHz)	40
Figure S33. 13C NMR spectrum of compound 4 (CD3OD, 150 MHz)	41
Figure S34. ROESY spectrum of compound 4 (CD3OD, 600 MHz)	42
Figure S35. 1H-1H COSY spectrum of compound 4 (CD3OD, 600 MHz)	43
Figure S36. HSQC spectrum of compound 4 (CD3OD, 600 MHz)	44
Figure S37. HMBC spectrum of compound 4 (CD3OD, 600 MHz)	45

Figure S38. HRESI spectrum of compound 4	46
Figure S39. CD and UV spectrum of compound 4	47
Figure S40. IR spectrum of compound 4	48
Figure S41. [α] 25D of compound 4	49
Figure S42. 1H NMR spectrum of compound 5 (CD3OD, 600 MHz)	50
Figure S43. 13C NMR spectrum of compound 5 (CD3OD, 150 MHz)	51
Figure S44. ROESY spectrum of compound 5 (CD3OD, 600 MHz)	52
Figure S45. 1H-1H COSY spectrum of compound 5 (CD3OD, 600 MHz)	53
Figure S46. HSQC spectrum of compound 5 (CD3OD, 600 MHz)	54
Figure S47. HMBC spectrum of compound 5 (CD3OD, 600 MHz)	55
Figure S48. HRESI spectrum of compound 5	56
Figure S49. CD and UV spectrum of compound 5	57
Figure S50. IR spectrum of compound 5	58
Figure S51. [α] 25D of compound 5	59
Figure S52. 1H NMR spectrum of compound 6 (CD3OD, 600 MHz)	60
Figure S53. 13C NMR spectrum of compound 6 (CD3OD, 150 MHz)	61
Figure S54. ROESY spectrum of compound 6 (CD3OD, 600 MHz)	62
Figure S55. 1H-1H COSY spectrum of compound 6 (CD3OD, 600 MHz)	63
Figure S56. HSQC spectrum of compound 6 (CD3OD, 600 MHz)	64
Figure S57. HMBC spectrum of compound 6 (CD3OD, 600 MHz)	65
Figure S58. HRESI spectrum of compound 6	66
Figure S59. CD and UV spectrum of compound 6	67
Figure S60. IR spectrum of compound 6	68
Figure S61. [α] 25D of compound 6	69
Figure S62. 1H NMR spectrum of compound 7 (CD3OD, 600 MHz)	70
Figure S63. 13C NMR spectrum of compound 7 (CD3OD, 150 MHz)	71
Figure S64. ROESY spectrum of compound 7 (CD3OD, 600 MHz)	72
Figure S65. 1H-1H COSY spectrum of compound 7 (CD3OD, 600 MHz)	73
Figure S66. HSQC spectrum of compound 7 (CD3OD, 600 MHz)	74
Figure S67. HMBC spectrum of compound 7 (CD3OD, 600 MHz)	75
Figure S68. HRESI spectrum of compound 7	76
Figure S69. CD and UV spectrum of compound 7	77
Figure S70. IR spectrum of compound 7	78
Figure S71. [α] 25D of compound 7	79
Figure S72. 1H NMR spectrum of compound 10 (CD3OD, 600 MHz)	80
Figure S73. 13C NMR and DEPT spectrum of compound 6 (CD3OD, 150 MHz)	81
Figure S74. ESI+ spectrum of compound 10	82
Figure S75. X-ray crystallographic analysis for compound 10	83-85

Table S1. The top ten square nodes.

Number	Degree	Smiles	Skeleton _types
45	27	C(C)(C)C1CC2C(C)(CC1)CCCC2C	Eudesmane
21	21	C(C)(C)C1CC2C(C)CCC2C(C)CC1	Guaiane
26	20	C(C)(C)C1C2C(C(C)CC1)CCC(C)C2	Cadinane
54	18	CC1CCCC(C)CCC(C(C)C)CC1	Germacane
40	16	C(CCCC(C)C1CCC(C)CC1)(C)C	Bisabolane
9	16	C(C)(C)C1CCC(CCCC)C(C)CC1	secoguaiane
27	15	C(C)(C)C1C2C(C(C)CC1)CC(C)CC2	Lasserane
17	15	C(C)(C)C1CC2C(C)(CCC2CC)CC1	Iphionane
87	14	CC(CC1)CC21CC(C(C)C)CCC2C	Spiroaxane
73	13	CC1C(C)CCC(C)C2C(C(C)CC2)C1	Dumortane

Table S2. Classification of medicinal plants.

No	Skeleton	Skeleton_SMILES	Sources_A	Sources_B	Sources_C	Sources_D	Sources_E
21	Guaiane	C(C)(C)C1CC2C(C)CC C2C(C)CC1	*Saussurea* DC	*Artemisia L.*	*Mikania Willd*	*Glebionis Cass.*	*Anthemis L.*
54	Germacane	CC1CCCC(C)CCC(C(C)C)CC1	*Saussurea* DC	*Inula L.*	*Carpesium L.*	*Pseudelephanto pus Rohr.*	*Helianthus .*
45	Eudesmane	C(C)(C)C1CC2C(C)(C C1)CCCC2C	*Saussurea* DC	*Tugarinovia Iljin*	*Inula L.*	*Atractylodes*	*Tanacetum L.*
40	Bisabolane	C(CCCC(C)C1CCC(C) CC1)(C)C	*Ligusticum L.*	*Eupatorium L.*	*Cremanthodium Benth*	*Artemisia L.*	*Matricaria*
26	Cadinane	C(C)(C)C1C2C(C(C)C C1)CCC(C)C2	*Pteridum*	*Pulicaria Gaertn.*	*Eupatorium L.*	*Artemisia L.*	*Cyperus L.*
22	Carotane	CC1(CCC2C(C)C)C2C CC(C)CC1	*Inula L.*	*Ageratum L.*	*Ferula L.*	*Sium L.*	*Daucus L.*
63	Caryophyll ane	CC1(C)C2C(C(C)CCC C(C)CC2)C1	*Dacrydium pierrei Hickel*	*Juniperus formosana Hayata*	*Sindora Miq.*	*Gossypium L.*	*Pulicaria Gaertn.*
44	Prezizaane	CC(CC1)C2(CC3)C1C(C)(C)C(C)C3C2	*Juniperus L.*	*Neocallitropsis*	*Vetiveria*	*Illicium L.*	*Streptomyces*
119	Patchoulane	CC1CCC(C2(C)C)C3(C)C1CC2CC3	*Pogostemon helferi*	*Cyperus L.*	*Croton crassifolius.*	*Daphne L.*	*Agastache Clayt. in Gronov*
50	Eremophila ne	C(C)(C)C1CC2(C)C(C) CCCC2CC1	*Senecio L.*	*Solidago L. .*	*Ligularia Cass.*	*Litsea*	*Pedicularis L.*

Table S3. Classification of *Saussurea* DC

NAME	Number of documents	Skeleton_number	Skeleton_types
Saussurea cauloptera	1	2	Eudesmane、Serpentine
Saussurea deltoidei	1	2	Guaiane、elemane
Saussurea laniceps	2	2	Guaiane、Germacrane
Saussurea lappa	4	3	Eudesmane、Guaiane、Germacrane
Saussurea macrota	1	2	Guaiane、Germacrane
Saussurea medusa	2	2	Eudesmane、Cadinene)
Saussurea medusa Maxim	1	2	Eudesmane、Amyrane
Saussurea pulchella	1	2	Guaiane、Germacrane
Saussureae Radix	1	2	Eudesmane、Guaian

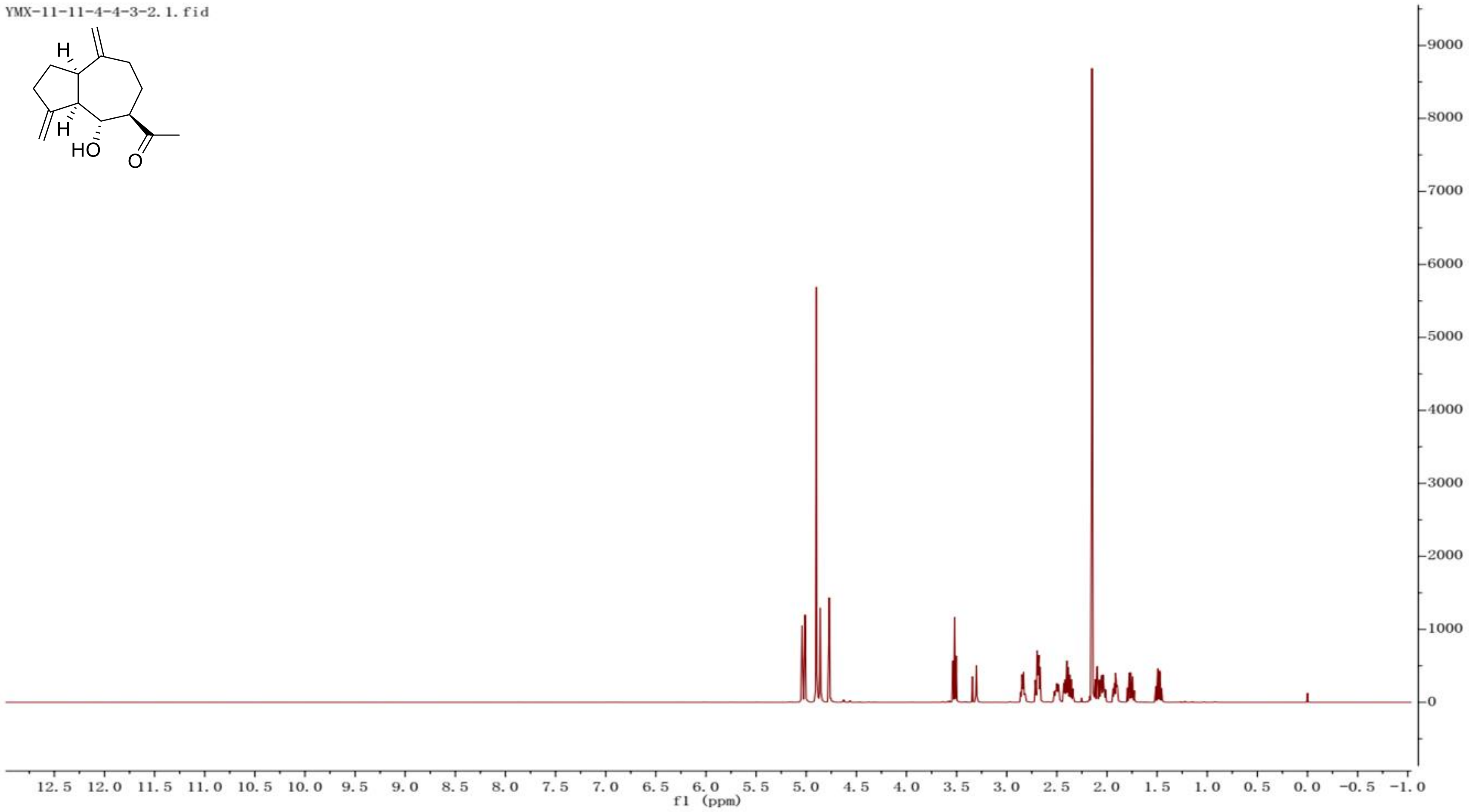

Figure S1. ^{1}H NMR spectrum of compound **1** (CD_3OD, 600 MHz)

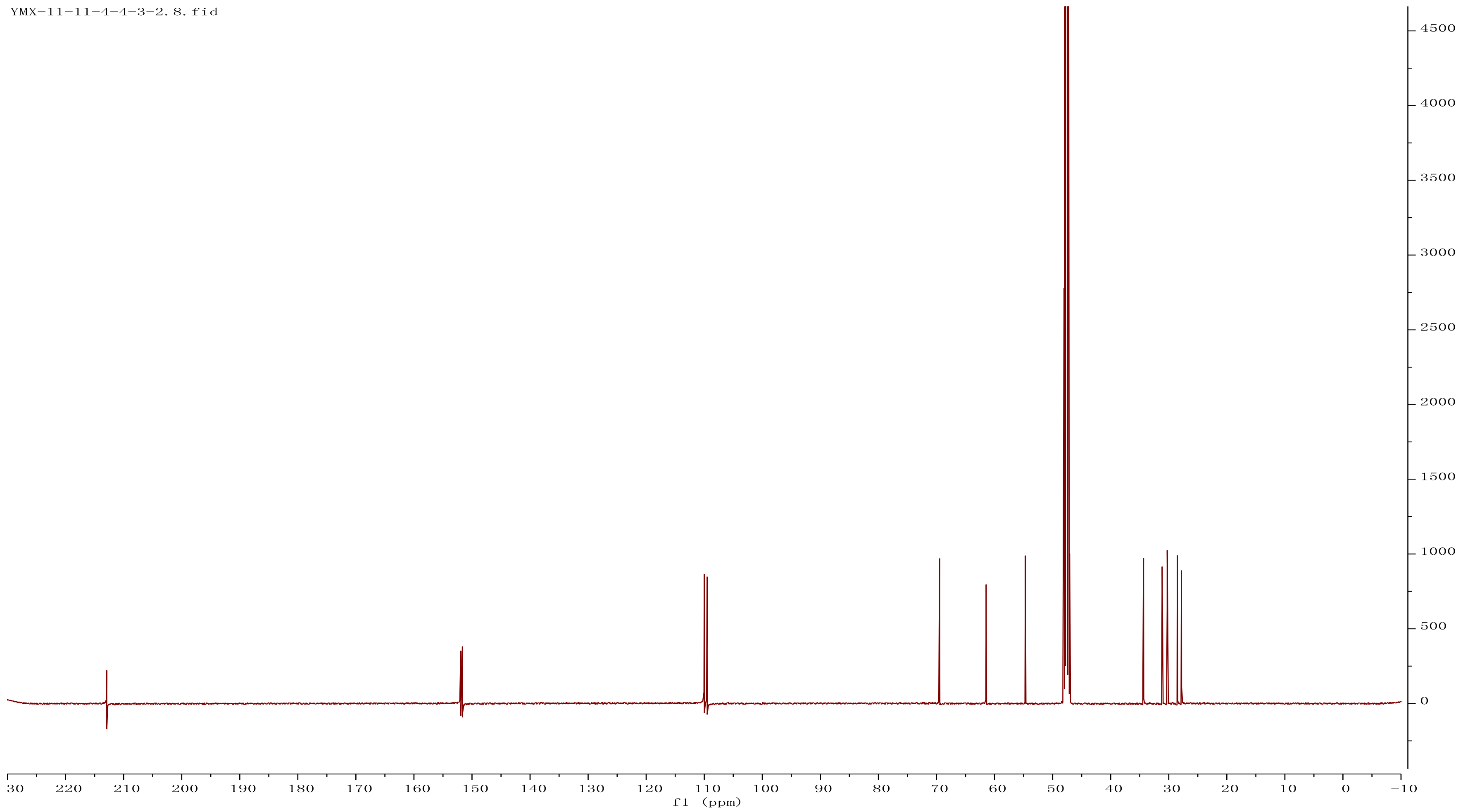

Figure S2. ^{13}C NMR spectrum of compound **1** (CD_3OD, 150 MHz)

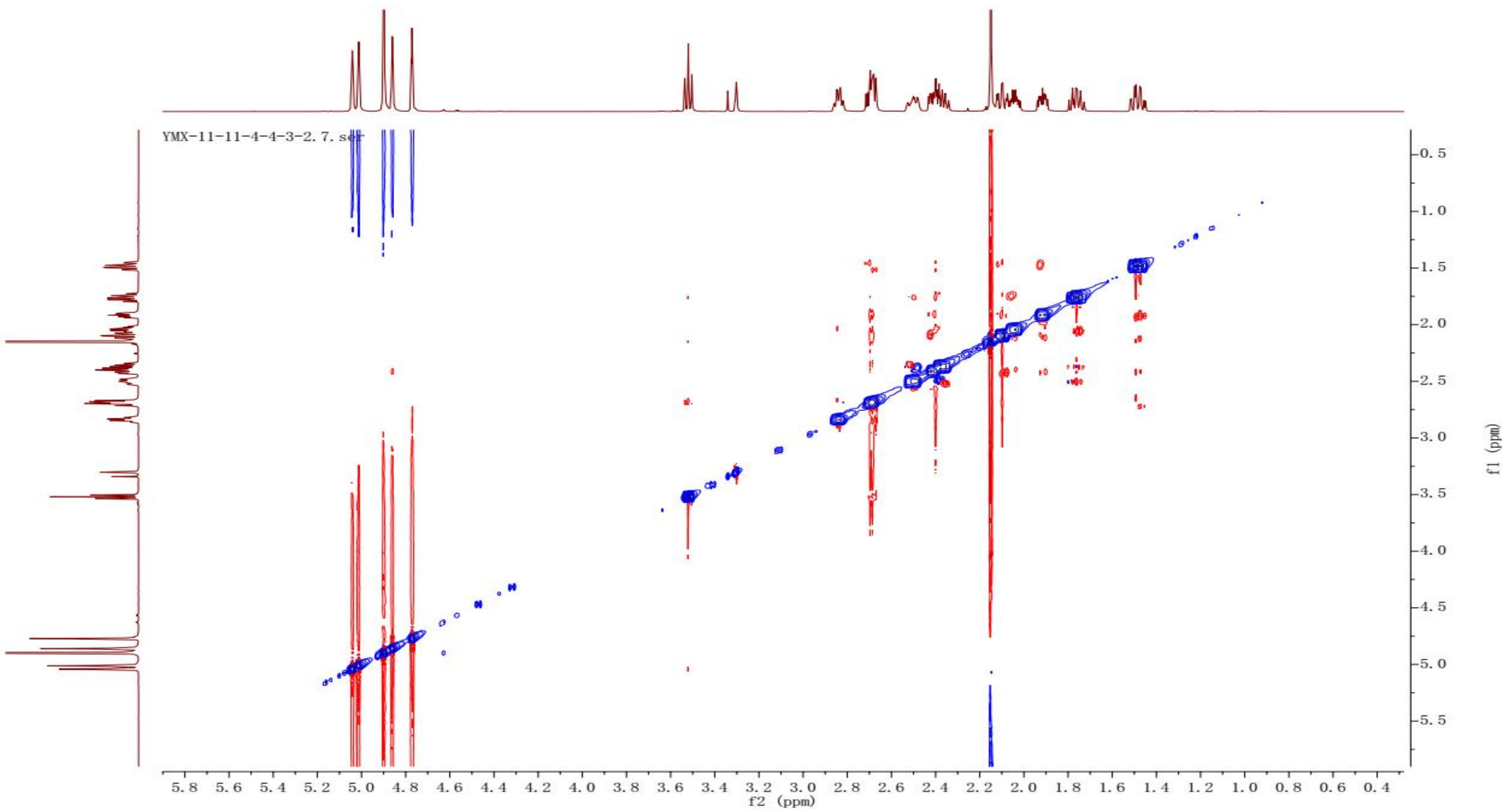

Figure S3. ROESY spectrum of compound **1** (CD_3OD, 600 MHz)

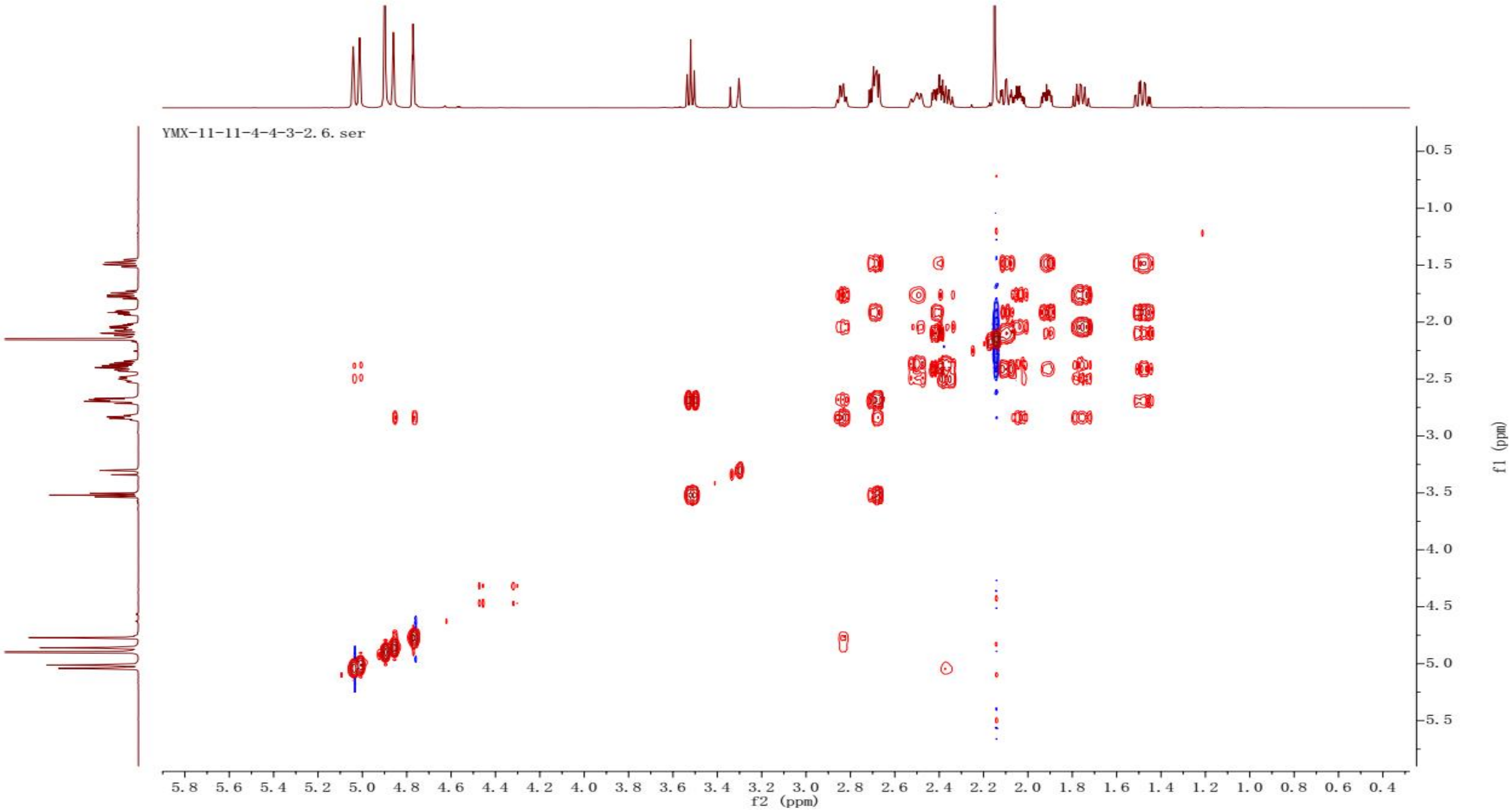

Figure S4. ^1H-^1H COSY spectrum of compound **1** (CD_3OD, 600 MHz)

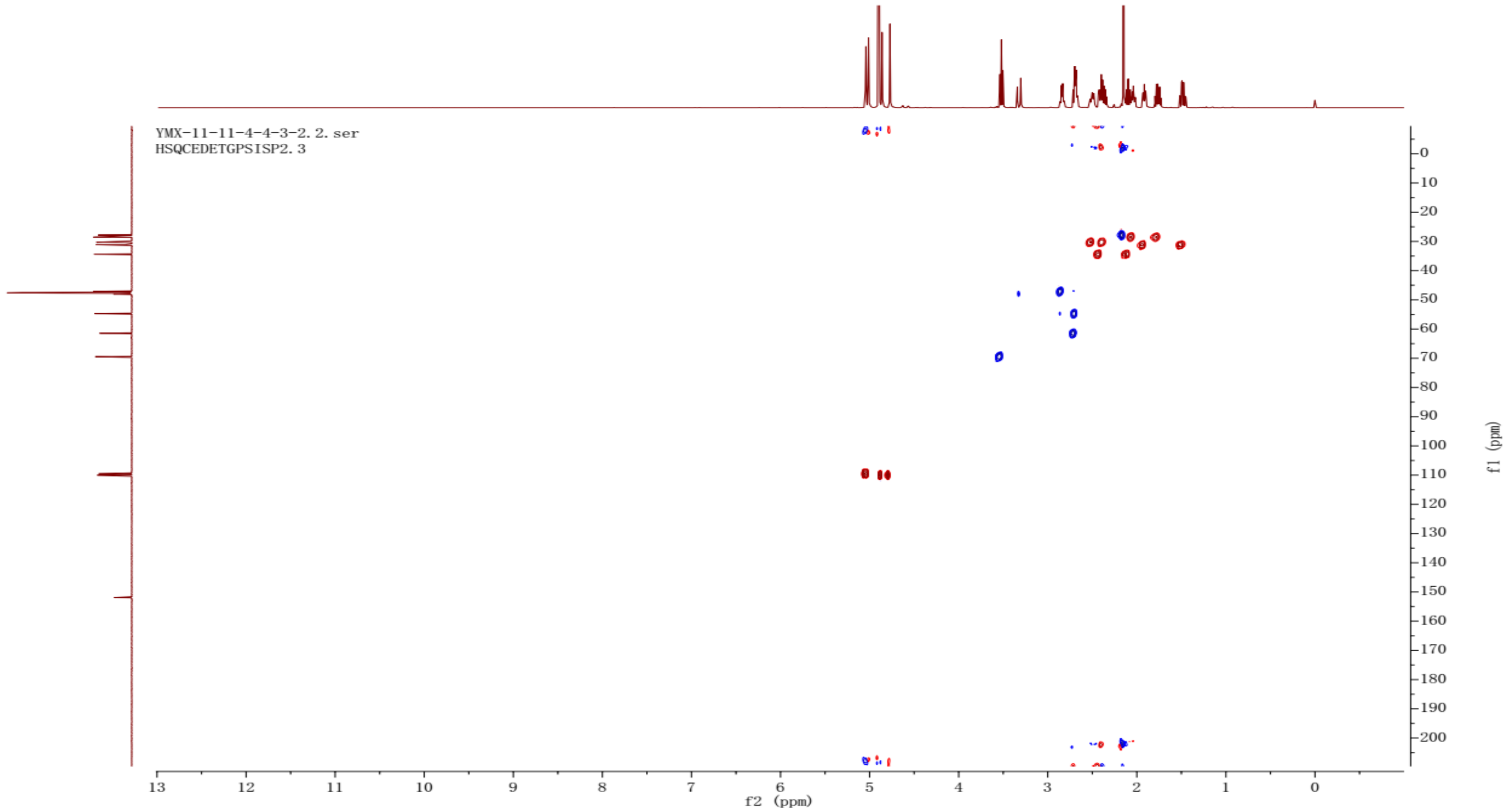

Figure S5. HSQC spectrum of compound **1** (CD_3OD, 600 MHz)

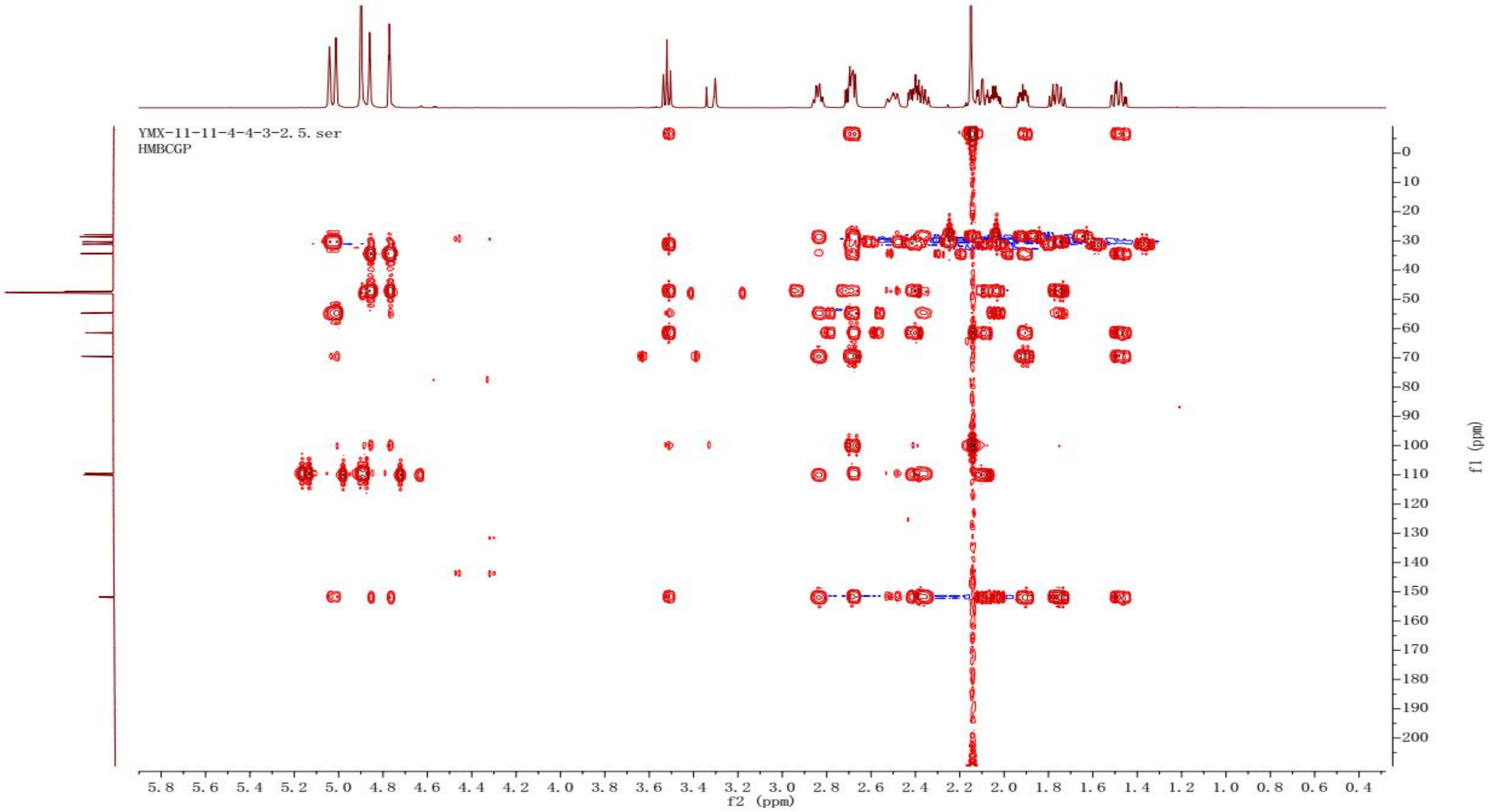

Figure S6. HMBC spectrum of compound **1** (CD_3OD, 600 MHz)

Qualitative Analysis Report

**Data Filename**	YMX-11-11-4-4-3-2.d	**Sample Name**	YMX-11-11-4-4-3-2
**Sample Type**	Sample	**Position**	p1b2
**Instrument Name**	Instrument 1	**User Name**	Q-TOF-HP\Q-TOF
**Acq Method**	default20190111-ms.m	**Acquired Time**	1/12/2020 12:37:26 AM
**IRM Calibration Status**	Success	**DA Method**	1.m
**Comment**			

Sample Group **Info.**
Acquisition SW 6200 series TOF/6500 series
Version Q-TOF B.05.01 (B5125.3)

User Chromatograms

Fragmentor Voltage 155 **Collision Energy** 0 **Ionization Mode** ESI

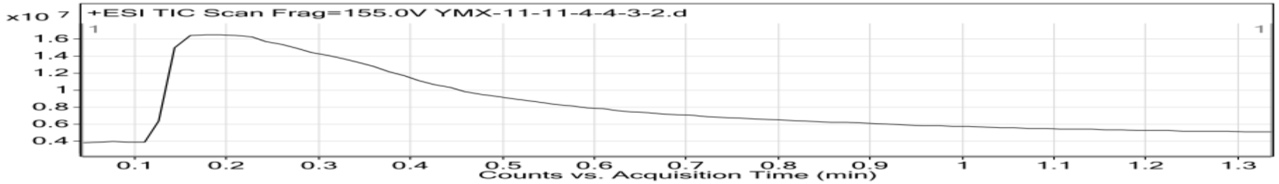

User Spectra

Fragmentor Voltage 155 **Collision Energy** 0 **Ionization Mode** ESI

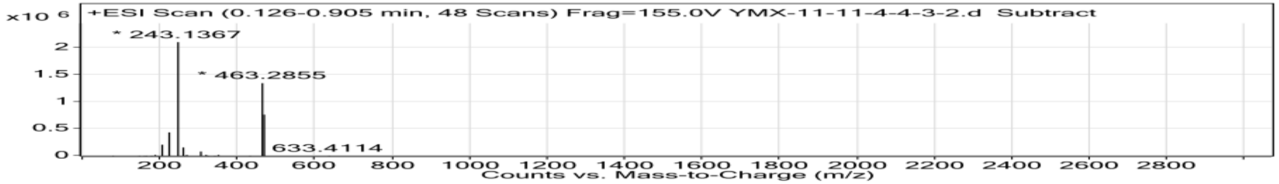

Peak List

m/z	z	Abund
203.1421	1	217932.42
221.1527	1	446446.97
243.1367	1	2108812
243.2699		151521.28
244.1382	1	518419.28
259.1082	1	160975.52
463.2855	1	1355956.25
463.464	1	123497.32

Agilent Technologies Page 1 of 2 Printed at: 4:30 PM on: 4/15/2020

Figure S7. HRESI spectrum of compound **1**

YMX-11-11-4-4-3-2

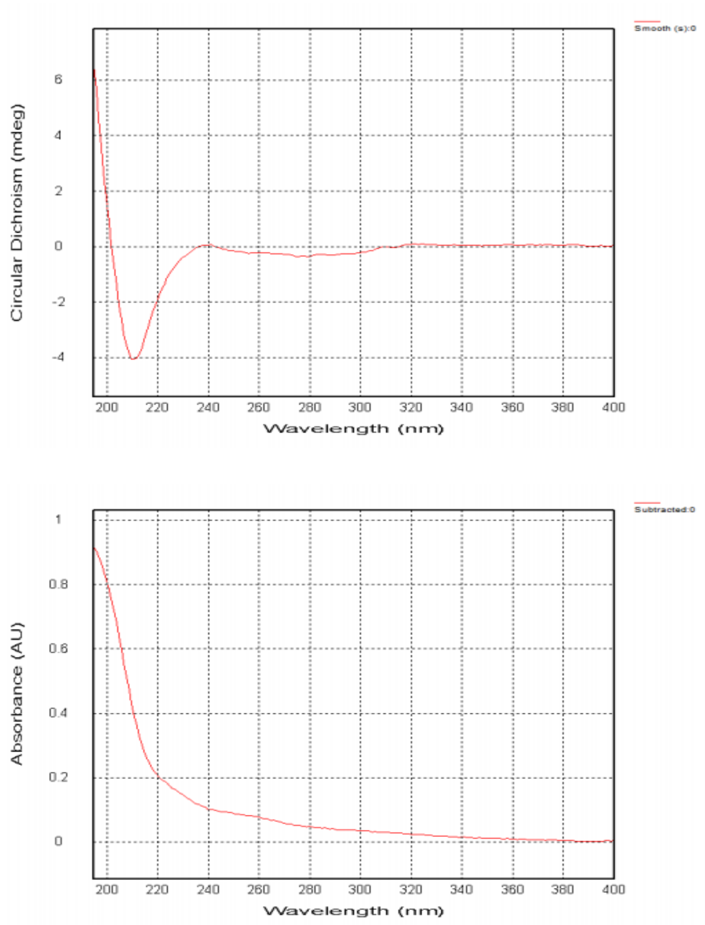

File: YMX-11-11-4-4-3-2 (195-400 nm) 20112718.dsx
ProBinaryX
Attributes :
- Time Stamp :Fri Nov 27 19:51:12 2020

- File ID : {A8234499-FCB9-4b69-BBF6-E4E19927A8D4}
- Is CFR Compliant : false
- Original data has not been modified.

Remarks:
- User: CD
- Date: 2020/11/27
- Instrument: 0547
- DetectorType: LAAPD
- DichOS Calibration Correction Curve: 0547/2
- HV (CDDC channel): 0 v
- Time per point: 1 s
- Description: Sample 1
- Concentration: 0.1680mg/mL MeOH
- Pathlength: 1 mm
- Temperature: 20℃

Settings:
- Time-per-point: 1s (25us x 40000)
- SE
- Wavelength: 195nm - 400nm
- Step Size: 1nm
- Bandwidth: 1nm

Figure S8. CD and UV spectrum of compound **1**

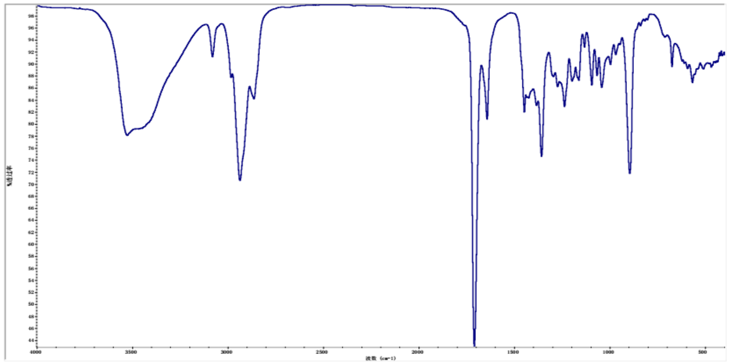

Sample Name: YMX-11-11-4-4-3-2
KBr压片
采集时间: 星期三 10月 28 16:21:54 2020 (GMT+08:00)
仪器型号: NICOLET iS10
Software version: OMNIC 9.8.372

样品扫描次数: 16
背景扫描次数: 16
分辨率: 4.000
采样增益: 1.0
动镜速度: 0.4747
光阑: 80.00

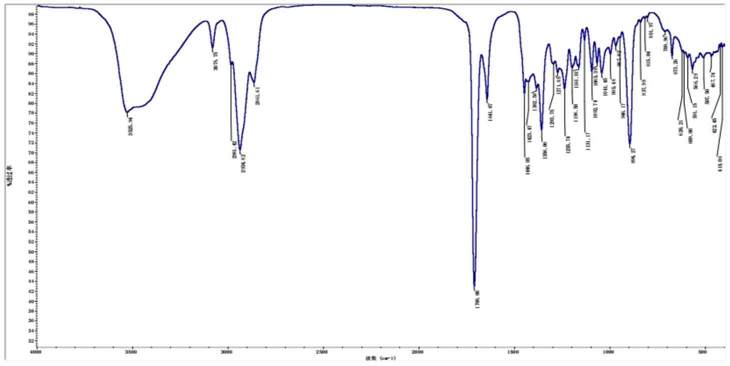

Sample Name: YMX-11-11-4-4-3-2
KBr压片
采集时间: 星期三 10月 28 16:21:54 2020 (GMT+08:00)
仪器型号: NICOLET iS10
Software version: OMNIC 9.8.372

样品扫描次数: 16
背景扫描次数: 16
分辨率: 4.000
采样增益: 1.0
动镜速度: 0.4747
光阑: 80.00

星期三 10月 28 16:28:45 2020 (GMT+08:00)
标峰:
谱图: YMX-11-11-4-4-3-2
范围: 4000.00 400.00
（绝对）阈值: 97.507
灵敏度: 99
峰表:

峰位置:	410.06	强度:	91.307
峰位置:	420.72	强度:	91.303
峰位置:	435.26	强度:	90.300
峰位置:	444.53	强度:	90.177
峰位置:	467.74	强度:	89.349
峰位置:	507.50	强度:	89.121
峰位置:	566.29	强度:	86.817
峰位置:	591.15	强度:	89.154
峰位置:	609.00	强度:	90.052
峰位置:	620.21	强度:	90.420
峰位置:	673.26	强度:	89.510
峰位置:	709.96	强度:	94.430
峰位置:	801.97	强度:	97.265
峰位置:	815.04	强度:	97.097
峰位置:	837.99	强度:	96.283
峰位置:	894.27	强度:	71.654
峰位置:	946.17	强度:	93.158
峰位置:	967.82	强度:	91.498
峰位置:	995.48	强度:	89.847
峰位置:	1041.45	强度:	86.025
峰位置:	1065.38	强度:	88.027
峰位置:	1092.74	强度:	86.427
峰位置:	1131.17	强度:	92.740
峰位置:	1161.01	强度:	87.220
峰位置:	1196.50	强度:	87.110
峰位置:	1235.74	强度:	82.836
峰位置:	1271.85	强度:	86.159
峰位置:	1293.75	强度:	87.843
峰位置:	1356.06	强度:	74.534
峰位置:	1382.58	强度:	83.024
峰位置:	1423.47	强度:	84.289
峰位置:	1446.05	强度:	81.823
峰位置:	1641.07	强度:	80.757
峰位置:	1708.00	强度:	42.918
峰位置:	2861.61	强度:	84.143
峰位置:	2934.82	强度:	70.524
峰位置:	2981.42	强度:	87.694
峰位置:	3078.75	强度:	91.125
峰位置:	3525.94	强度:	78.053

Figure S9. IR spectrum of compound **1**

Rudolph Research Analytical

This sample was measured on an Autopol VI, Serial #91058
Manufactured by Rudolph Research Analytical, Hackettstown, NJ, USA.

Measurement Date : Thursday, 26-NOV-2020

Set Temperature : OFF

Time Delay : Disabled

Delay between Measurement : Disabled

n	Average	Std.Dev.	% RSD	Maximum	Minimum
5	-30.07	0.12	-0.39	-29.90	-30.20

S.No	Sample ID	Time	Result	Scale	OR °Arc	WLG.nm	Lg.mm	Conc.g/100ml	Temp.
1	YMX-11-11-4-4-3	02:35:47 PM	-30.20	SR	-0.0906	589	100.00	0.300	23.1
2	YMX-11-11-4-4-3	02:35:56 PM	-30.13	SR	-0.0904	589	100.00	0.300	23.1
3	YMX-11-11-4-4-3	02:36:04 PM	-30.10	SR	-0.0903	589	100.00	0.300	23.1
4	YMX-11-11-4-4-3	02:36:12 PM	-29.90	SR	-0.0897	589	100.00	0.300	23.1
5	YMX-11-11-4-4-3	02:36:20 PM	-30.00	SR	-0.0900	589	100.00	0.300	23.1

Figure S10. $[\alpha]^{25}_{D}$ of compound **1**

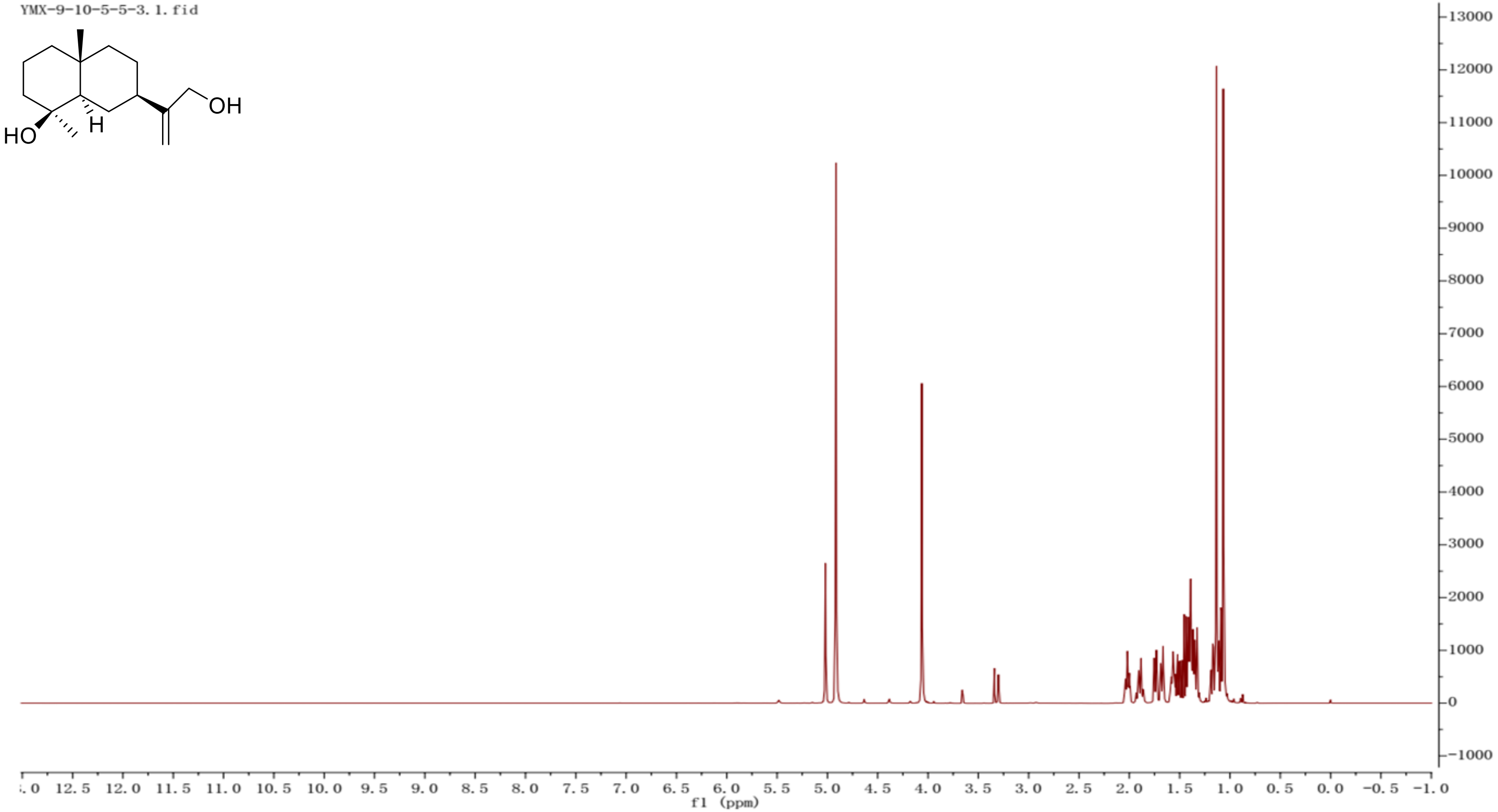

Figure S11. ^{1}H NMR spectrum of compound **2** (CD_3OD, 600 MHz)

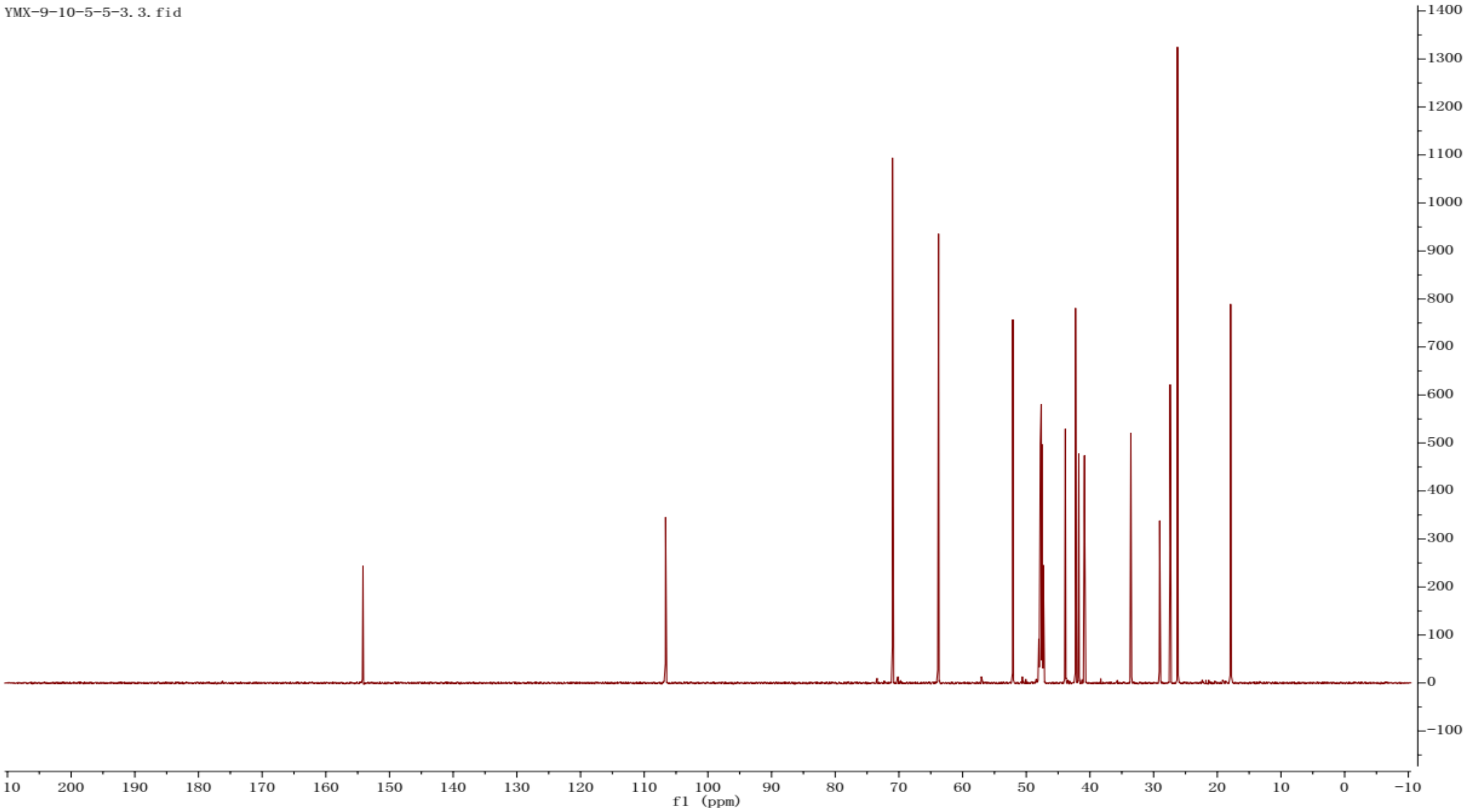

Figure S12. ^{13}C NMR spectrum of compound **2** (CD_3OD, 150 MHz)

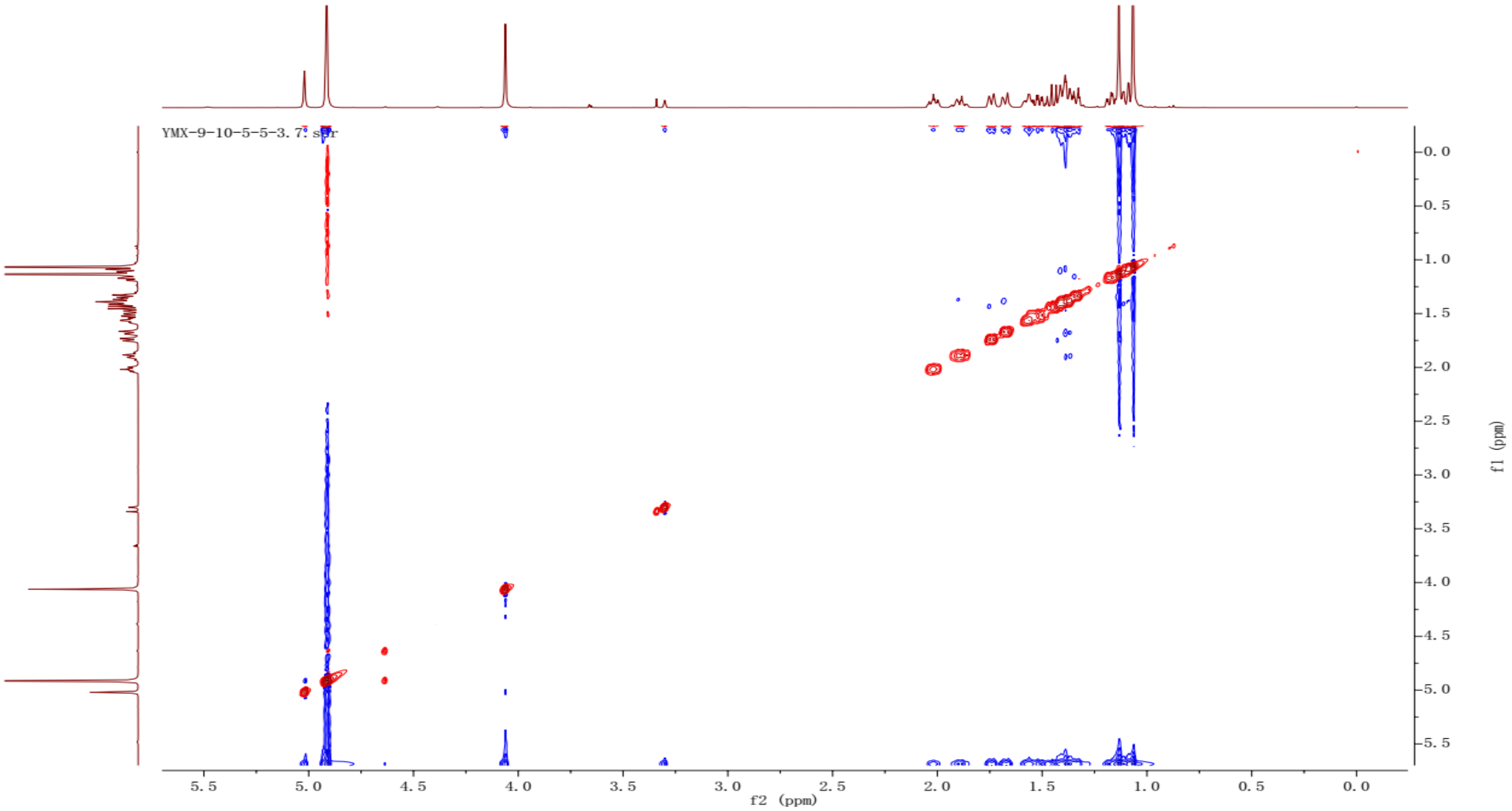

Figure S13. ROESY spectrum of compound **2** (CD_3OD, 600 MHz)

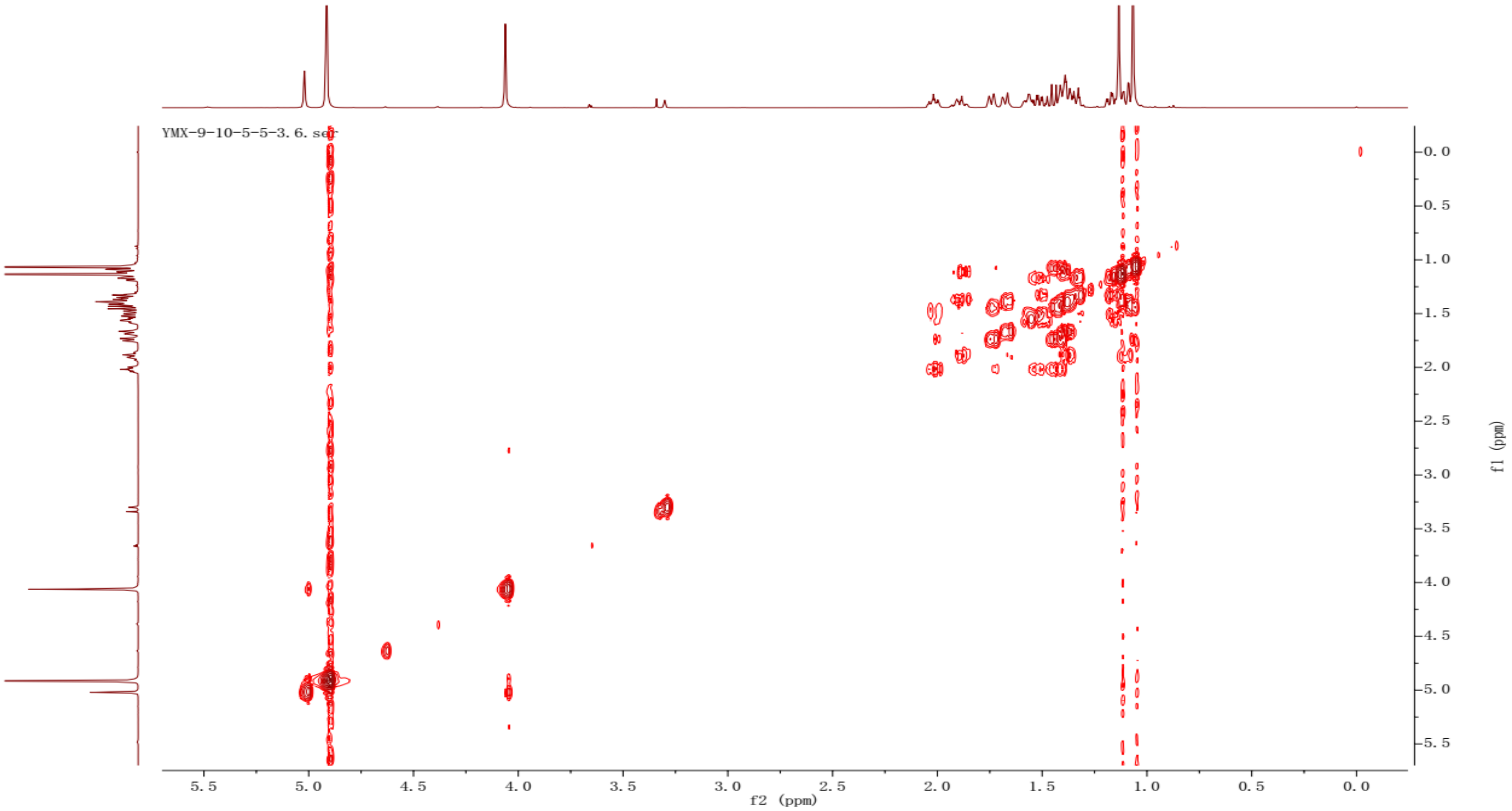

Figure S14. ^{1}H-^{1}H COSY spectrum of compound **2** (CD_3OD, 600 MHz)

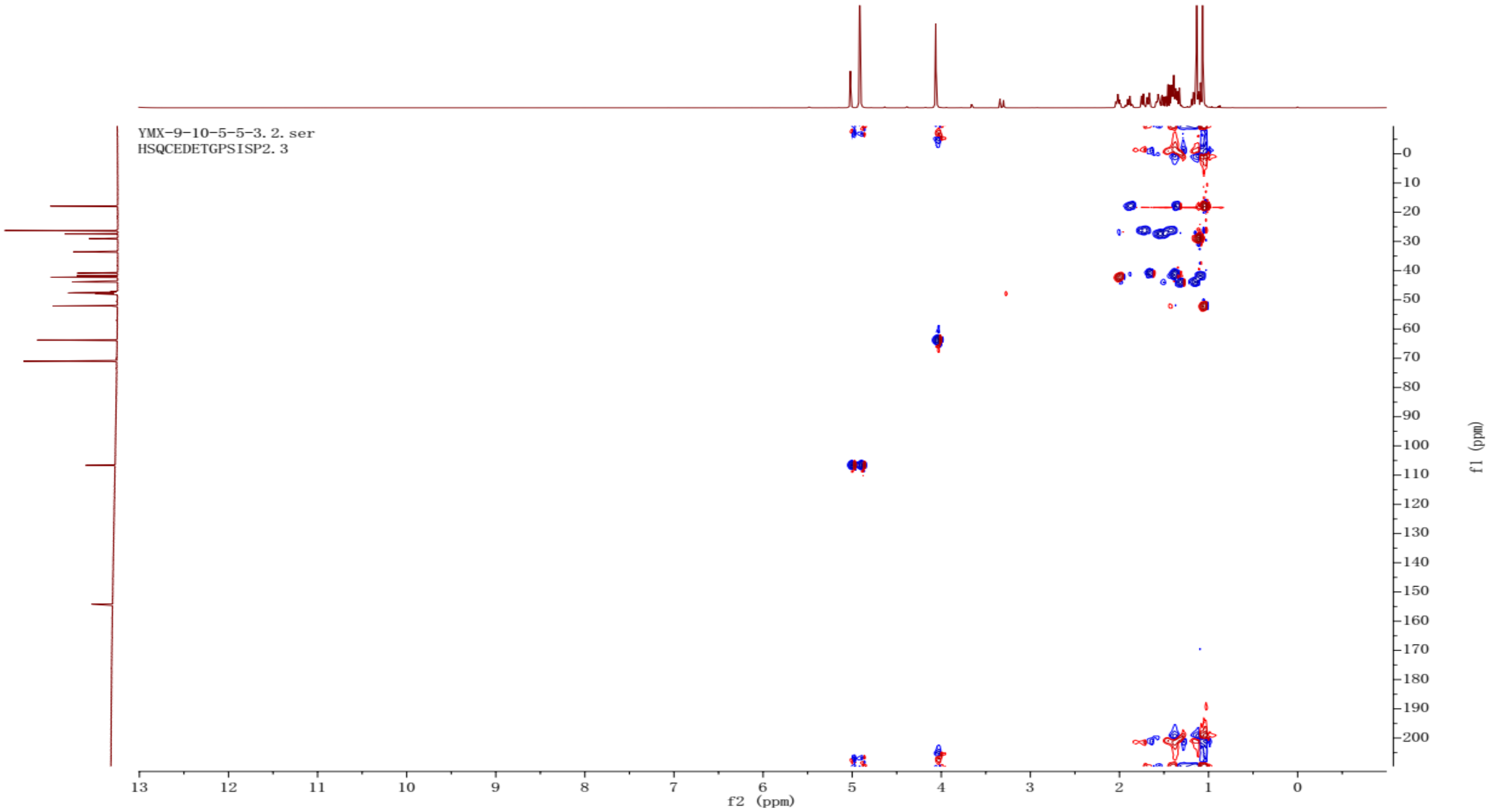

Figure S15. HSQC spectrum of compound **2** (CD_3OD, 600 MHz)

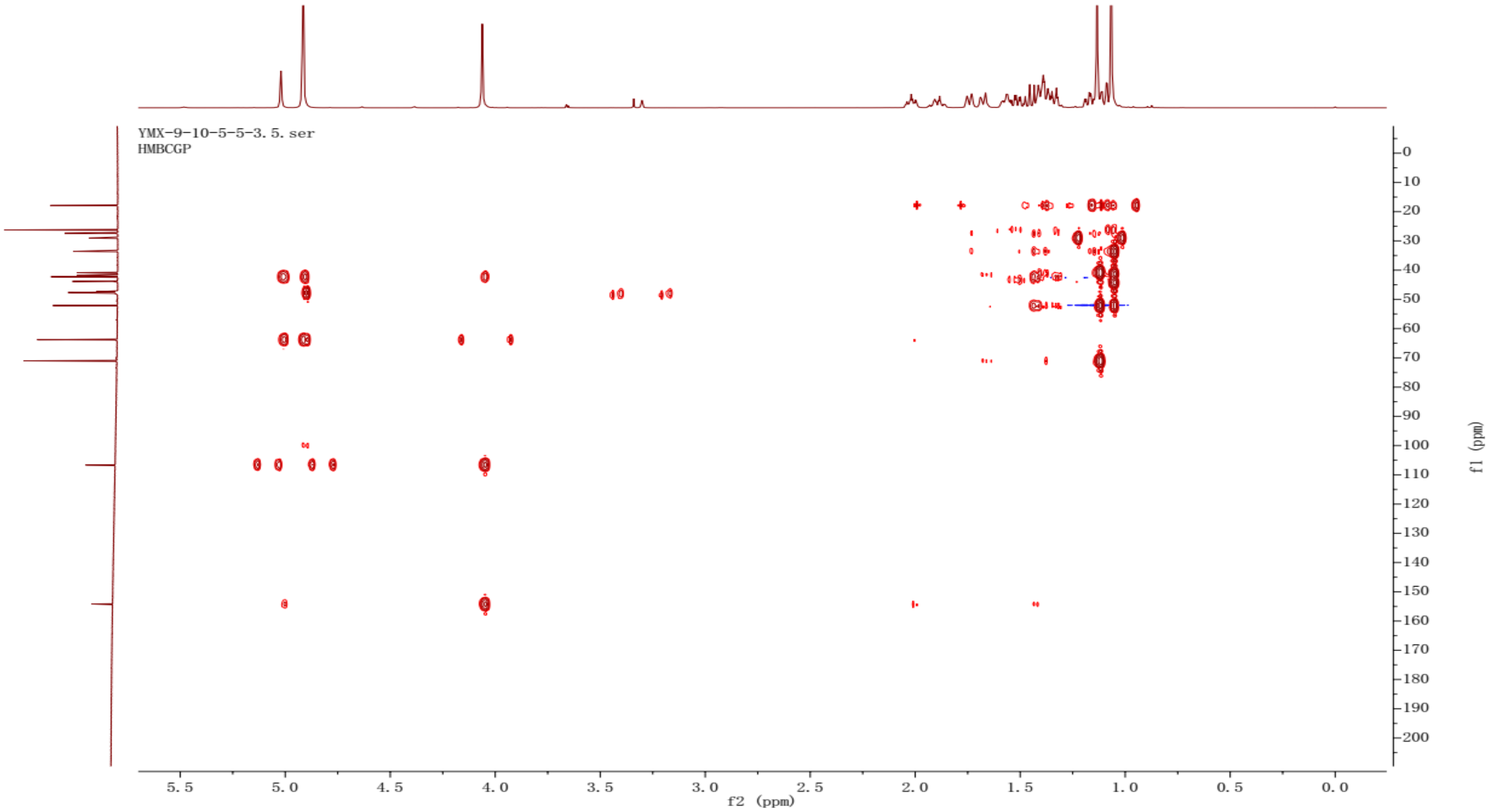

Figure S16. HMBC spectrum of compound **2** (CD_3OD, 600 MHz)

Data File: E:\DATA\2021\0901\YMX-9-10-5-5-3.lcd

Elmt	Val.	Min	Max	Elmt	Val.	Min	Max	Elmt	Val.	Min	Max	Elmt	Val.	Min	Max	Use Adduct
H	1	5	150	O	2	0	30	P	3	0	0	Se	2	0	0	H
2H	1	0	0	F	1	0	0	S	2	0	0	Br	1	0	0	Na
B	3	0	0	Na	1	0	0	Cl	1	0	0	Pd	2	0	0	
C	4	5	100	Mg	2	0	0	Co	2	0	0	Ag	1	0	0	
N	3	0	10	Si	4	0	0	Cu	2	0	0	I	3	0	0	

Error Margin (ppm):	5	DBE Range:	not fixed	Electron Ions:	both
HC Ratio:	unlimited	Apply N Rule:	yes	Use MSn Info:	yes
Max Isotopes:	all	Isotope RI (%):	1.00	Isotope Res:	10000
MSn Iso RI (%):	75.00	MSn Logic Mode:	OR	Max Results:	20

Event#: 1 MS(E+) Ret. Time : 0.280 -> 1.000 Scan# : 43 -> 151

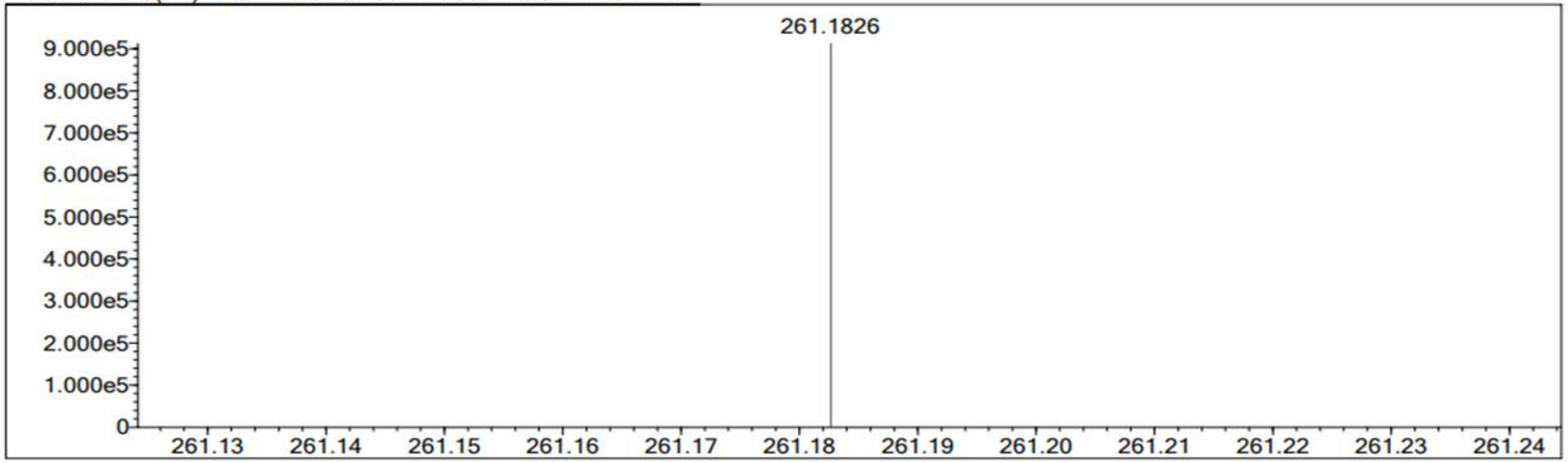

Figure S17. HRESI spectrum of compound **2**

数据集: YMX-9-10-5-5-3 - RawData

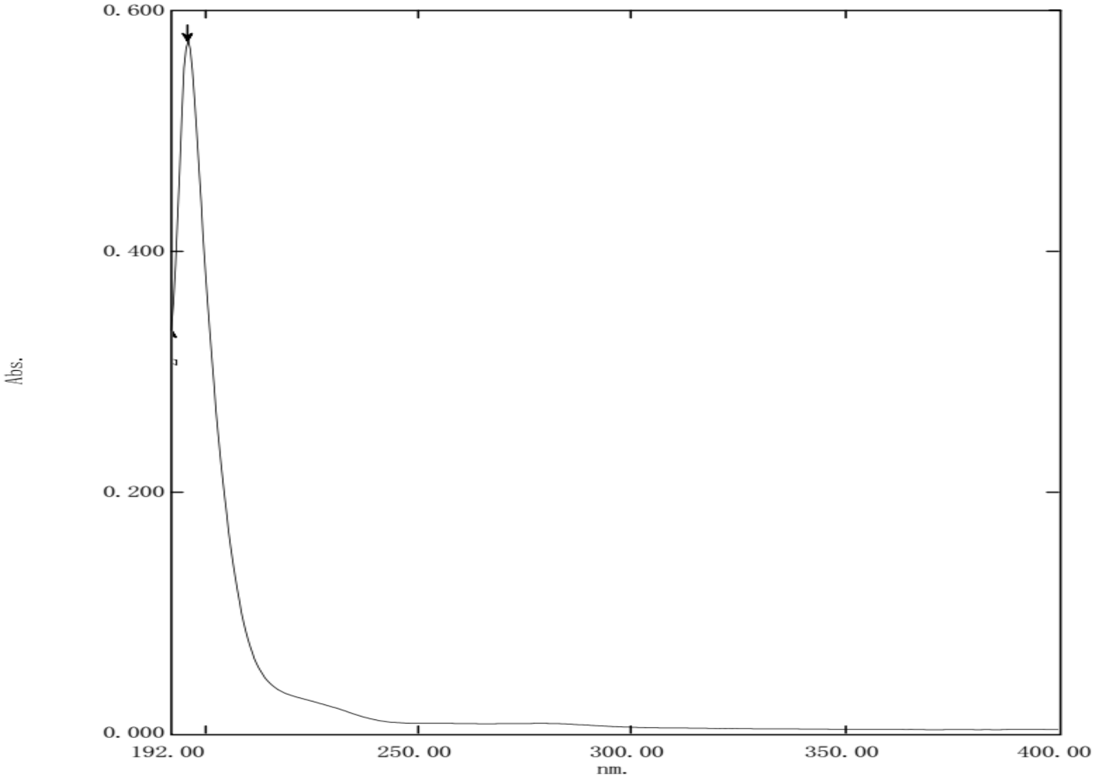

[测定属性]
波长范围(nm): 190. 00 到 600. 00
扫描速度: 中速
采样间隔: 0. 5
自动采样间隔: 停用
扫描模式: 单个

[仪器属性]
仪器类型: UV-2700 系列
测定方式: 吸收值
狭缝宽: 5. 0 nm
积分时间: 0. 1 秒
光源转换波长: 323. 0 nm
检测器单元: 直接
S/R 转换: 标准
阶梯校正: OFF

[附件属性]
附件: 无

[数据处理参数]
阈值: 0. 0010000
点: 4
内插: 停用
平均: 停用

[样品准备属性]
重量:
体积:
稀释:
光程长: 10mm
附加信息: 样品浓度: 0. 0300毫克/毫升
溶剂: 甲醇

No.	P/V	波长(nm)	Abs.	描述
1	↑	196. 00	0. 575	
2	↓	192. 00	0. 334	

Figure S18. CD and UV spectrum of compound **2**

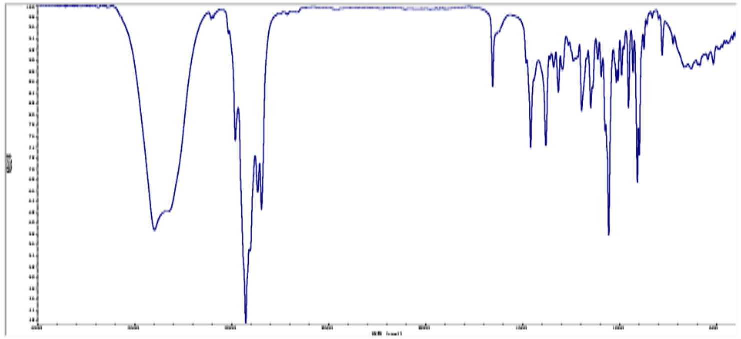

Sample Name: YMX-9-10-5-5-3
KBr压片
采集时间: 星期四 11月 19 10:11:27 2020 (GMT+08:00)
仪器型号: NICOLET iS10
Software version: OMNIC 9.8.372

样品扫描次数: 16
背景扫描次数: 16
分辨率: 4.000
采样增益: 1.0
动镜速度: 0.4747
光阑: 80.00

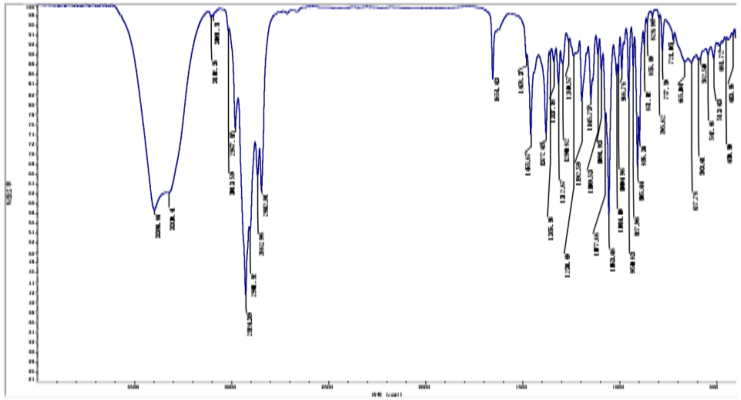

Sample Name: YMX-9-10-5-5-3
KBr压片
采集时间: 星期四 11月 19 10:11:27 2020 (GMT+08:00)
仪器型号: NICOLET iS10
Software version: OMNIC 9.8.372

样品扫描次数: 16
背景扫描次数: 16
分辨率: 4.000
采样增益: 1.0
动镜速度: 0.4747
光阑: 80.00

星期四 11月 19 10:21:41 2020 (GMT+08:00)
标峰:
谱图: YMX-9-10-5-5-3
范围: 4000.00 400.00
（绝对）阈值: 98.492
灵敏度: 99
峰表:

峰位置:	402.81	强度:	94.327
峰位置:	413.95	强度:	93.904
峰位置:	438.18	强度:	93.018
峰位置:	450.90	强度:	93.137
峰位置:	467.24	强度:	92.399
峰位置:	481.72	强度:	91.910
峰位置:	513.43	强度:	89.238
峰位置:	542.68	强度:	89.996
峰位置:	582.50	强度:	88.953
峰位置:	593.41	强度:	89.100
峰位置:	627.78	强度:	88.386
峰位置:	665.04	强度:	88.640
峰位置:	721.05	强度:	92.924
峰位置:	777.59	强度:	90.905
峰位置:	795.62	强度:	97.539
峰位置:	828.98	强度:	97.784
峰位置:	855.89	强度:	96.464
峰位置:	871.02	强度:	92.053
峰位置:	895.34	强度:	72.446
峰位置:	905.44	强度:	67.441
峰位置:	927.98	强度:	87.806
峰位置:	950.83	强度:	81.284
峰位置:	974.22	强度:	92.030
峰位置:	986.76	强度:	87.135
峰位置:	1004.96	强度:	86.176
峰位置:	1014.09	强度:	85.855
峰位置:	1053.46	强度:	57.686
峰位置:	1071.52	强度:	76.920
峰位置:	1091.93	强度:	86.914
峰位置:	1109.53	强度:	90.065
峰位置:	1145.73	强度:	81.207
峰位置:	1192.59	强度:	80.634
峰位置:	1236.25	强度:	89.819
峰位置:	1260.57	强度:	92.719
峰位置:	1290.82	强度:	88.204
峰位置:	1312.67	强度:	84.129
峰位置:	1337.18	强度:	88.586
峰位置:	1355.98	强度:	89.997
峰位置:	1377.45	强度:	74.205
峰位置:	1455.67	强度:	73.854
峰位置:	1478.37	强度:	89.459
峰位置:	1651.43	强度:	85.112
峰位置:	2710.46	强度:	98.337
峰位置:	2842.91	强度:	62.505
峰位置:	2862.98	强度:	65.579
峰位置:	2901.92	强度:	54.765
峰位置:	2924.39	强度:	41.457
峰位置:	2977.92	强度:	75.158
峰位置:	3013.59	强度:	94.910
峰位置:	3091.51	强度:	97.722
峰位置:	3102.35	强度:	97.548
峰位置:	3394.64	强度:	58.607

Figure S19. IR spectrum of compound **2**

Rudolph Research Analytical

This sample was measured on an Autopol VI, Serial #91058
Manufactured by Rudolph Research Analytical, Hackettstown, NJ, USA.

Measurement Date : Thursday, 26-NOV-2020

Set Temperature : OFF

Time Delay : Disabled

Delay between Measurement : Disabled

n	Average	Std.Dev.	% RSD	Maximum	Minimum
5	-12.20	0.31	-2.54	-11.87	-12.67

S.No	Sample ID	Time	Result	Scale	OR °Arc	WLG.nm	Lg.mm	Conc.g/100ml	Temp.
1	YMX-9-10-5-5-3	03:30:04 PM	-12.67	SR	-0.0190	589	100.00	0.150	23.2
2	YMX-9-10-5-5-3	03:30:13 PM	-12.33	SR	-0.0185	589	100.00	0.150	23.2
3	YMX-9-10-5-5-3	03:30:21 PM	-12.07	SR	-0.0181	589	100.00	0.150	23.2
4	YMX-9-10-5-5-3	03:30:29 PM	-11.87	SR	-0.0178	589	100.00	0.150	23.2
5	YMX-9-10-5-5-3	03:30:37 PM	-12.07	SR	-0.0181	589	100.00	0.150	23.2

Figure S20. $[\alpha]^{25}_{D}$ of compound **2**

Crystal data for xm7: $C_{15}H_{26}O_2$, M = 238.36, a = 12.1080(3) Å, b = 6.30390(10) Å, c = 18.5396(4) Å, α = 90°, β = 94.6690(10)°, γ = 90°, V = 1410.39(5) Å^3, T = 100.(2) K, space group P1211, Z = 4, μ(Cu Kα) = 0.560 mm^{-1}, 30099 reflections measured, 5508 independent reflections (R_{int} = 0.0467). The final R_1 values were 0.0312 ($I > 2\sigma(I)$). The final $wR(F^2)$ values were 0.0785 ($I > 2\sigma(I)$). The final R_1 values were 0.0320 (all data). The final $wR(F^2)$ values were 0.0792 (all data). The goodness of fit on F^2 was 1.078. Flack parameter = 0.03(5).

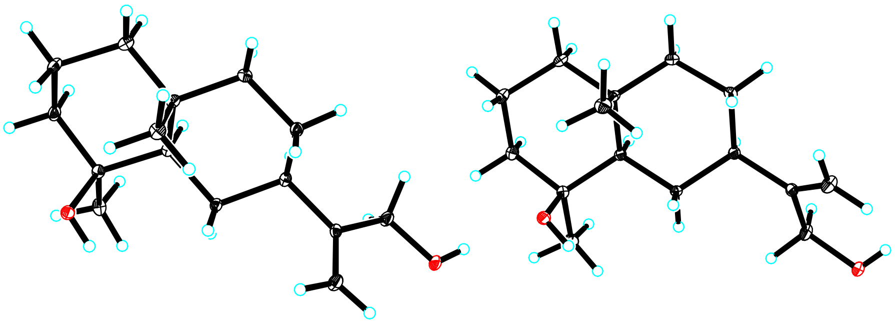

View of the molecules in an asymmetric unit.

Displacement ellipsoids are drawn at the 30% probability level.

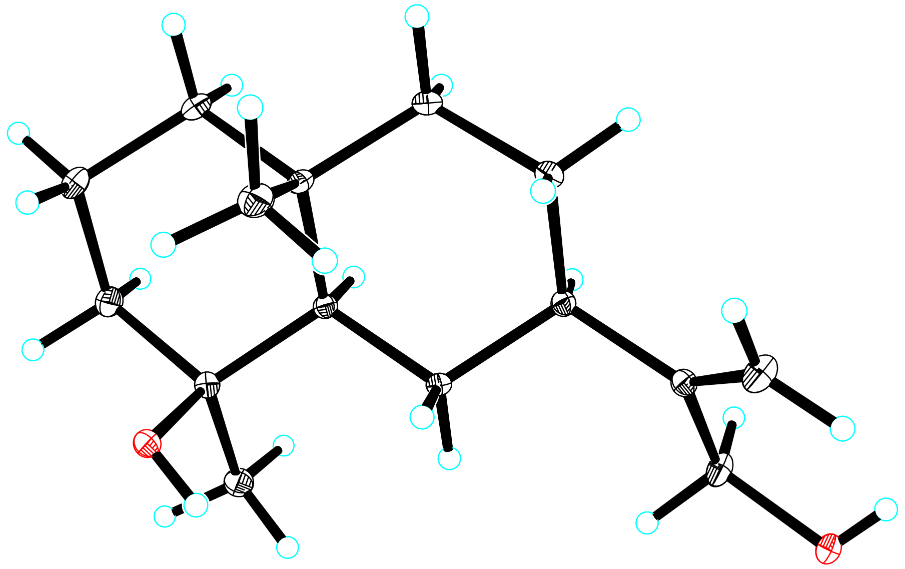

View of a molecule of xm7 with the atom-labelling scheme.

Displacement ellipsoids are drawn at the 30% probability level.

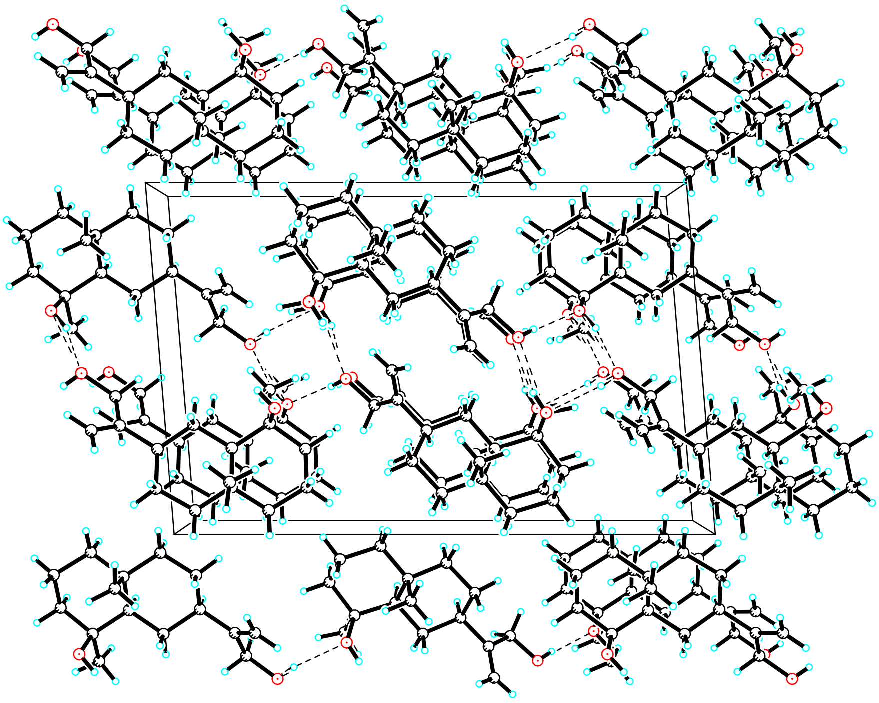

View of the pack drawing of xm7.

Hydrogen-bonds are shown as dashed lines.

Table 1. Crystal data and structure refinement for xm7_0m.

Identification code	global	
Empirical formula	C15 H26 O2	
Formula weight	238.36	
Temperature	100(2) K	
Wavelength	1.54178 Å	
Crystal system	Monoclinic	
Space group	P 1 21 1	
Unit cell dimensions	a = 12.1080(3) Å	= 90°.
	b = 6.30390(10) Å	= 94.6690(10)°.
	c = 18.5396(4) Å	= 90°.
Volume	1410.39(5) Å^3	
Z	4	
Density (calculated)	1.123 Mg/m^3	
Absorption coefficient	0.560 mm^{-1}	

F(000)	528
Crystal size	0.580 x 0.200 x 0.100 mm^3
Theta range for data collection	2.39 to 72.47°.
Index ranges	-14<=h<=14, -7<=k<=7, -22<=l<=22
Reflections collected	30099
Independent reflections	5508 [R(int) = 0.0467]
Completeness to theta = 72.47°	99.8 %
Absorption correction	Semi-empirical from equivalents
Max. and min. transmission	0.95 and 0.76
Refinement method	Full-matrix least-squares on F^2
Data / restraints / parameters	5508 / 1 / 318
Goodness-of-fit on F^2	1.078
Final R indices [I>2sigma(I)]	R1 = 0.0312, wR2 = 0.0785
R indices (all data)	R1 = 0.0320, wR2 = 0.0792
Absolute structure parameter	0.03(5)
Largest diff. peak and hole	0.192 and -0.280 e.$Å^{-3}$

Figure S21. X-ray crystallographic analysis for compound **2**

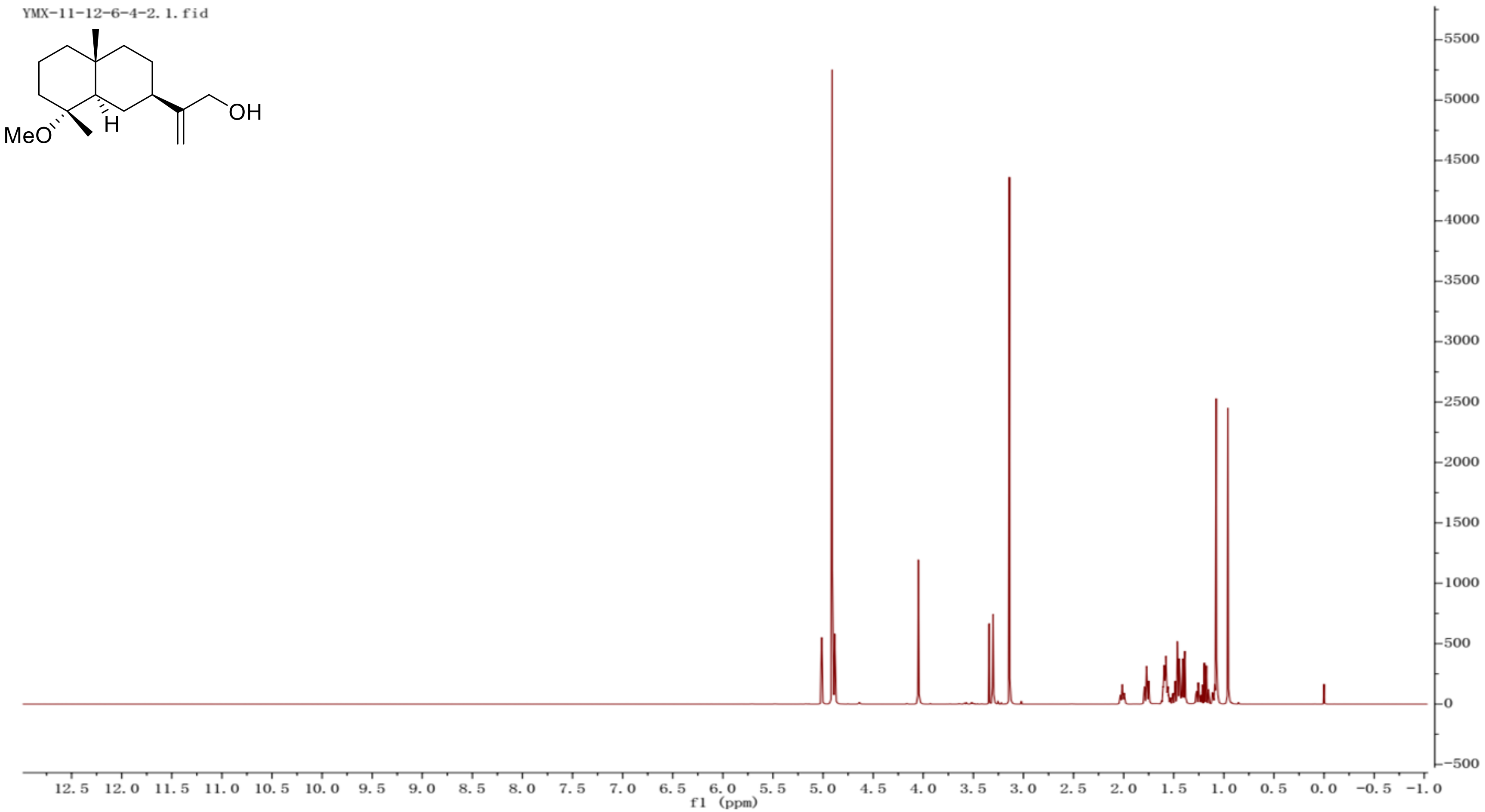

Figure S22. ^{1}H NMR spectrum of compound **3** (CD_3OD, 600 MHz)

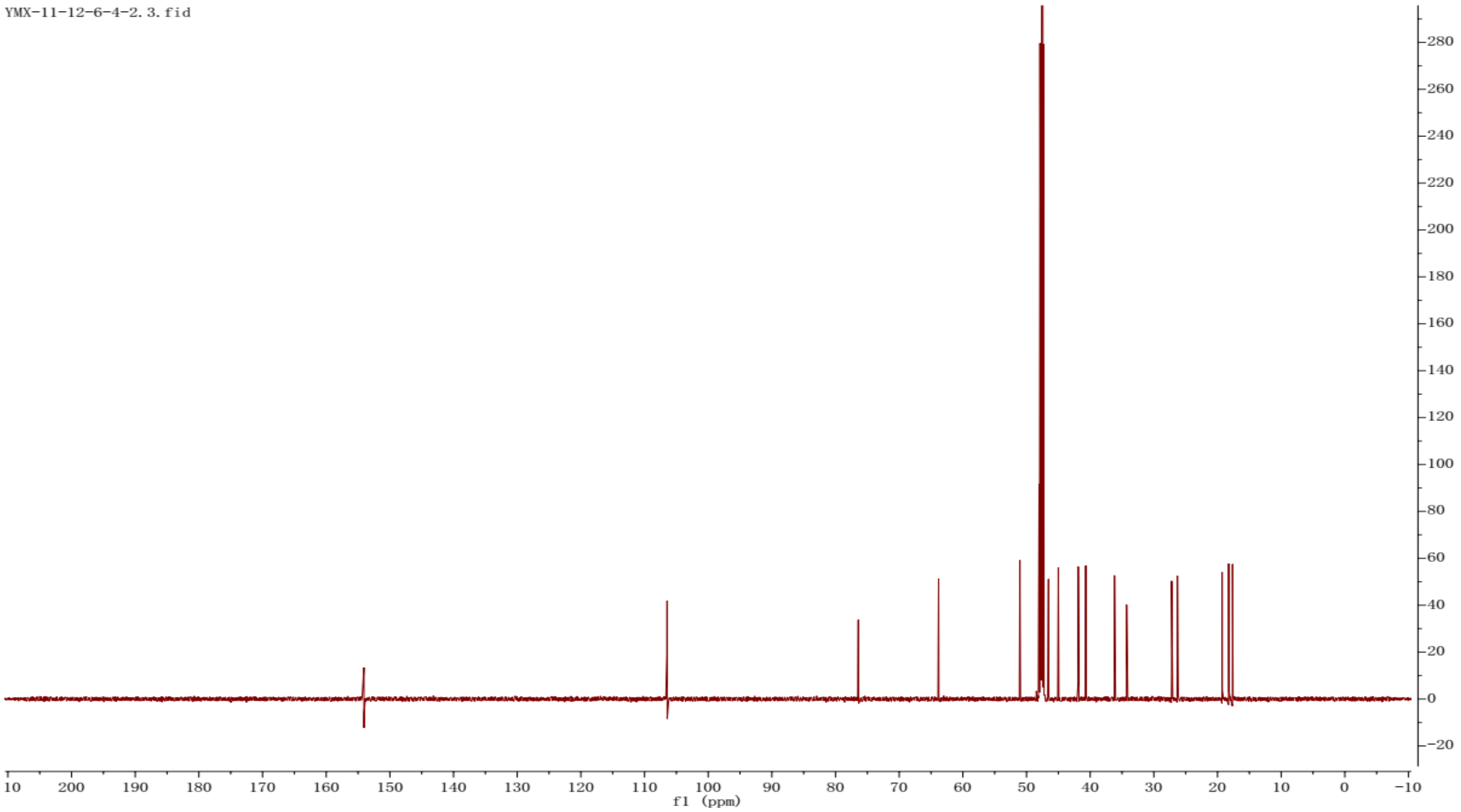

Figure S23. ^{13}C NMR spectrum of compound **3** (CD_3OD, 150 MHz)

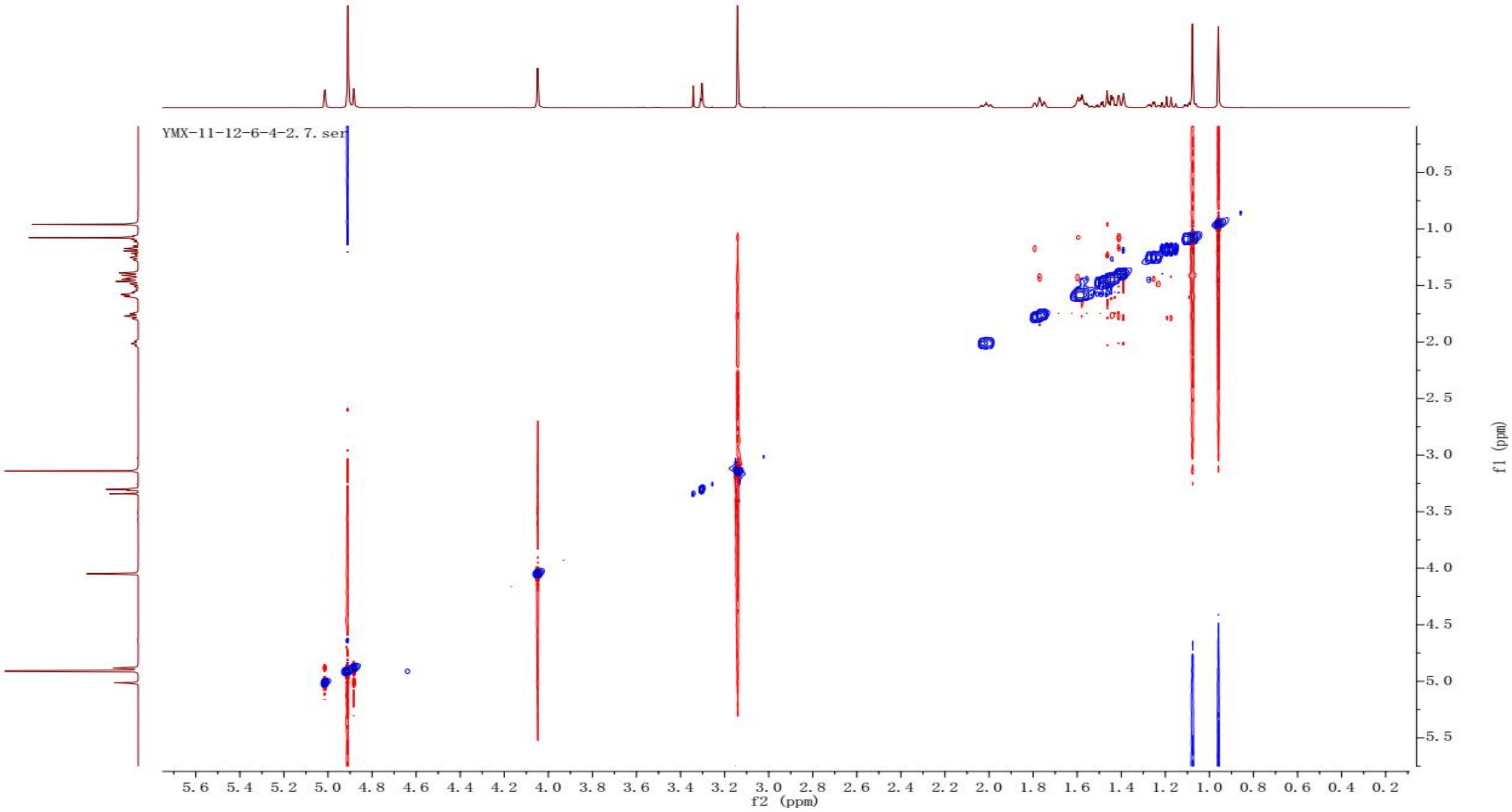

Figure S24. ROESY spectrum of compound **3** (CD_3OD, 600 MHz)

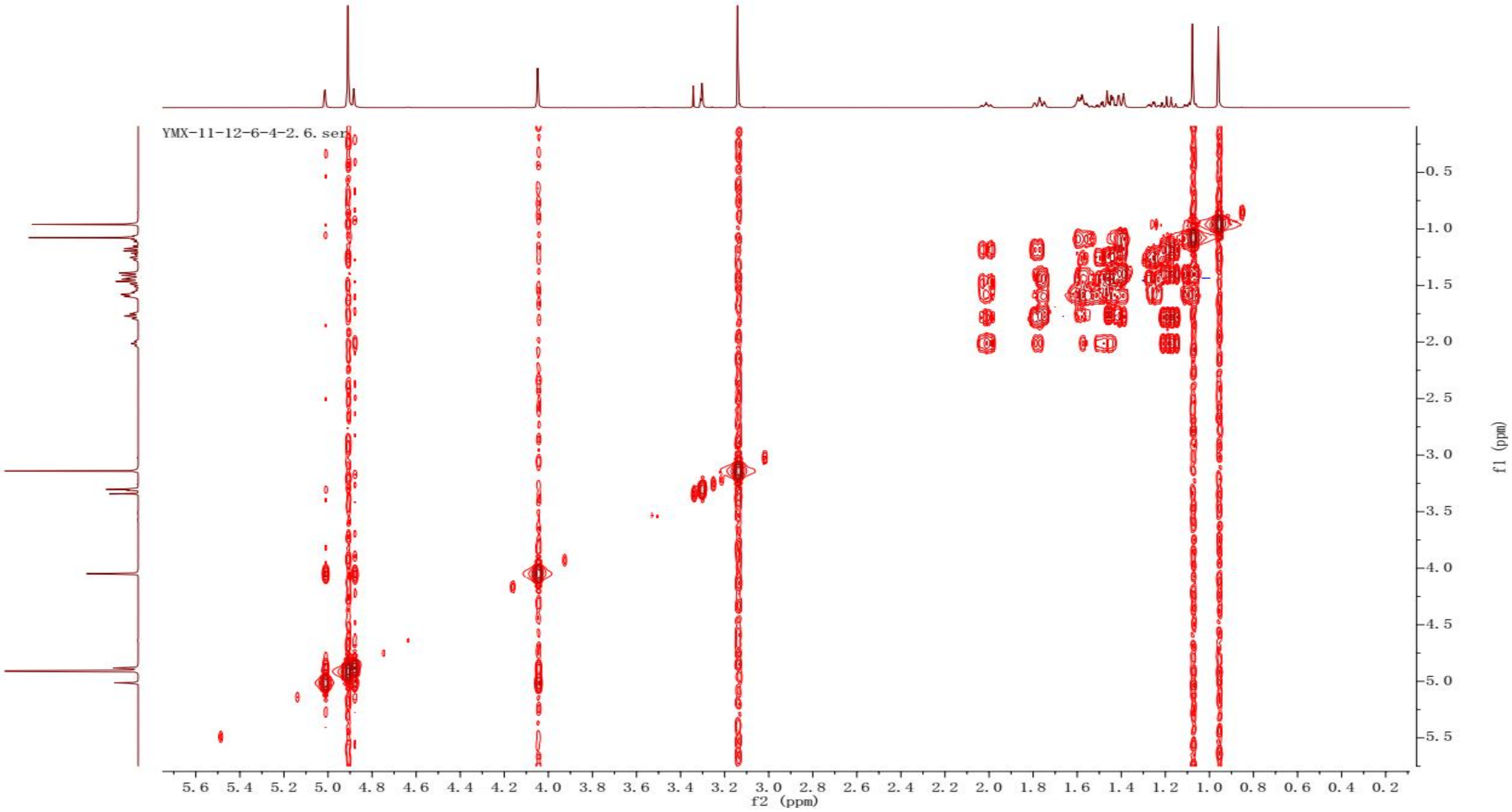

Figure S25. ^{1}H-^{1}H COSY spectrum of compound **3** (CD_3OD, 600 MHz)

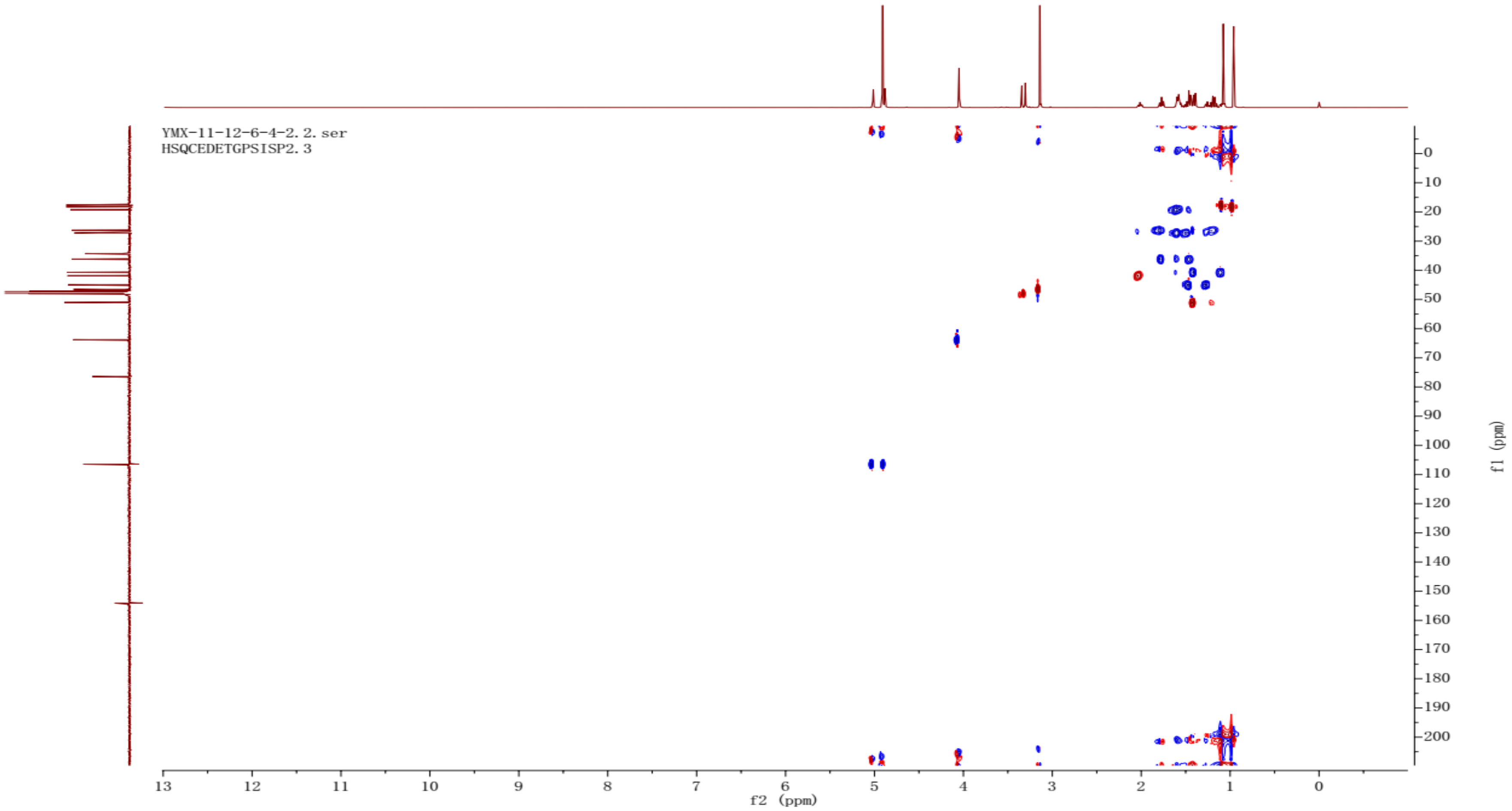

Figure S26. HSQC spectrum of compound **3** (CD_3OD, 600 MHz)

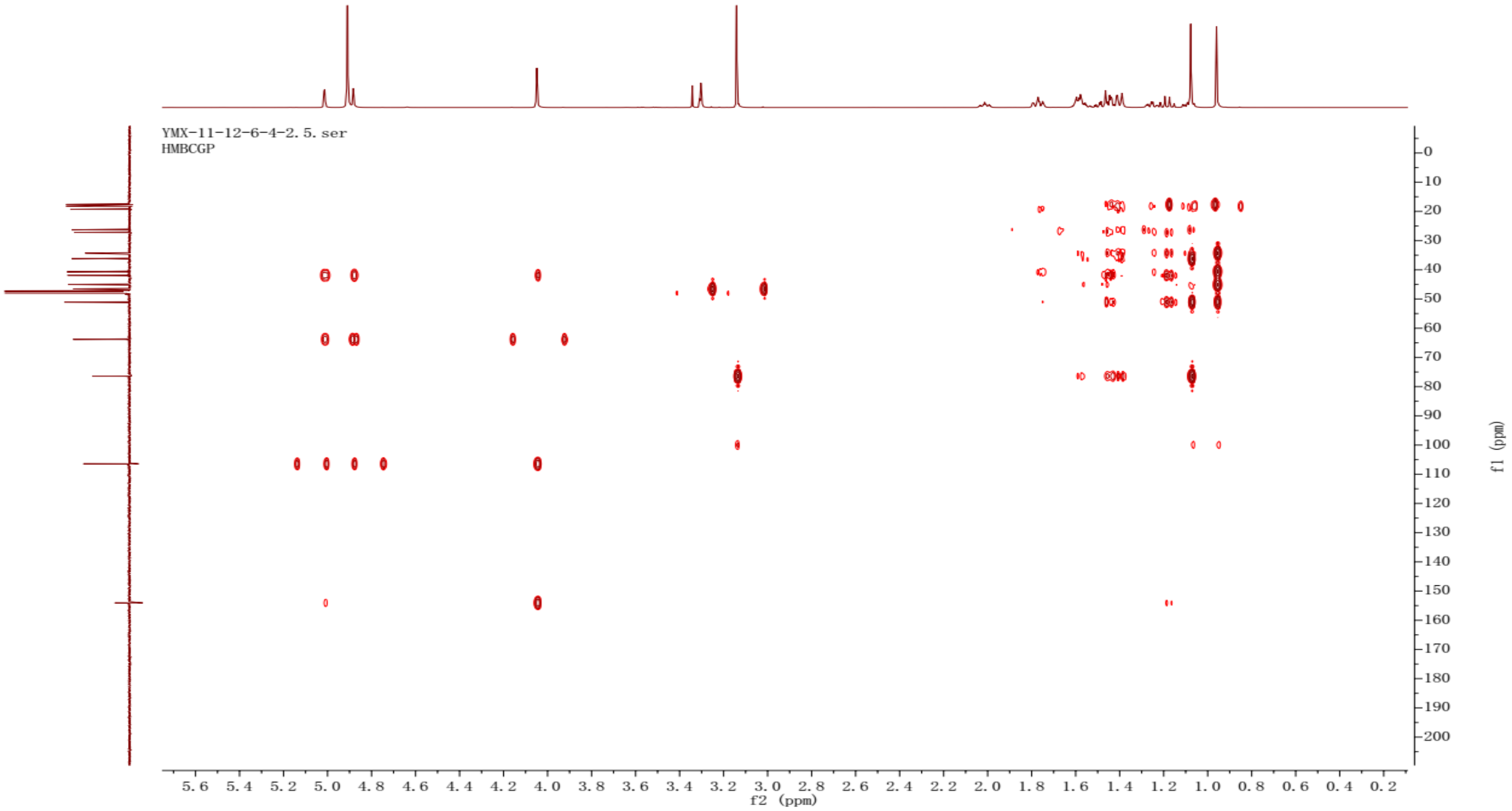

Figure S27. HMBC spectrum of compound **3** (CD_3OD, 600 MHz)

Qualitative Analysis Report

**Data Filename**	YMX-11-12-6-4-2.d	**Sample Name**	YMX-11-12-6-4-2
**Sample Type**	Sample	**Position**	p1b6
**Instrument Name**	Instrument 1	**User Name**	Q-TOF-HP\Q-TOF
**Acq Method**	default20190111-ms.m	**Acquired Time**	1/12/2020 3:53:15 PM
**IRM Calibration Status**	Success	**DA Method**	1.m
**Comment**			

**Sample Group**		**Info.**
**Acquisition SW Version**	6200 series TOF/6500 series Q-TOF B.05.01 (B5125.3)	

User Chromatograms

Fragmentor Voltage 155 **Collision Energy** 0 **Ionization Mode** ESI

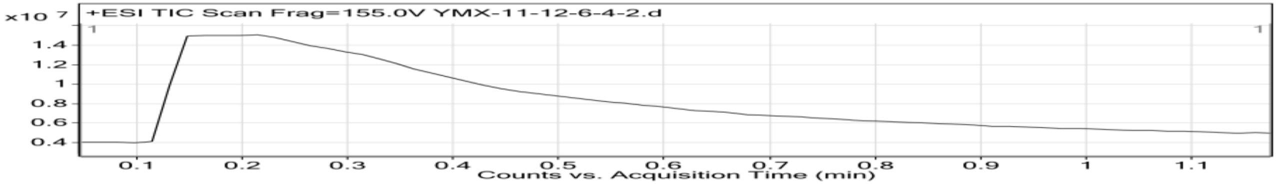

User Spectra

Fragmentor Voltage	Collision Energy	Ionization Mode
155	0	ESI

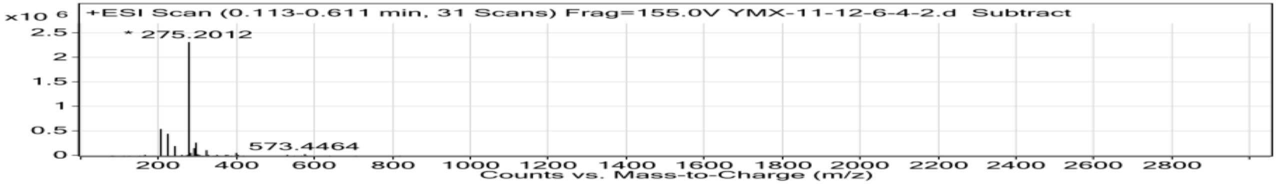

Peak List

m/z	z	Abund
203.1784	1	562934.5
221.1888	1	456599.94
238.2153	1	203270.44
275.2012		2326498.25
275.3404	1	322283.94
275.3859		111786.8
276.2007	1	1035205.25
289.1761	1	170995.88

Agilent Technologies Page 1 of 2 Printed at: 4:31 PM on: 4/15/2020

Figure S28. HRESI spectrum of compound **3**

YMX-11-12-6-4-2

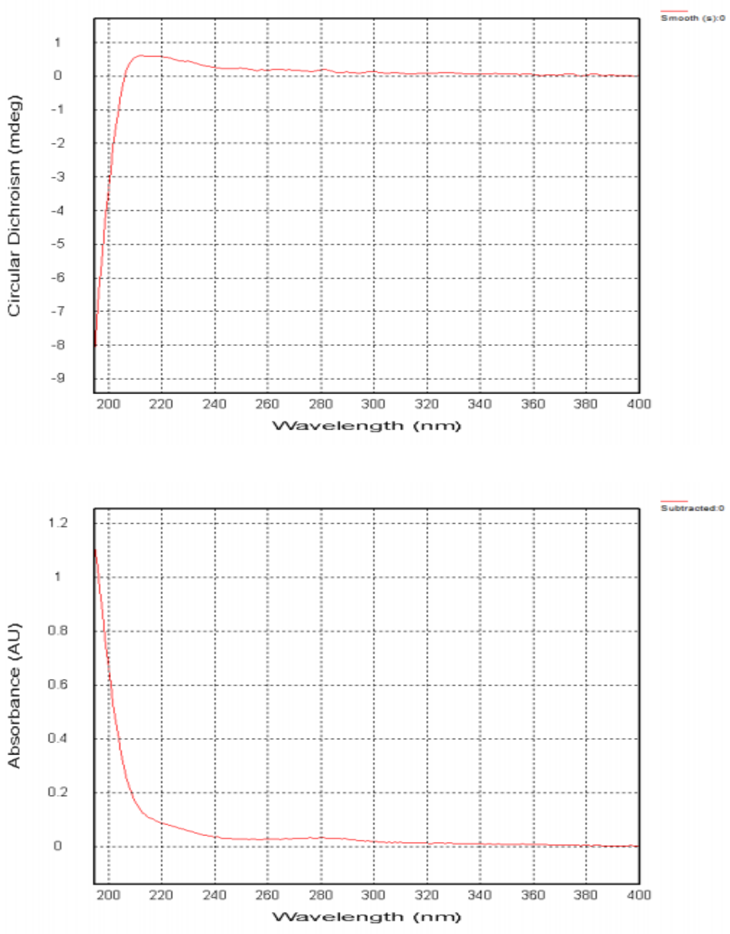

File: YMX-11-12-6-4-2 (195-400 nm) 20112716.dsx
ProBinaryX
Attributes :
- Time Stamp :Fri Nov 27 19:17:31 2020

- File ID : {D6821013-5B01-4cf8-96AB-CEAAAE56FBC2}
- Is CFR Compliant : false
- Original data has not been modified.

Remarks:
- User: CD
- Date: 2020/11/27
- Instrument: 0547
- DetectorType: LAAPD
- DichOS Calibration Correction Curve: 0547/2
- HV (CDDC channel): 0 v
- Time per point: 1 s
- Description: Sample 1
- Concentration: 0.2700mg/mL MeOH
- Pathlength: 1 mm
- Temperature: 20℃

Settings:
- Time-per-point: 1s (25us x 40000)
- SE
- Wavelength: 195nm - 400nm
- Step Size: 1nm
- Bandwidth: 1nm

Figure S29. CD and UV spectrum of compound **3**

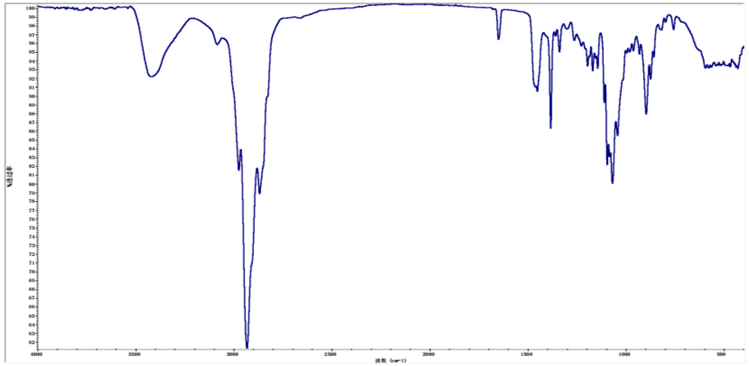

Sample Name: YMX-11-12-6-4-2
KBr压片
采集时间: 星期三 10月 28 15:29:57 2020 (GMT+08:00)
仪器型号: NICOLET iS10
Software version: OMNIC 9.8.372

样品扫描次数: 16
背景扫描次数: 16
分辨率: 4.000
采样增益: 1.0
动镜速度: 0.4747
光阑: 80.00

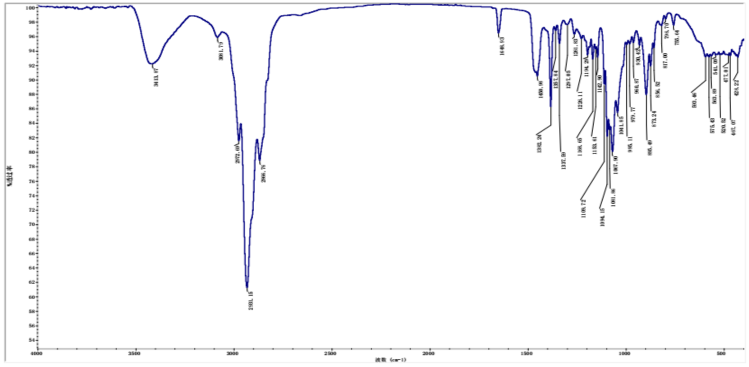

Sample Name: YMX-11-12-6-4-2
KBr压片
采集时间: 星期三 10月 28 15:29:57 2020 (GMT+08:00)
仪器型号: NICOLET iS10
Software version: OMNIC 9.8.372

样品扫描次数: 16
背景扫描次数: 16
分辨率: 4.000
采样增益: 1.0
动镜速度: 0.4747
光阑: 80.00

星期三 10月 28 16:13:13 2020 (GMT+08:00)
标峰:
谱图: YMX-11-12-6-4-2
范围: 4000.00 400.00
(绝对)阈值: 98.415
灵敏度: 99
峰表:

峰位置:	428.22	强度:	93.097
峰位置:	437.88	强度:	93.255
峰位置:	467.07	强度:	93.302
峰位置:	477.01	强度:	93.434
峰位置:	513.07	强度:	93.463
峰位置:	520.52	强度:	93.458
峰位置:	541.09	强度:	93.419
峰位置:	563.89	强度:	93.272
峰位置:	575.43	强度:	93.131
峰位置:	593.46	强度:	93.158
峰位置:	755.64	强度:	97.530
峰位置:	796.70	强度:	98.393
峰位置:	817.00	强度:	97.493
峰位置:	856.52	强度:	94.444
峰位置:	873.24	强度:	91.889
峰位置:	895.49	强度:	87.903
峰位置:	930.42	强度:	94.749
峰位置:	960.87	强度:	95.122
峰位置:	979.77	强度:	94.978
峰位置:	995.11	强度:	94.864
峰位置:	1041.85	强度:	85.458
峰位置:	1067.90	强度:	80.034
峰位置:	1081.86	强度:	83.002
峰位置:	1094.15	强度:	82.123
峰位置:	1109.72	强度:	89.205
峰位置:	1142.90	强度:	93.105
峰位置:	1153.61	强度:	94.253
峰位置:	1168.65	强度:	92.833
峰位置:	1194.29	强度:	93.392
峰位置:	1226.11	强度:	95.664
峰位置:	1261.83	强度:	96.304
峰位置:	1297.05	强度:	97.606
峰位置:	1337.59	强度:	94.985
峰位置:	1357.84	强度:	96.782
峰位置:	1382.24	强度:	86.283
峰位置:	1450.98	强度:	90.491
峰位置:	1648.93	强度:	96.434
峰位置:	2866.76	强度:	78.823
峰位置:	2931.15	强度:	61.206
峰位置:	2972.69	强度:	81.513
峰位置:	3081.79	强度:	95.841
峰位置:	3413.87	强度:	92.189

Figure S30. IR spectrum of compound **3**

Rudolph Research Analytical

This sample was measured on an Autopol VI, Serial #91058
Manufactured by Rudolph Research Analytical, Hackettstown, NJ, USA.

Measurement Date : Monday, 23-NOV-2020

Set Temperature : OFF

Time Delay : Disabled

Delay between Measurement : Disabled

n	Average	Std.Dev.	% RSD	Maximum	Minimum
5	-58.40	0.10	-0.17	-58.33	-58.53

S.No	Sample ID	Time	Result	Scale	OR °Arc	WLG.nm	Lg.mm	Conc.g/100ml	Temp.
1	YMX-11-12-6-4-2	09:21:22 PM	-58.53	SR	-0.0878	589	100.00	0.150	22.2
2	YMX-11-12-6-4-2	09:21:30 PM	-58.33	SR	-0.0875	589	100.00	0.150	22.3
3	YMX-11-12-6-4-2	09:21:38 PM	-58.33	SR	-0.0875	589	100.00	0.150	22.3
4	YMX-11-12-6-4-2	09:21:46 PM	-58.33	SR	-0.0875	589	100.00	0.150	22.3
5	YMX-11-12-6-4-2	09:21:54 PM	-58.47	SR	-0.0877	589	100.00	0.150	22.3

Figure S31. $[\alpha]^{25}_{D}$ of compound **3**

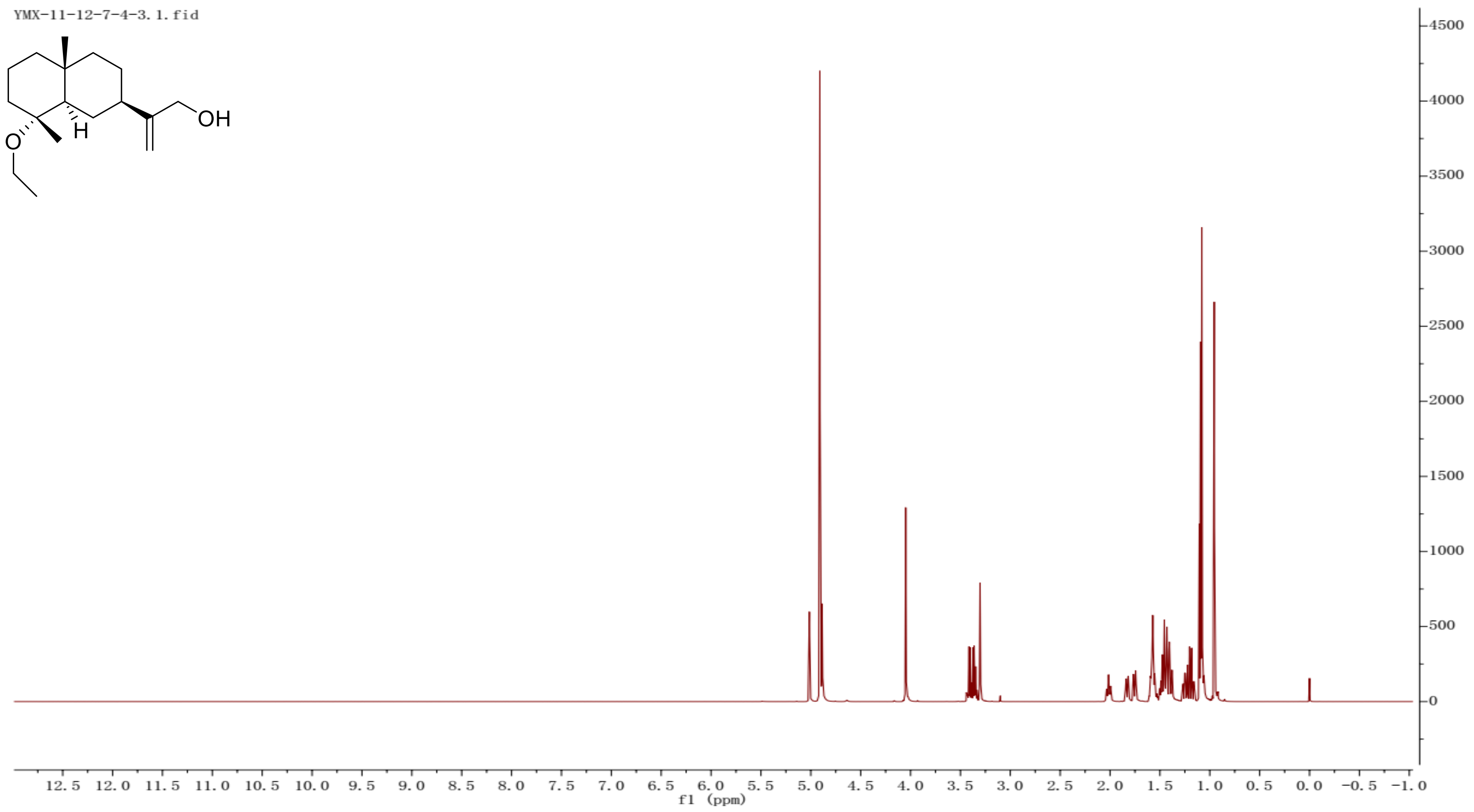

Figure S32. ^{1}H NMR spectrum of compound **4** (CD_3OD, 600 MHz)

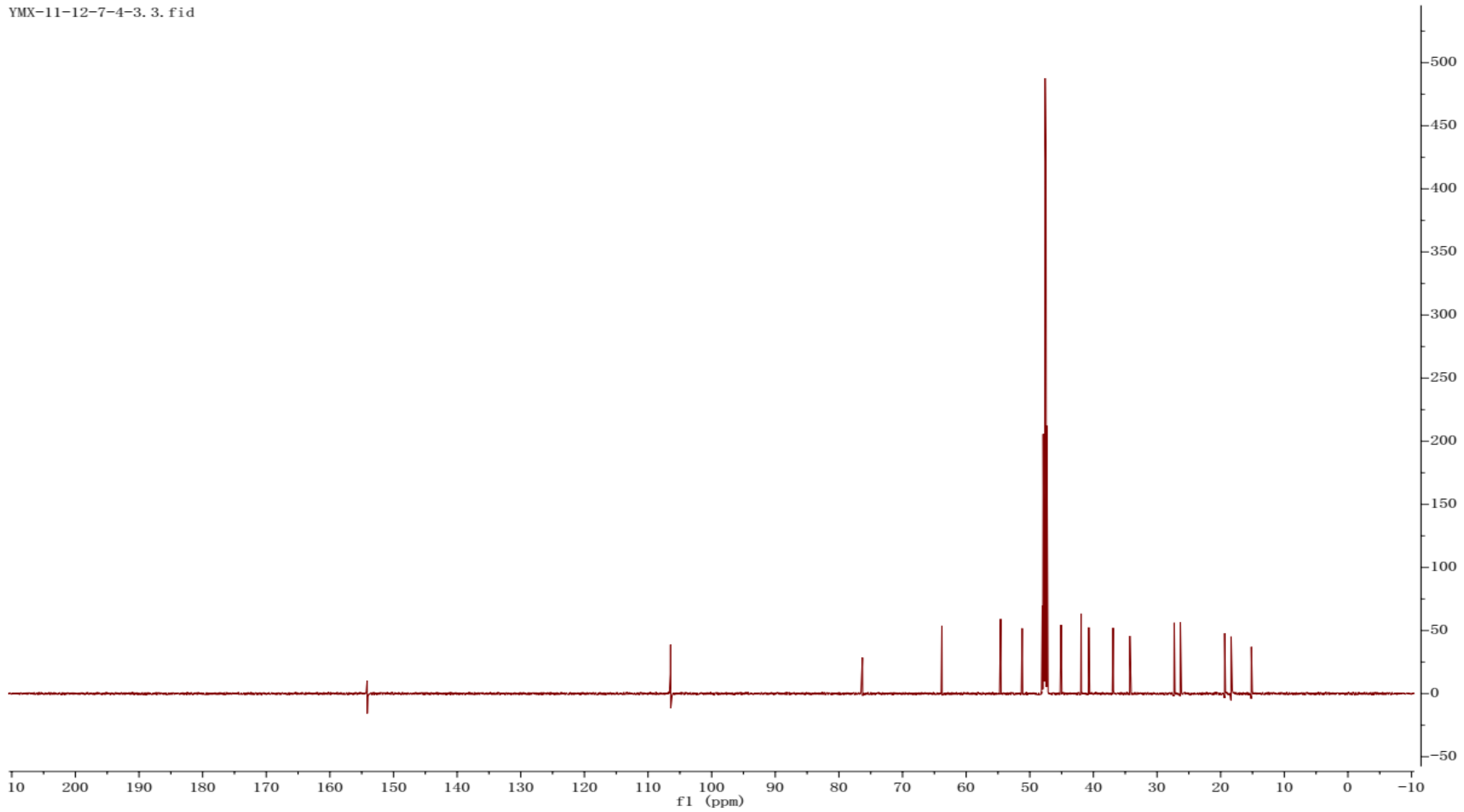

Figure S33. ^{13}C NMR spectrum of compound **4** (CD_3OD, 150 MHz)

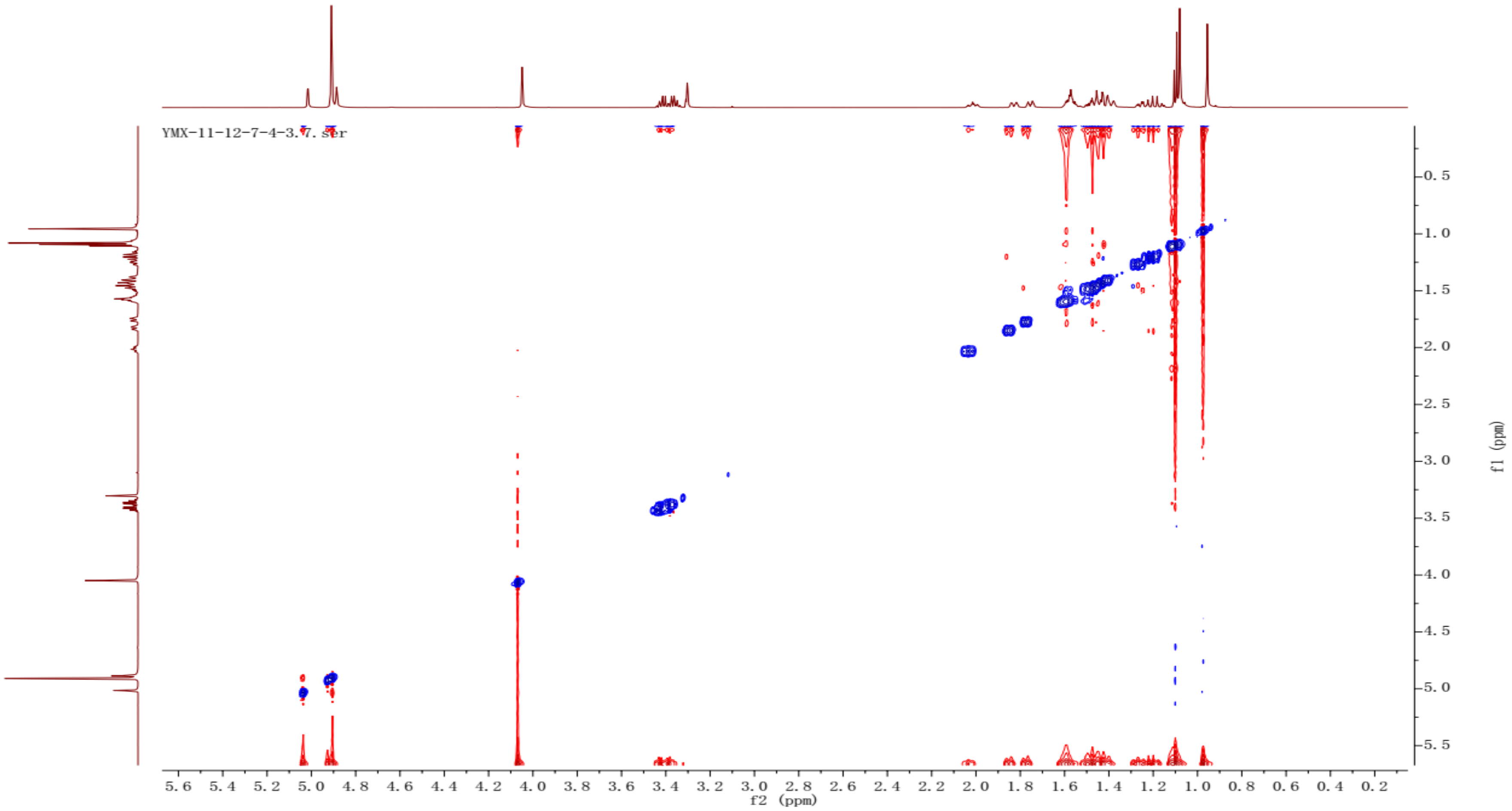

Figure S34. ROESY spectrum of compound **4** (CD_3OD, 600 MHz)

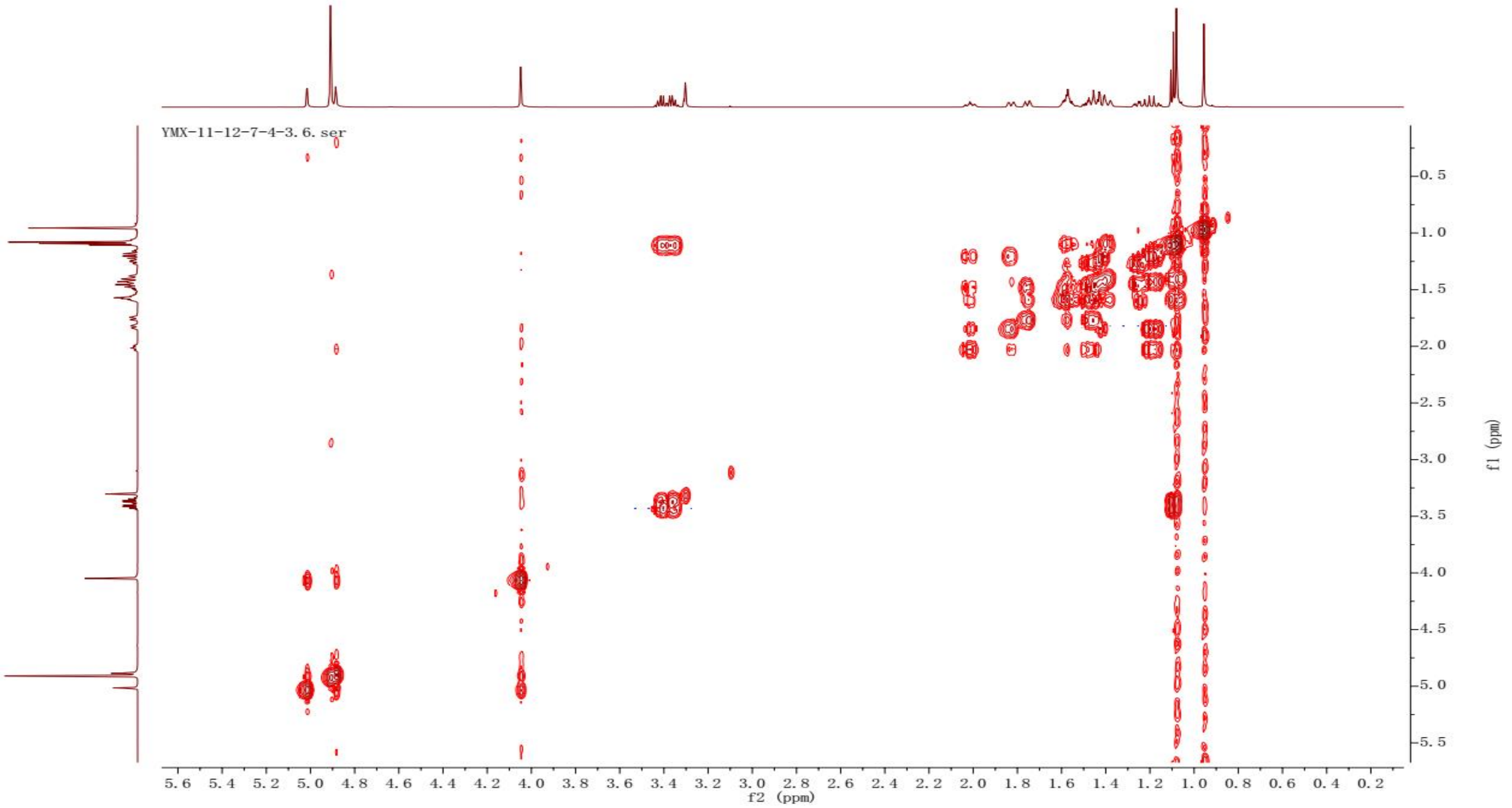

Figure S35. ^{1}H-^{1}H COSY spectrum of compound **4** (CD_3OD, 600 MHz)

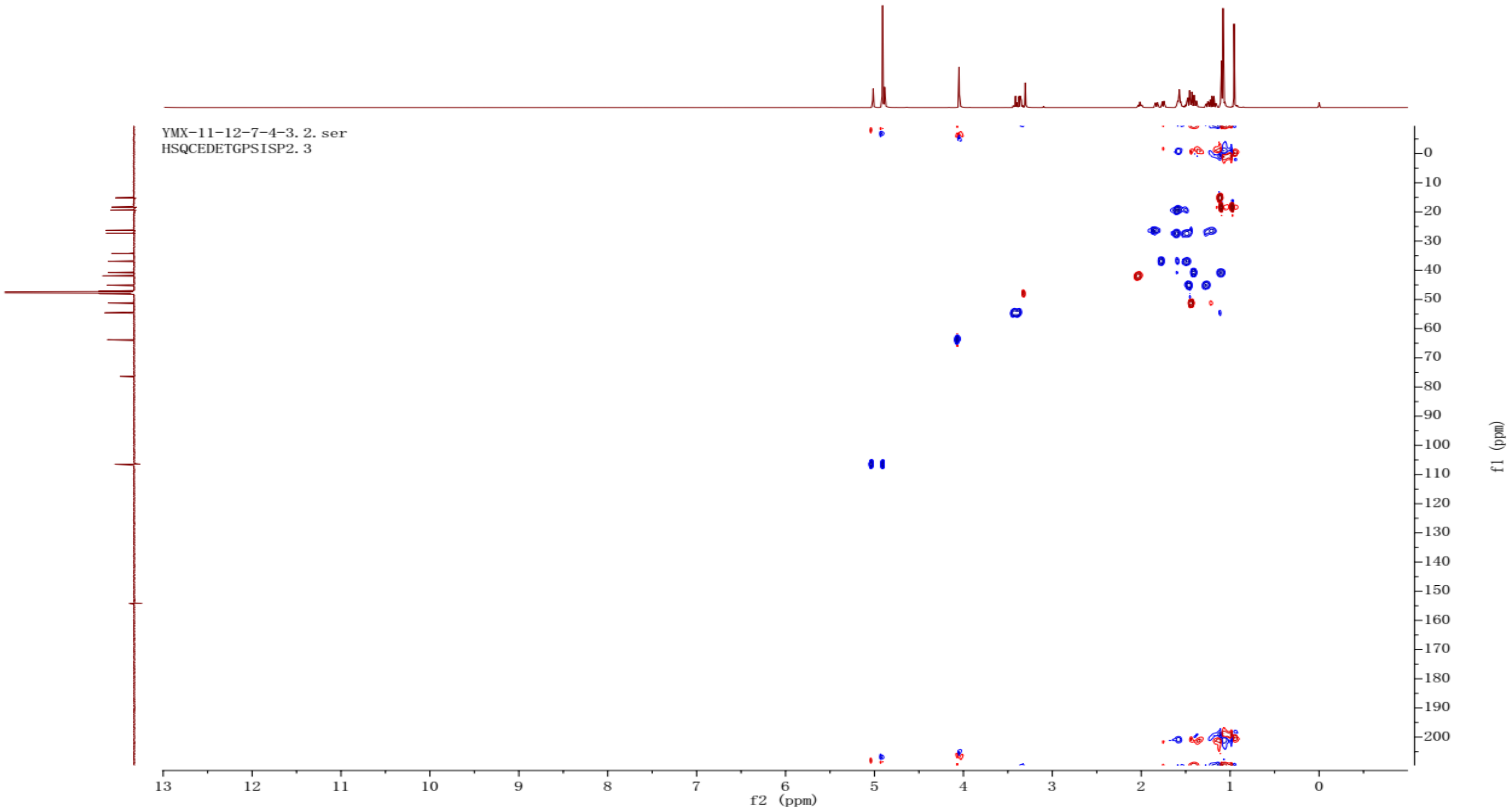

Figure S36. HSQC spectrum of compound **4** (CD_3OD, 600 MHz)

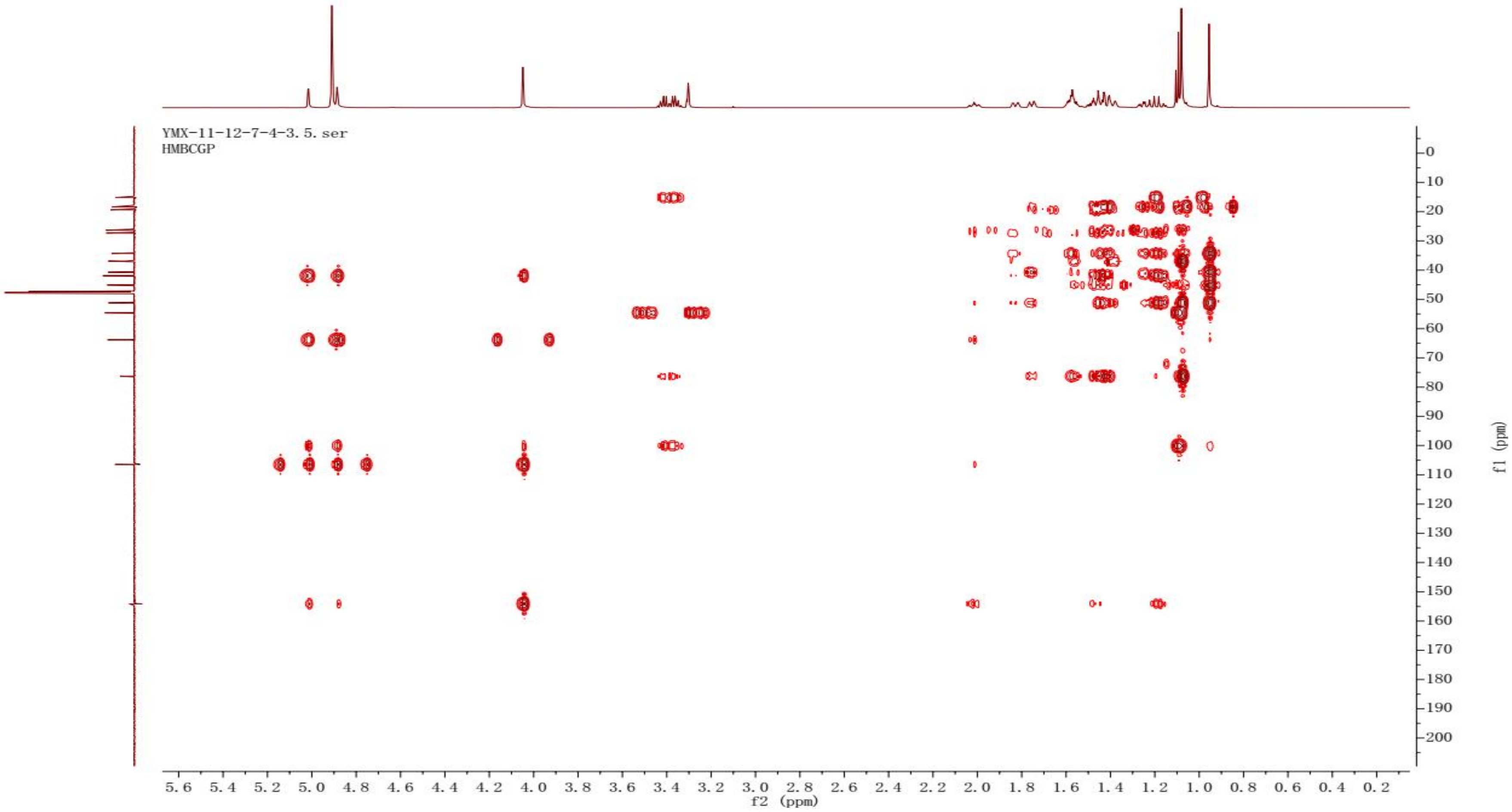

Figure S37. HMBC spectrum of compound **4** (CD_3OD, 600 MHz)

Qualitative Analysis Report

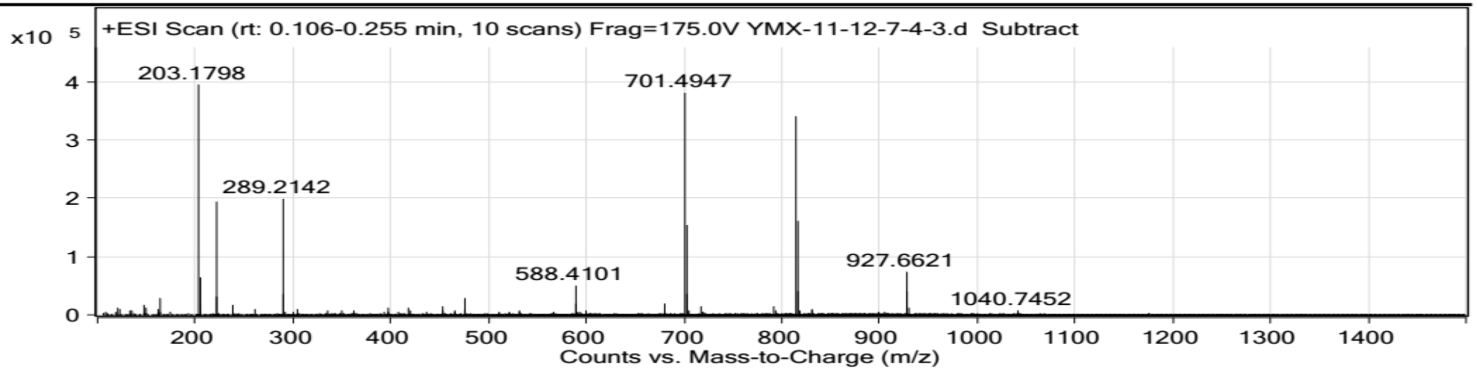

Peak List

m/z	z	Abund
163.1479	1	29645.47
203.1798	1	395609.72
204.1828	1	62958.54
205.1934		17911.16
221.1902	1	195222.67
222.1935	1	30062.8
289.2142	1	198920.55
290.2176	1	35569.34
475.3259	2	29273.89
588.4101	1	50941.28
589.413	1	17897.13
679.5111	1	18046.72
701.4947	1	380123.91
702.4978	1	153495.44
703.5	1	35624.95
814.5788	1	339993.13
815.5817	1	162160.91
816.5844	1	41294.73
927.6621	1	72421.57
928.6646	1	40550.8

Figure S38. HRESI spectrum of compound **4**

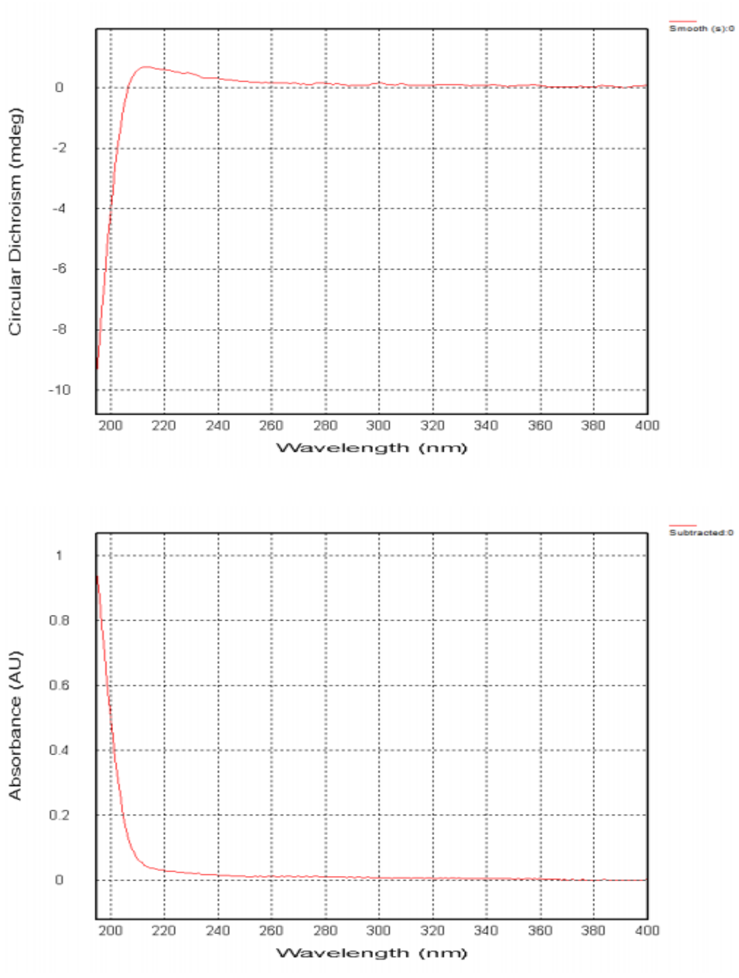

File: YMX-11-12-7-4-3 (195-400 nm) 20112719.dsx
ProBinaryX
Attributes :
- Time Stamp :Fri Nov 27 20:15:48 2020

- File ID : {E8796B06-0EF0-4e14-9505-25E7F76C5594}
- Is CFR Compliant : false
- Original data has not been modified.
Remarks:
- User: CD
- Date: 2020/11/27
- Instrument: 0547
- DetectorType: LAAPD
- DichOS Calibration Correction Curve: 0547/2
- HV (CDDC channel): 0 v
- Time per point: 1 s
- Description: Sample 1
- Concentration: 0.6500mg/mL MeOH
- Pathlength: 1 mm
- Temperature: 20℃

Settings:
- Time-per-point: 1s (25us x 40000)
- SE
- Wavelength: 195nm - 400nm
- Step Size: 1nm
- Bandwidth: 1nm

Figure S39. CD and UV spectrum of compound **4**

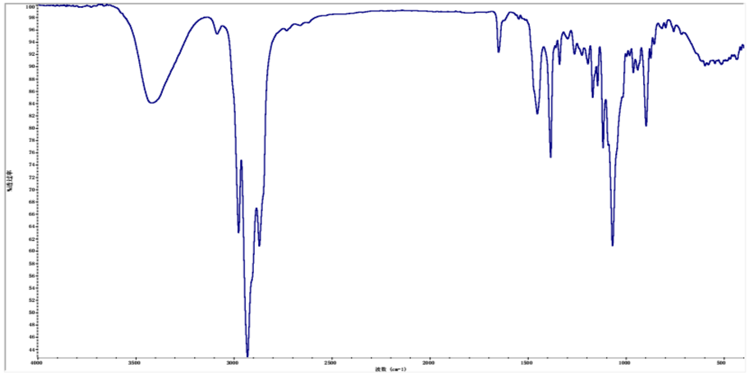

Sample Name: YMX-11-12-7-4-3
KBr压片
采集时间: 星期四 10月 29 10:22:16 2020 (GMT+08:00)
仪器型号: NICOLET iS10
Software version: OMNIC 9.8.372

样品扫描次数: 16
背景扫描次数: 16
分辨率: 4.000
采样增益: 1.0
动镜速度: 0.4747
光阑: 80.00

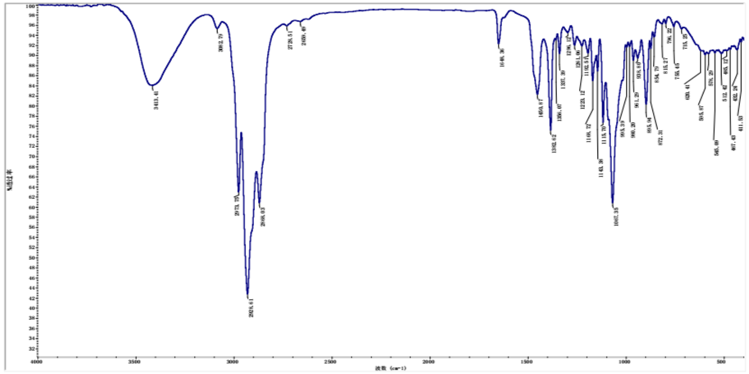

Sample Name: YMX-11-12-7-4-3
KBr压片
采集时间: 星期四 10月 29 10:22:16 2020 (GMT+08:00)
仪器型号: NICOLET iS10
Software version: OMNIC 9.8.372

样品扫描次数: 16
背景扫描次数: 16
分辨率: 4.000
采样增益: 1.0
动镜速度: 0.4747
光阑: 80.00

星期四 10月 29 10:52:47 2020 (GMT+08:00)
标峰:
谱图: YMX-11-12-7-4-3
范围: 4000.00 400.00
（绝对）阈值: 97.382
灵敏度: 99
峰表:

峰位置:	411.53	强度:	92.625
峰位置:	432.24	强度:	91.099
峰位置:	453.24	强度:	91.639
峰位置:	467.43	强度:	91.155
峰位置:	485.12	强度:	90.882
峰位置:	512.42	强度:	90.304
峰位置:	545.09	强度:	90.458
峰位置:	578.29	强度:	90.285
峰位置:	595.87	强度:	90.092
峰位置:	715.25	强度:	95.228
峰位置:	755.45	强度:	95.663
峰位置:	796.22	强度:	96.297
峰位置:	815.27	强度:	96.160
峰位置:	854.79	强度:	93.601
峰位置:	872.31	强度:	91.325
峰位置:	895.94	强度:	80.277
峰位置:	938.80	强度:	89.396
峰位置:	961.29	强度:	88.930
峰位置:	980.20	强度:	91.718
峰位置:	995.39	强度:	91.590
峰位置:	1067.35	强度:	60.756
峰位置:	1115.70	强度:	76.698
峰位置:	1143.38	强度:	86.808
峰位置:	1168.72	强度:	84.945
峰位置:	1192.51	强度:	90.372
峰位置:	1223.12	强度:	91.797
峰位置:	1261.06	强度:	91.989
峰位置:	1296.12	强度:	94.483
峰位置:	1337.39	强度:	90.320
峰位置:	1356.07	强度:	93.043
峰位置:	1382.62	强度:	75.159
峰位置:	1450.87	强度:	82.245
峰位置:	1648.36	强度:	92.241
峰位置:	2659.49	强度:	96.667
峰位置:	2728.51	强度:	95.803
峰位置:	2868.03	强度:	60.744
峰位置:	2928.61	强度:	42.689
峰位置:	2973.75	强度:	62.895
峰位置:	3082.79	强度:	95.265
峰位置:	3413.41	强度:	84.019

Figure S40. IR spectrum of compound **4**

Rudolph Research Analytical

This sample was measured on an Autopol VI, Serial #91058
Manufactured by Rudolph Research Analytical, Hackettstown, NJ, USA.

Measurement Date : Thursday, 26-NOV-2020

Set Temperature : OFF

Time Delay : Disabled

Delay between Measurement : Disabled

n	Average	Std.Dev.	% RSD	Maximum	Minimum
5	-24.66	0.07	-0.28	-24.56	-24.72

S.No	Sample ID	Time	Result	Scale	OR °Arc	WLG.nm	Lg.mm	Conc.g/100ml	Temp.
1	YMX-11-12-7-4-3	02:54:20 PM	-24.72	SR	-0.0618	589	100.00	0.250	22.6
2	YMX-11-12-7-4-3	02:54:29 PM	-24.68	SR	-0.0617	589	100.00	0.250	22.6
3	YMX-11-12-7-4-3	02:54:37 PM	-24.72	SR	-0.0618	589	100.00	0.250	22.6
4	YMX-11-12-7-4-3	02:54:45 PM	-24.64	SR	-0.0616	589	100.00	0.250	22.6
5	YMX-11-12-7-4-3	02:54:53 PM	-24.56	SR	-0.0614	589	100.00	0.250	22.6

Figure S41. $[\alpha]^{25}_{D}$ of compound **4**

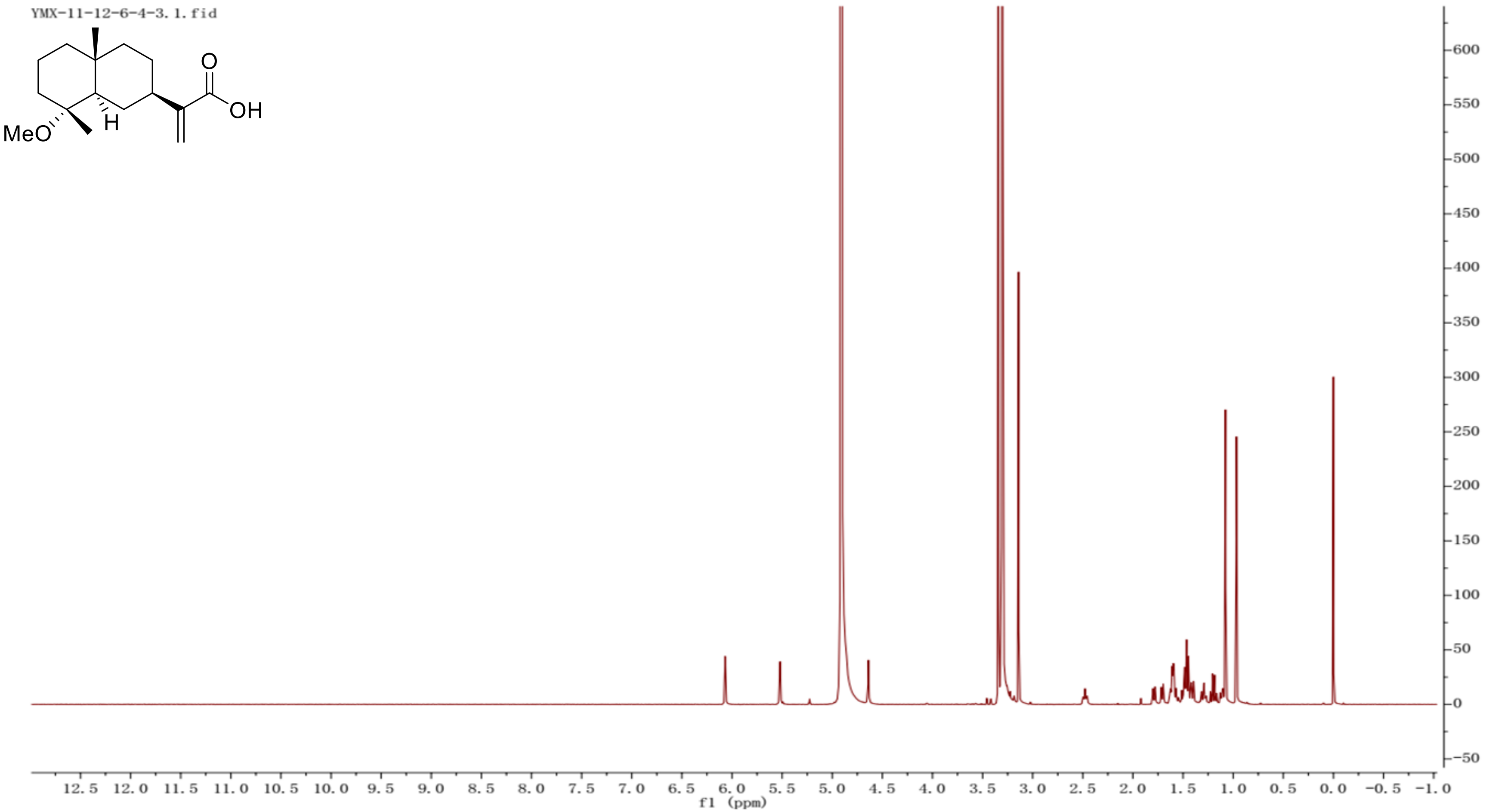

Figure S42. ^{1}H NMR spectrum of compound **5** (CD_3OD, 600 MHz)

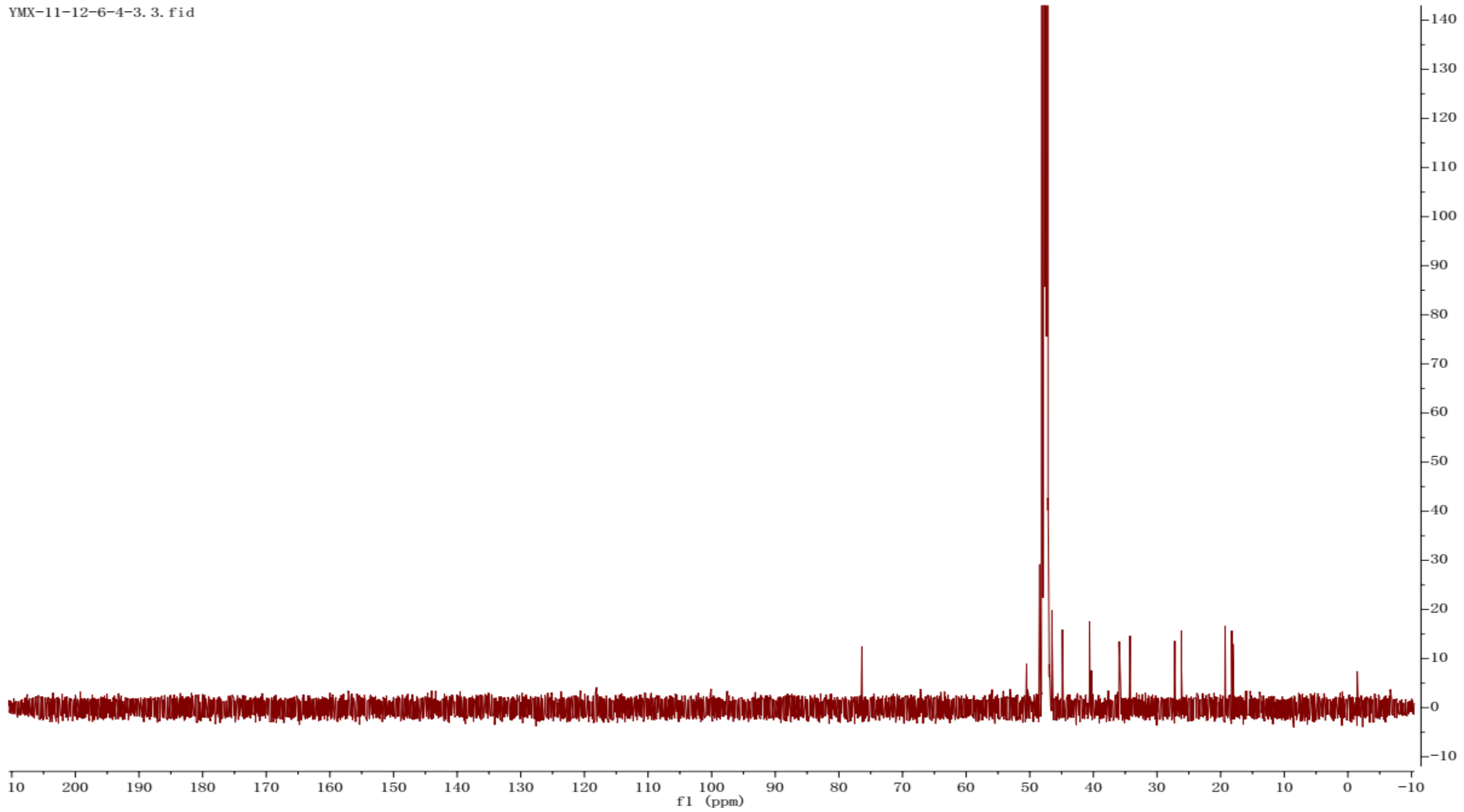

Figure S43. ^{13}C NMR spectrum of compound **5** (CD_3OD, 150 MHz)

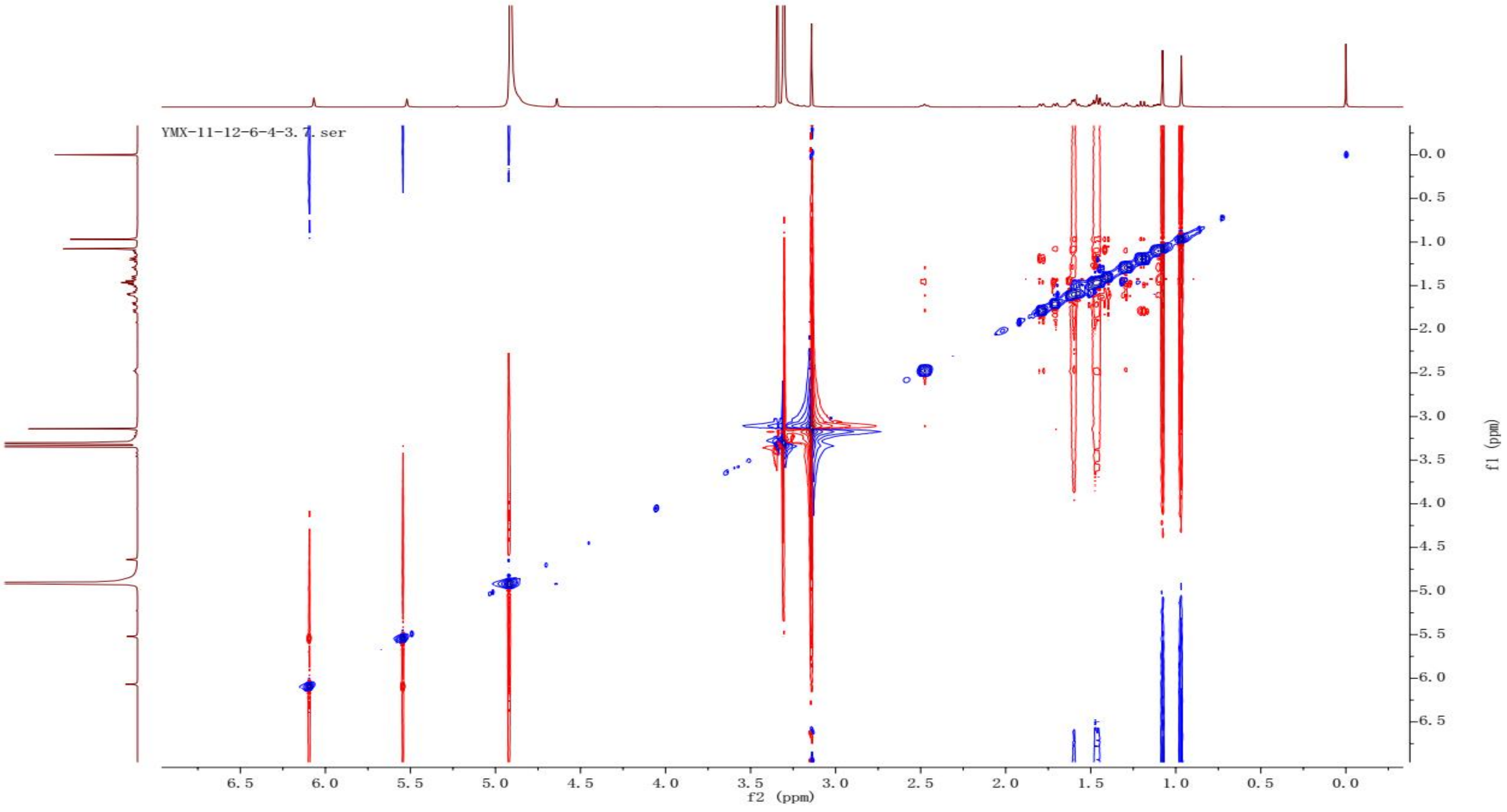

Figure S44. ROESY spectrum of compound **5** (CD_3OD, 600 MHz)

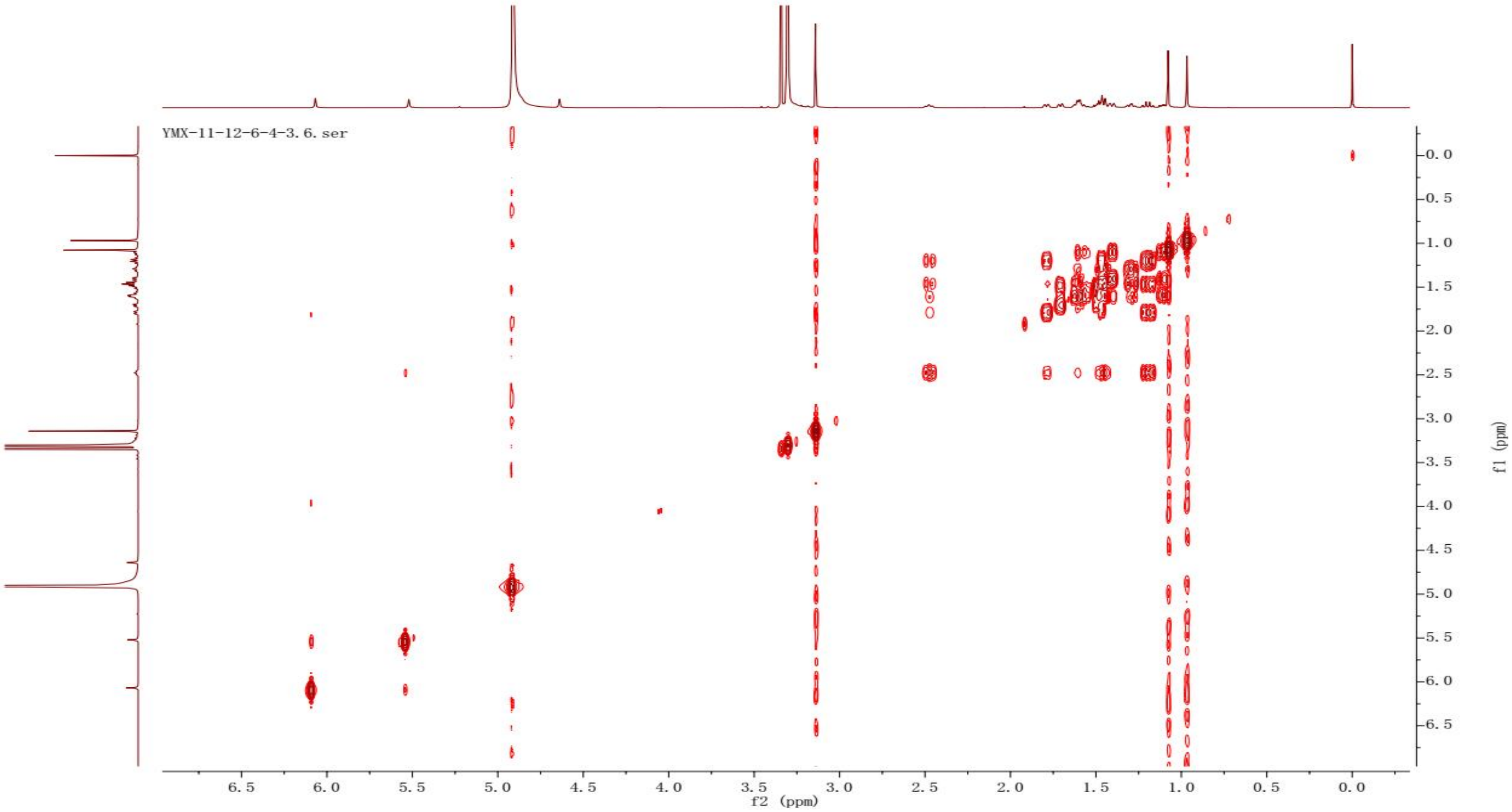

Figure S45. ^{1}H-^{1}H COSY spectrum of compound **5** (CD_3OD, 600 MHz)

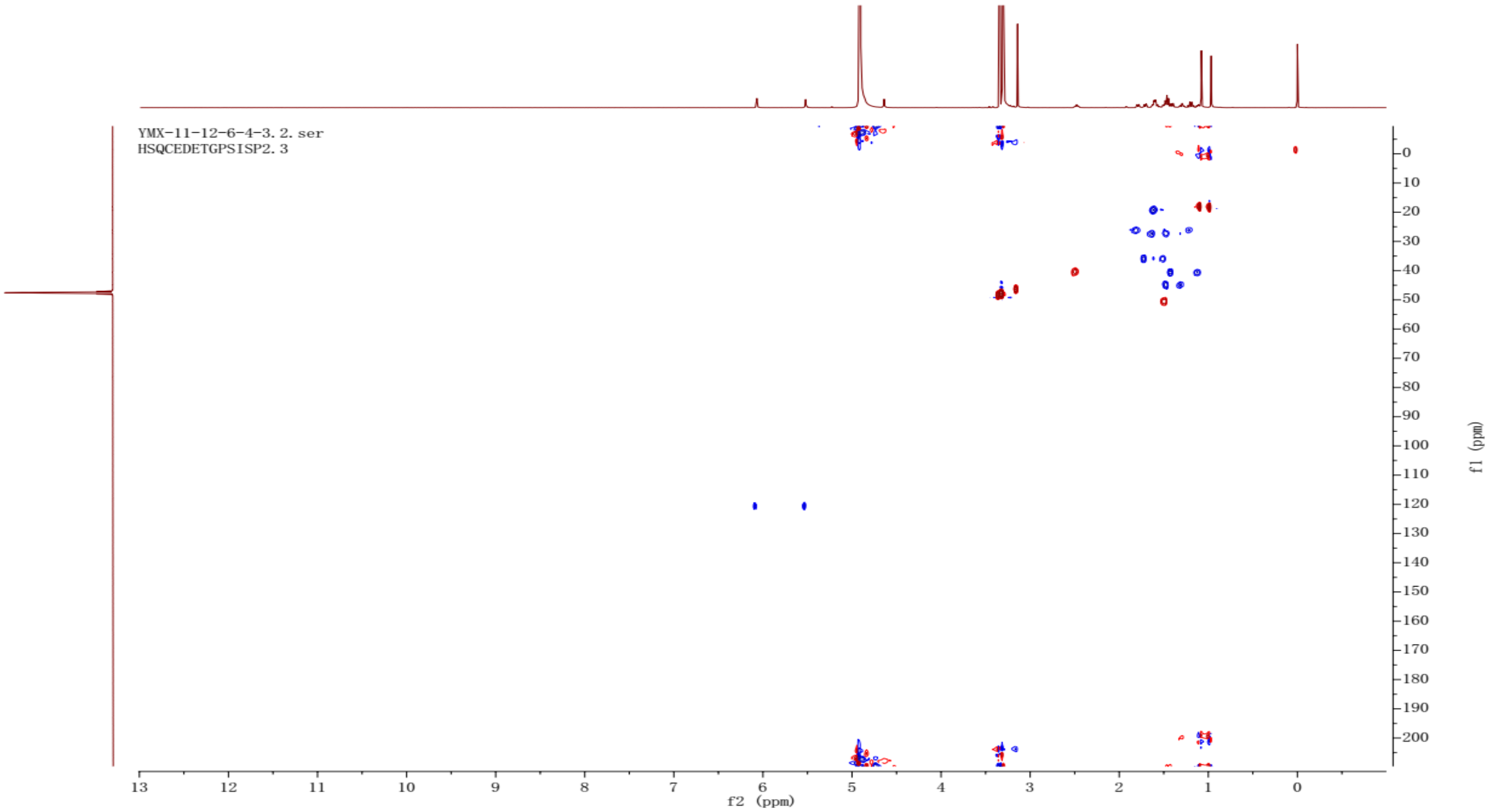

Figure S46. HSQC spectrum of compound **5** (CD_3OD, 600 MHz)

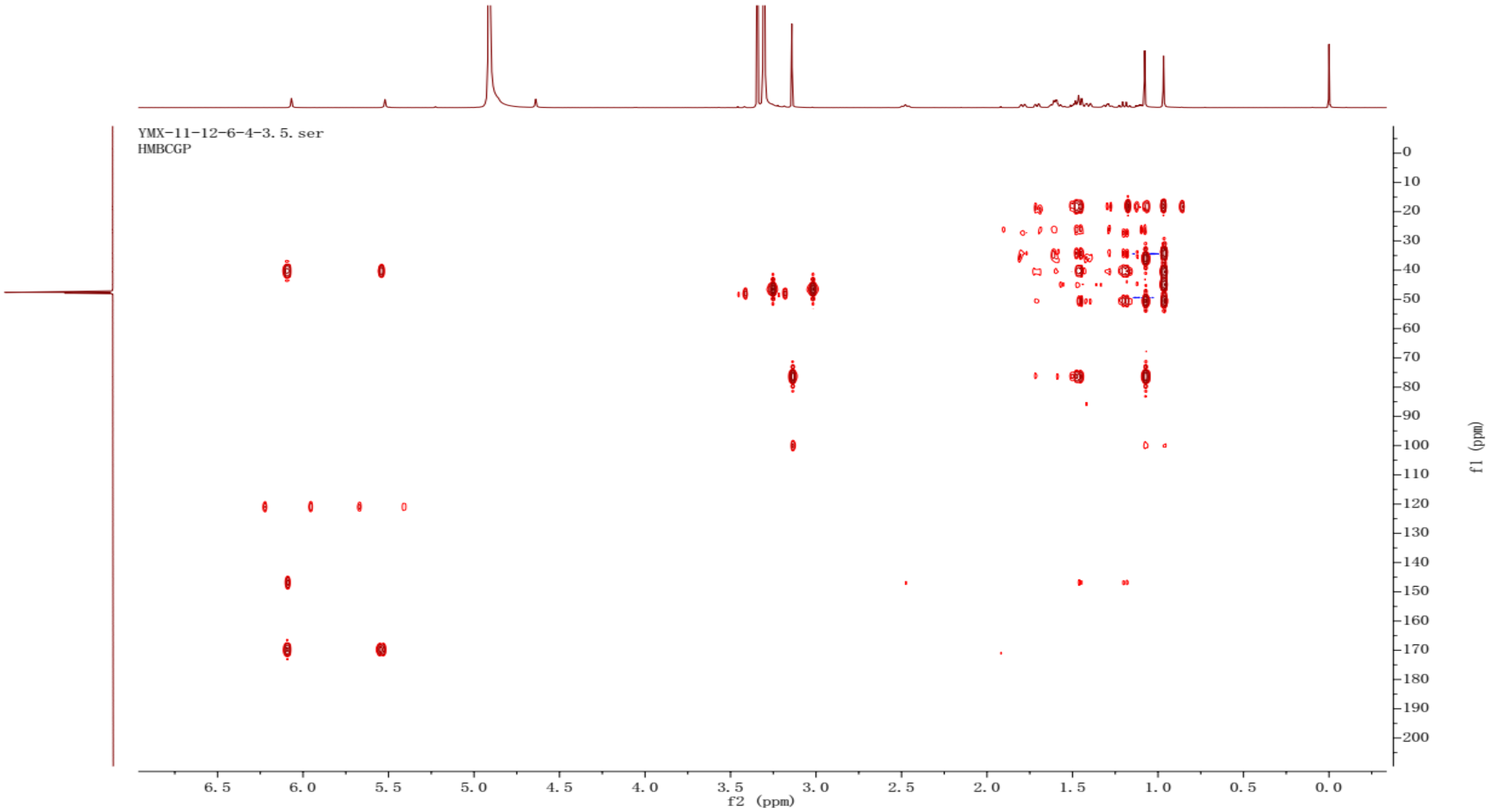

Figure S47. HMBC spectrum of compound **5** (CD_3OD, 600 MHz)

Qualitative Analysis Report

**Data Filename**	YMX-11-12-6-4-3.d	**Sample Name**	YMX-11-12-6-4-3
**Sample Type**	Sample	**Position**	p1b7
**Instrument Name**	Instrument 1	**User Name**	Q-TOF-HP\Q-TOF
**Acq Method**	default20190111-ms.m	**Acquired Time**	1/12/2020 3:56:11 PM
**IRM Calibration Status**	Success	**DA Method**	1.m
**Comment**			

Sample Group **Info.**
Acquisition SW Version 6200 series TOF/6500 series Q-TOF B.05.01 (B5125.3)

User Chromatograms

Fragmentor Voltage 155 **Collision Energy** 0 **Ionization Mode** ESI

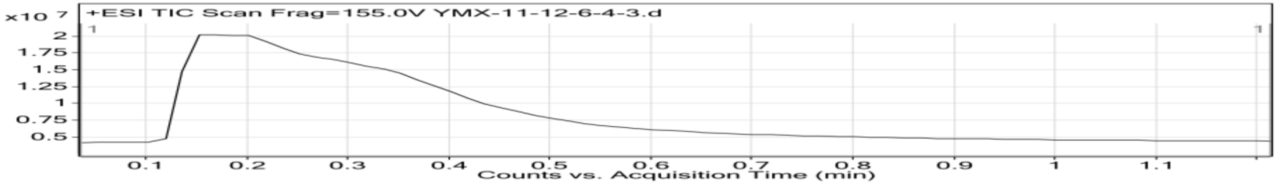

User Spectra

Fragmentor Voltage	Collision Energy	Ionization Mode
155	0	ESI

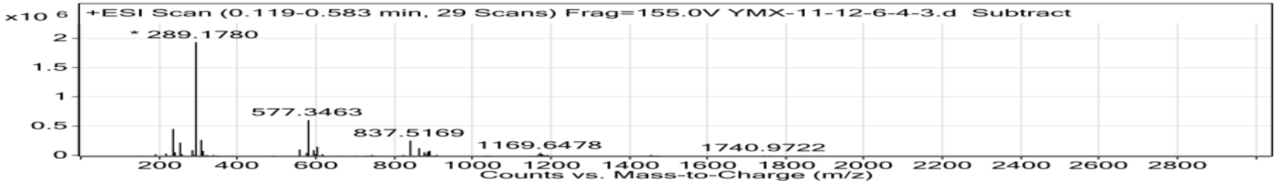

Peak List

m/z	z	Abund
235.1682	1	466617.69
252.1947	1	229973.59
289.178	1	1947617.25
290.1797	1	470698.53
305.1497	1	283320.63
577.3463	1	622858.25
578.3492	1	192049.98
599.3274	1	159994.36

Figure S48. HRESI spectrum of compound **5**

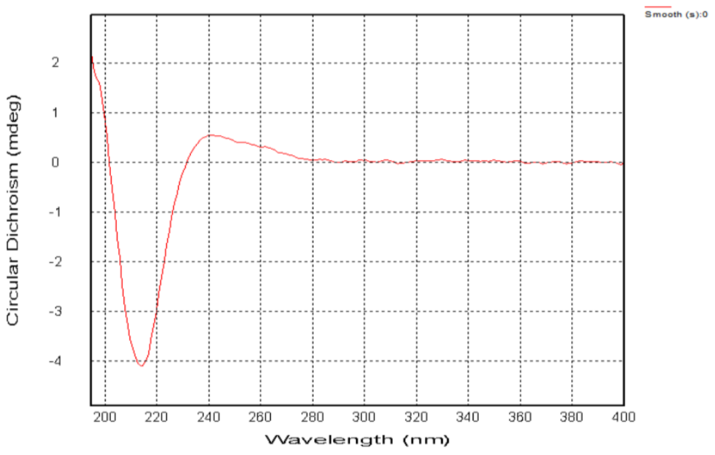

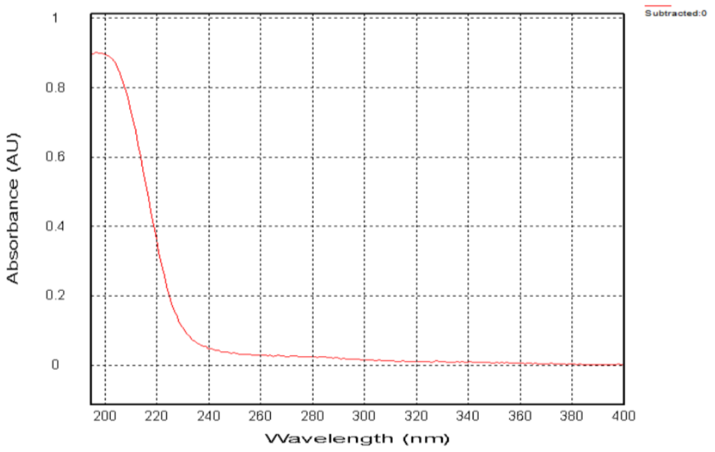

File: YMX-11-12-6-4-3 (195-400 nm) 20112717.dsx
ProBinaryX
Attributes :
- Time Stamp :Fri Nov 27 19:34:36 2020

- File ID : {E3C61445-D6E4-4321-8A56-F058D491F92A}
- Is CFR Compliant : false
- Original data has not been modified.

Remarks:
- User: CD
- Date: 2020/11/27
- Instrument: 0547
- DetectorType: LAAPD
- DichOS Calibration Correction Curve: 0547/2
- HV (CDDC channel): 0 v
- Time per point: 1 s
- Description: Sample 1
- Concentration: 0.3300mg/mL MeOH
- Pathlength: 1 mm
- Temperature: 20℃

Settings:
- Time-per-point: 1s (25us x 40000)
- SE
- Wavelength: 195nm - 400nm
- Step Size: 1nm
- Bandwidth: 1nm

Figure S49. CD and UV spectrum of compound **5**

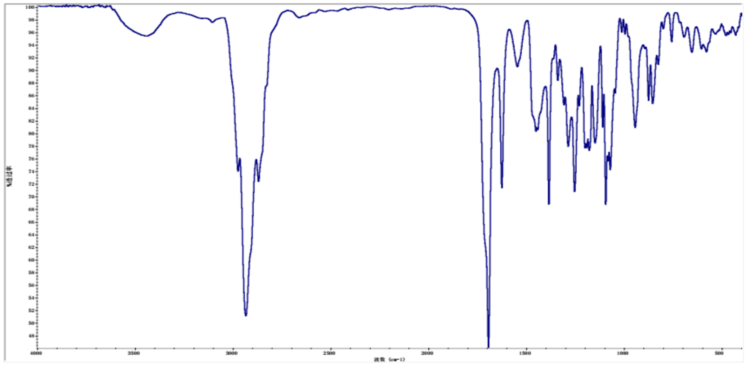

Sample Name: YMX-11-12-6-4-3
KBr压片
采集时间: 星期三 10月 28 15:50:22 2020 (GMT+08:00)
仪器型号: NICOLET iS10
Software version: OMNIC 9.8.372

样品扫描次数: 16
背景扫描次数: 16
分辨率: 4.000
采样增益: 1.0
动镜速度: 0.4747
光阑: 80.00

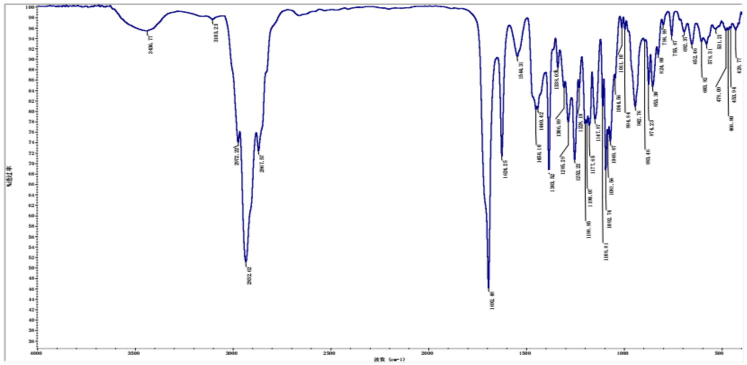

Sample Name: YMX-11-12-6-4-3
KBr压片
采集时间: 星期三 10月 28 15:50:22 2020 (GMT+08:00)
仪器型号: NICOLET iS10
Software version: OMNIC 9.8.372

样品扫描次数: 16
背景扫描次数: 16
分辨率: 4.000
采样增益: 1.0
动镜速度: 0.4747
光阑: 80.00

星期三 10月 28 16:17:58 2020 (GMT+08:00)
标峰:
谱图: YMX-11-12-6-4-3
范围: 4000.00 400.00
(绝对) 阈值: 98.027
灵敏度: 99
峰表:

峰位置:	412.82	强度:	96.662
峰位置:	428.77	强度:	95.522
峰位置:	453.94	强度:	95.875
峰位置:	466.80	强度:	95.609
峰位置:	478.09	强度:	95.467
峰位置:	531.21	强度:	95.695
峰位置:	578.31	强度:	92.856
峰位置:	603.92	强度:	93.347
峰位置:	652.46	强度:	92.811
峰位置:	692.37	强度:	95.215
峰位置:	755.87	强度:	94.485
峰位置:	798.99	强度:	96.532
峰位置:	824.80	强度:	90.853
峰位置:	853.30	强度:	84.659
峰位置:	874.23	强度:	85.192
峰位置:	893.46	强度:	93.418
峰位置:	942.76	强度:	80.892
峰位置:	994.84	强度:	95.705
峰位置:	1011.10	强度:	95.968
峰位置:	1044.56	强度:	86.363
峰位置:	1069.87	强度:	74.184
峰位置:	1081.56	强度:	75.599
峰位置:	1092.74	强度:	68.662
峰位置:	1108.81	强度:	80.982
峰位置:	1147.87	强度:	78.456
峰位置:	1177.65	强度:	77.247
峰位置:	1190.07	强度:	77.700
峰位置:	1198.95	强度:	77.649
峰位置:	1228.10	强度:	84.290
峰位置:	1252.22	强度:	70.643
峰位置:	1285.29	强度:	77.882
峰位置:	1306.89	强度:	84.494
峰位置:	1338.03	强度:	88.230
峰位置:	1383.52	强度:	68.059
峰位置:	1440.42	强度:	80.577
峰位置:	1450.10	强度:	80.296
峰位置:	1544.31	强度:	90.498
峰位置:	1624.25	强度:	71.351
峰位置:	1692.48	强度:	46.060
峰位置:	2867.57	强度:	72.352
峰位置:	2932.62	强度:	51.049
峰位置:	2972.22	强度:	73.976
峰位置:	3103.23	强度:	97.566
峰位置:	3436.77	强度:	95.382

Figure S50. IR spectrum of compound **5**

Rudolph Research Analytical

This sample was measured on an Autopol VI, Serial #91058
Manufactured by Rudolph Research Analytical, Hackettstown, NJ, USA.

Measurement Date : Thursday, 26-NOV-2020

Set Temperature : OFF

Time Delay : Disabled

Delay between Measurement : Disabled

n	Average	Std.Dev.	% RSD	Maximum	Minimum
5	-29.43	0.11	-0.37	-29.33	-29.60

S.No	Sample ID	Time	Result	Scale	OR °Arc	WLG.nm	Lg.mm	Conc.g/100ml	Temp.
1	YMX-11-12-6-4-3	02:24:56 PM	-29.60	SR	-0.0444	589	100.00	0.150	22.8
2	YMX-11-12-6-4-3	02:25:04 PM	-29.33	SR	-0.0440	589	100.00	0.150	22.8
3	YMX-11-12-6-4-3	02:25:12 PM	-29.33	SR	-0.0440	589	100.00	0.150	22.8
4	YMX-11-12-6-4-3	02:25:20 PM	-29.47	SR	-0.0442	589	100.00	0.150	22.8
5	YMX-11-12-6-4-3	02:25:28 PM	-29.40	SR	-0.0441	589	100.00	0.150	22.9

Figure S51 $[\alpha]^{25}_{D}$ of compound **5**

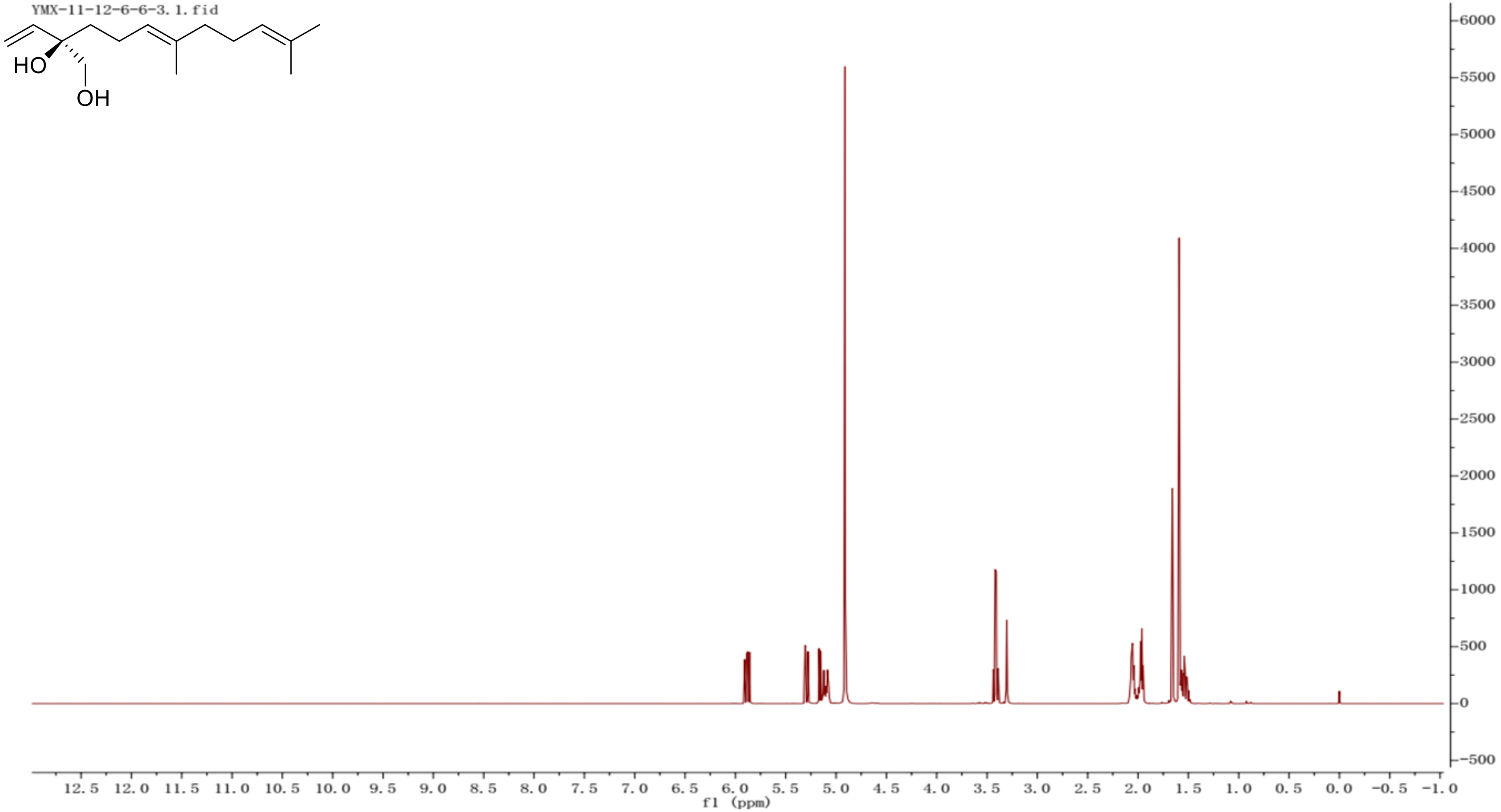

Figure S52. ^{1}H NMR spectrum of compound **6** (CD_3OD, 600 MHz)

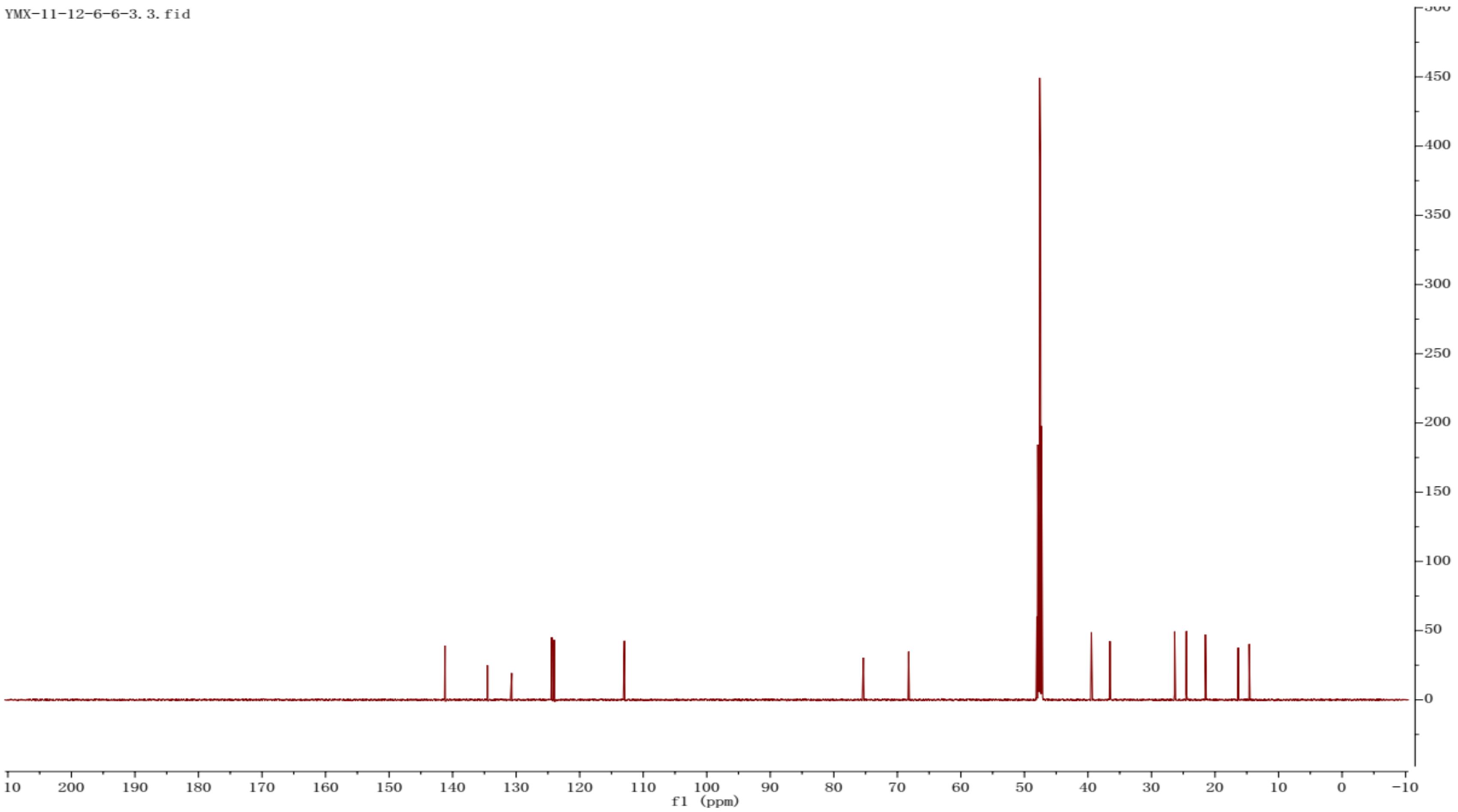

Figure S53. ^{13}C NMR spectrum of compound **6** (CD_3OD, 150 MHz)

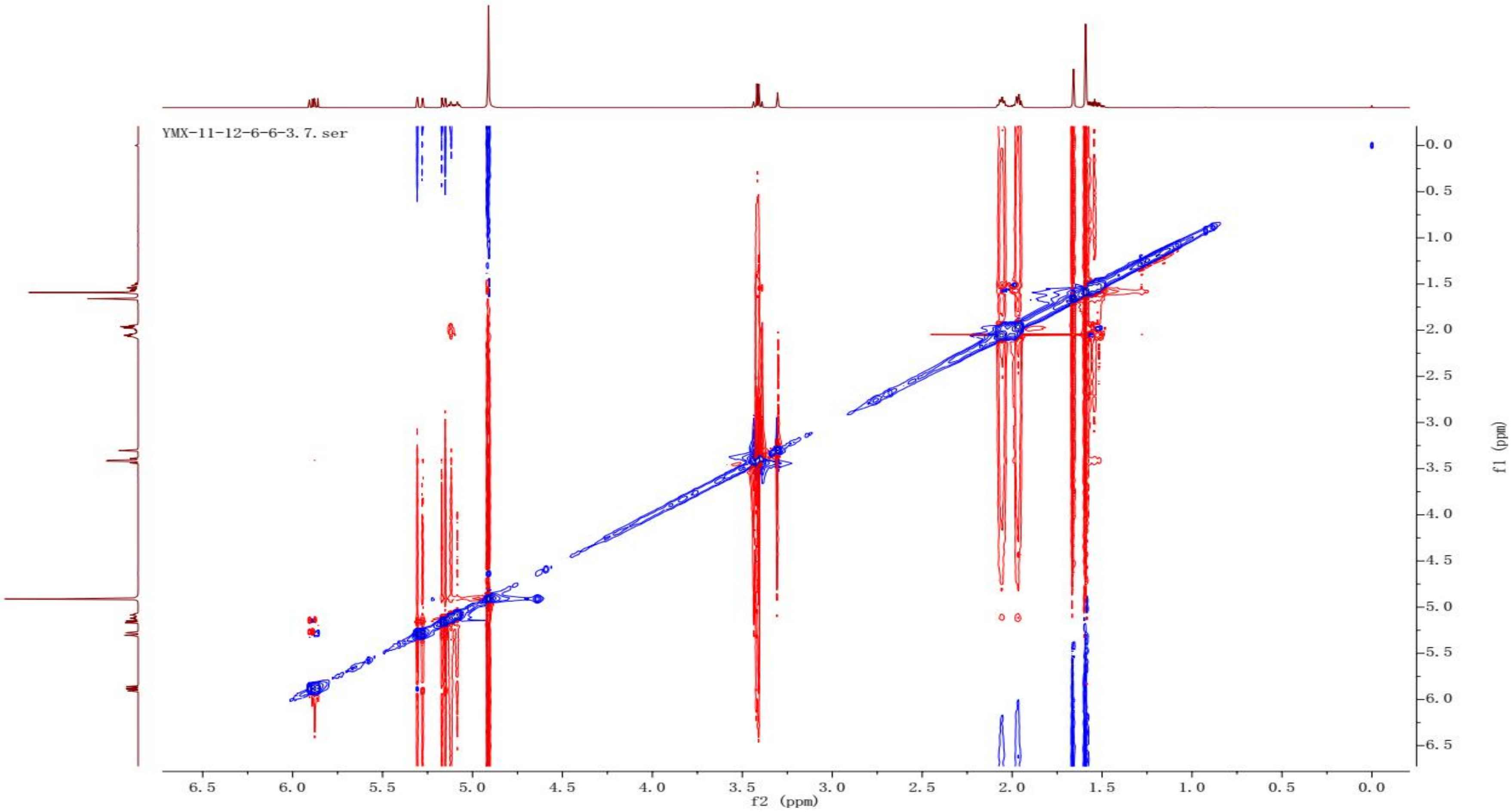

Figure S54. ROESY spectrum of compound **6** (CD_3OD, 600 MHz)

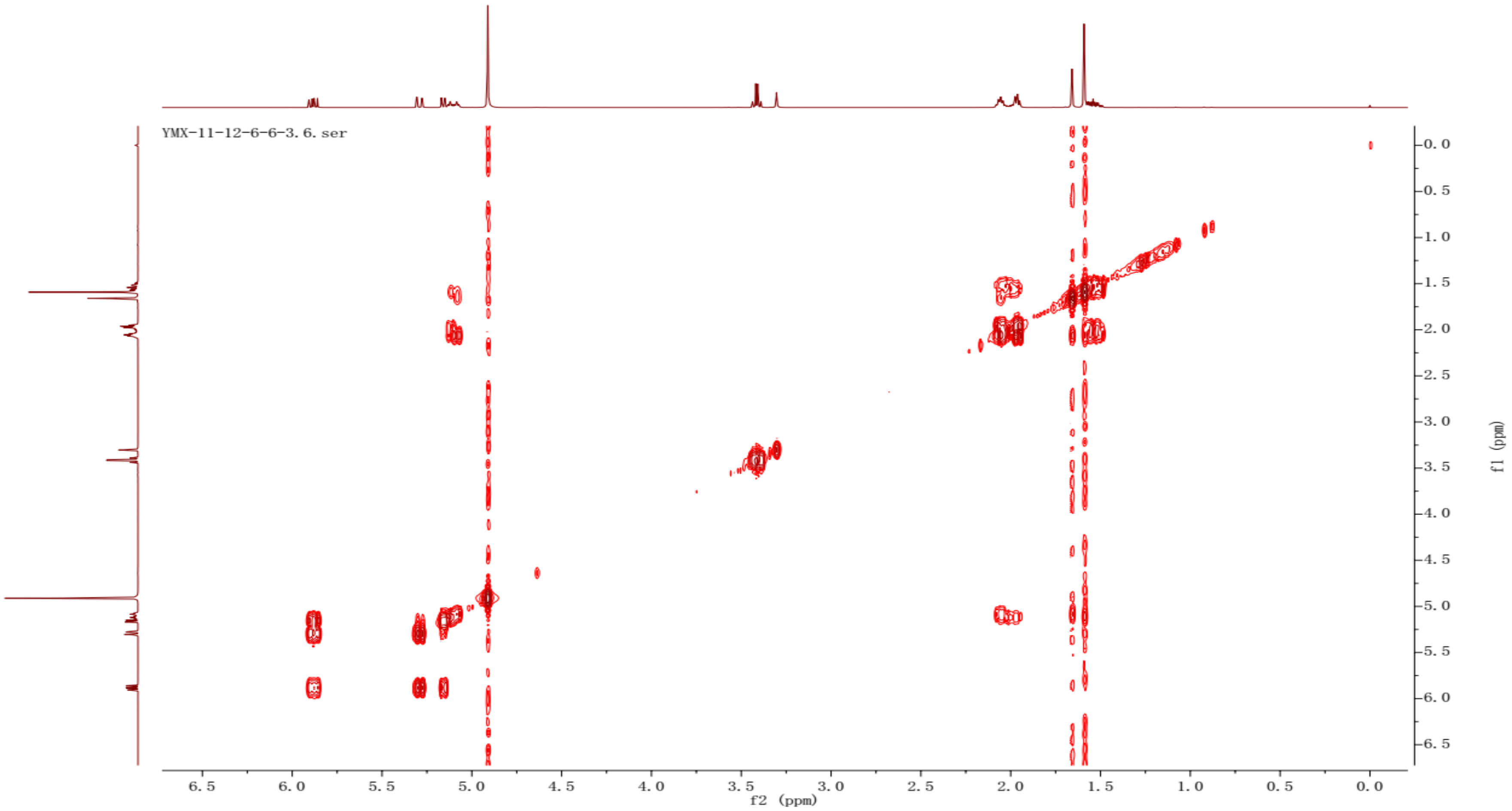

Figure S55. ^{1}H-^{1}H COSY spectrum of compound **6** (CD_3OD, 600 MHz)

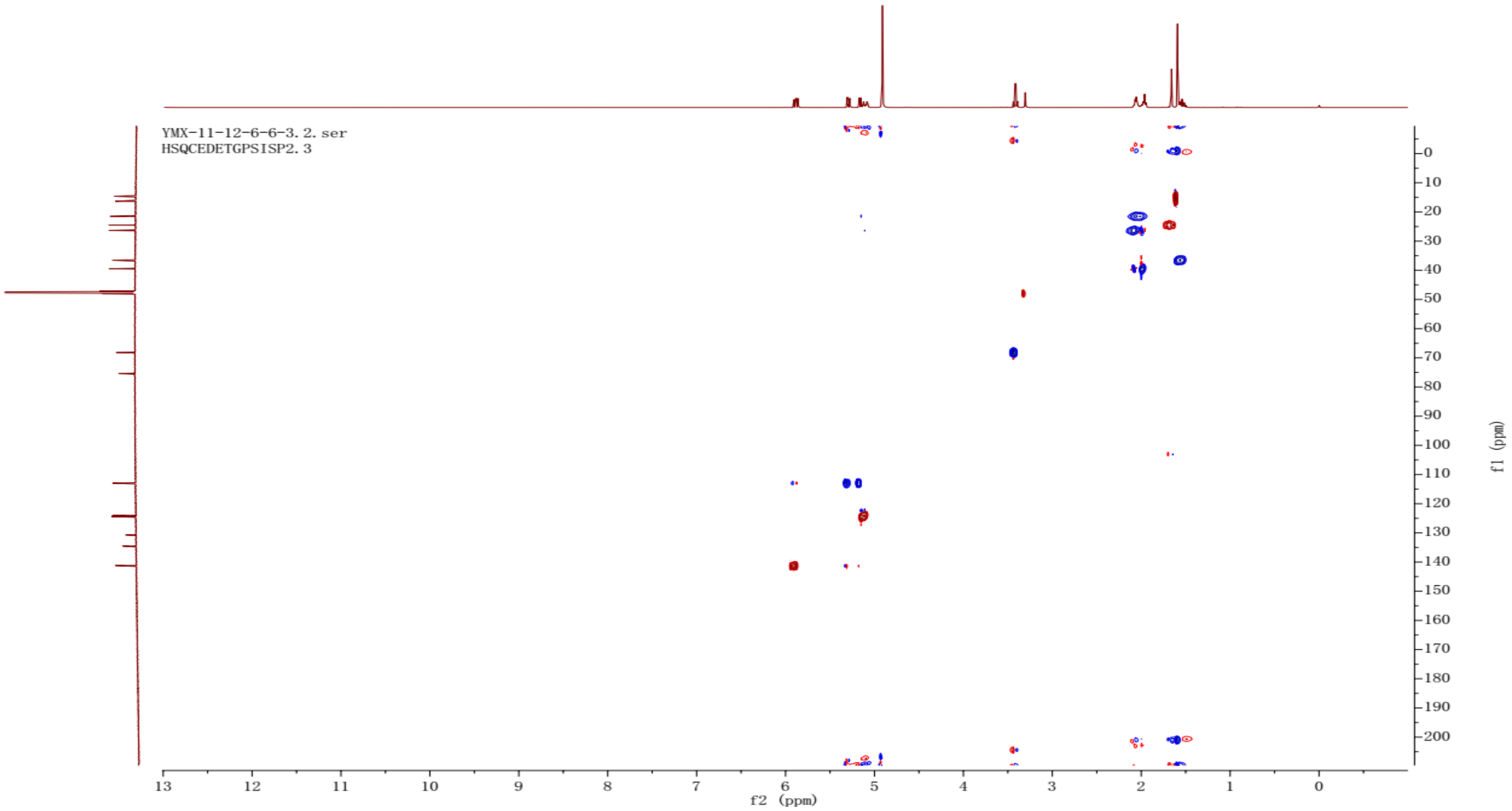

Figure S56. HSQC spectrum of compound **6** (CD_3OD, 600 MHz)

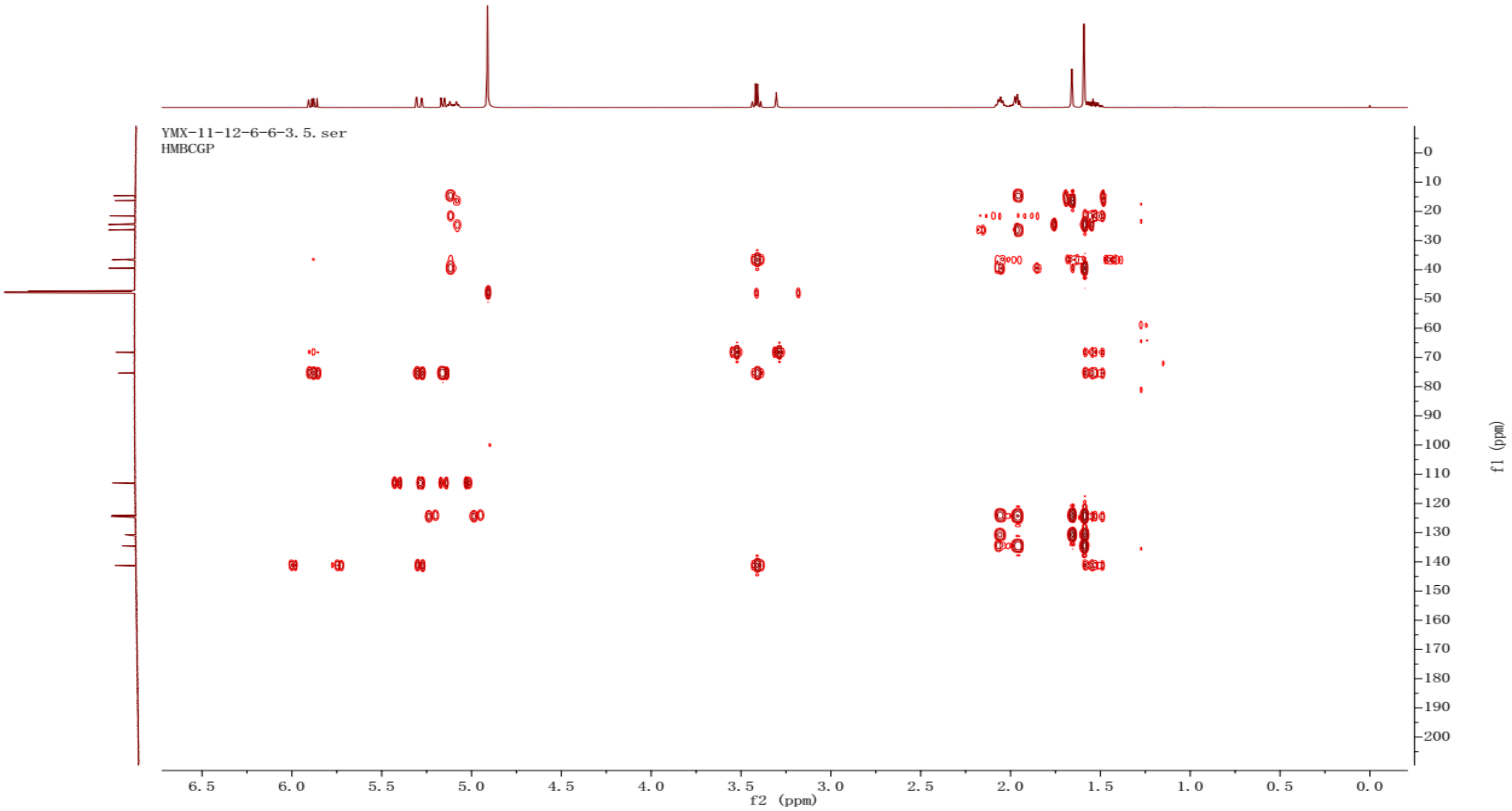

Figure S57. HMBC spectrum of compound **6** (CD_3OD, 600 MHz)

Qualitative Analysis Report

**Data Filename**	YMX-11-12-6-6-3.d	**Sample Name**	YMX-11-12-6-6-3
**Sample Type**	Sample	**Position**	p1c1
**Instrument Name**	Instrument 1	**User Name**	Q-TOF-HP\Q-TOF
**Acq Method**	default20190111-ms.m	**Acquired Time**	1/12/2020 4:05:49 PM
**IRM Calibration Status**	Success	**DA Method**	1.m
**Comment**			

Sample Group **Info.**
Acquisition SW Version 6200 series TOF/6500 series Q-TOF B.05.01 (B5125.3)

User Chromatograms

Fragmentor Voltage 155 **Collision Energy** 0 **Ionization Mode** ESI

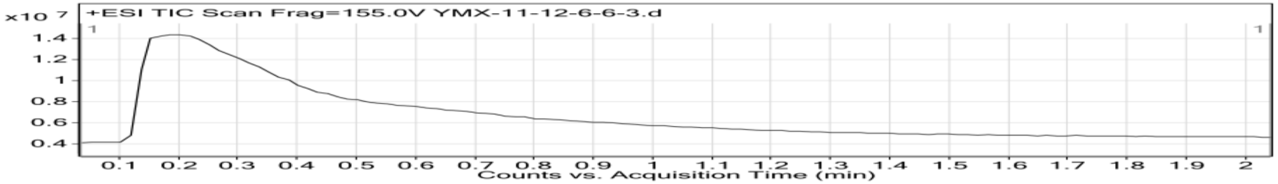

User Spectra

Fragmentor Voltage 155 **Collision Energy** 0 **Ionization Mode** ESI

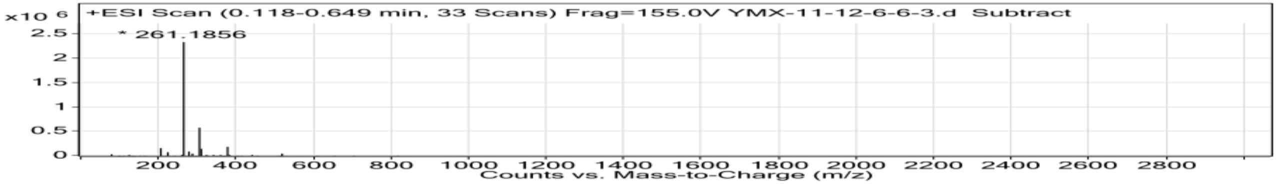

Peak List

m/z	z	Abund
203.1783	1	172827.08
261.1856		2338798.5
261.2774		96010.87
261.3213	1	342364
261.3655		121769.01
262.1851	1	1024942.81
277.1545	1	95900.87
303.1922	1	584750.81

Agilent Technologies Page 1 of 2 Printed at: 4:32 PM on: 4/15/2020

Figure S58. HRESI spectrum of compound **6**

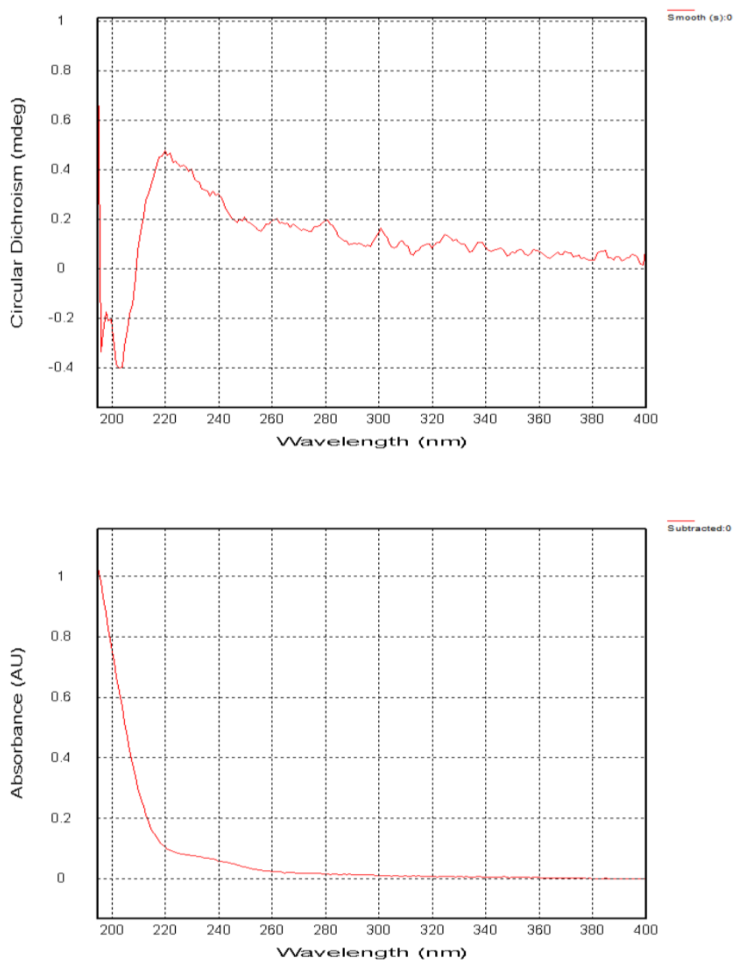

File: YMX-11-12-6-6-3 (195-400 nm) 20112720.dsx
ProBinaryX
Attributes :
- Time Stamp :Fri Nov 27 20:42:05 2020

- File ID : {6F3D91B5-42E8-473d-B607-7EE2ACF6B1BD}
- Is CFR Compliant : false
- Original data has not been modified.

Remarks:
- User: CD
- Date: 2020/11/27
- Instrument: 0547
- DetectorType: LAAPD
- DichOS Calibration Correction Curve: 0547/2
- HV (CDDC channel): 0 v
- Time per point: 1 s
- Description: Sample 1
- Concentration: 0.1500mg/mL MeOH
- Pathlength: 1 mm
- Temperature: 20℃

Settings:
- Time-per-point: 1s (25us x 40000)
- SE
- Wavelength: 195nm - 400nm
- Step Size: 1nm
- Bandwidth: 1nm

Figure S59. CD and UV spectrum of compound **6**

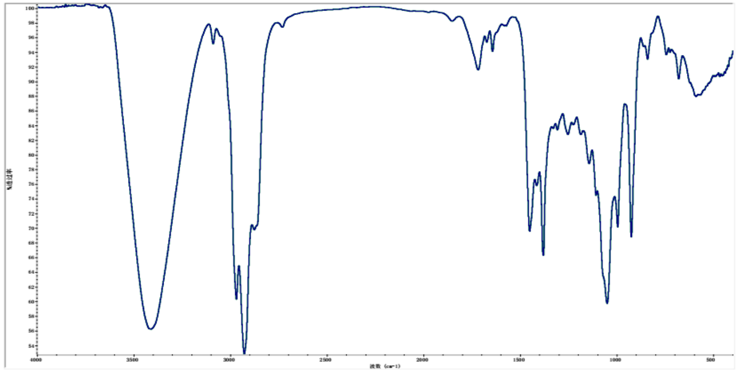

Sample Name: YMX-11-12-6-6-3
KBr压片
采集时间: 星期四 10月 29 11:29:49 2020 (GMT+08:00)
仪器型号: NICOLET iS10
Software version: OMNIC 9.8.372

样品扫描次数: 16
背景扫描次数: 16
分辨率: 4.000
采样增益: 1.0
动镜速度: 0.4747
光阑: 80.00

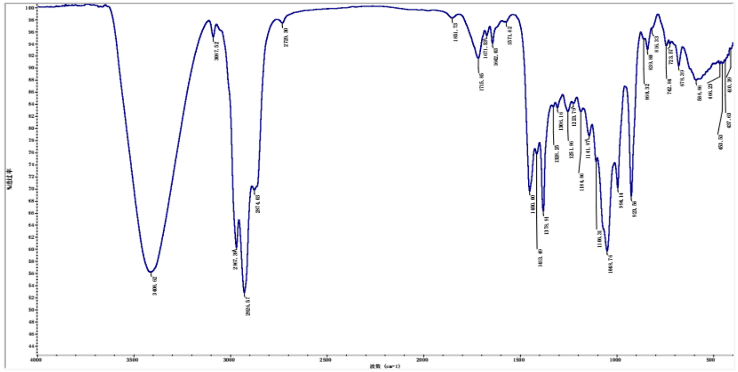

Sample Name: YMX-11-12-6-6-3
KBr压片
采集时间: 星期四 10月 29 11:29:49 2020 (GMT+08:00)
仪器型号: NICOLET iS10
Software version: OMNIC 9.8.372

样品扫描次数: 16
背景扫描次数: 16
分辨率: 4.000
采样增益: 1.0
动镜速度: 0.4747
光阑: 80.00

星期四 10月 29 13:55:28 2020 (GMT+08:00)
标峰:
谱图: YMX-11-12-6-6-3
范围: 4000.00 400.00
（绝对）阈值: 99.389
灵敏度: 99
峰表:

峰位置:	410.39	强度:	93.079
峰位置:	421.03	强度:	92.003
峰位置:	437.63	强度:	91.084
峰位置:	453.53	强度:	90.767
峰位置:	466.23	强度:	90.657
峰位置:	488.75	强度:	90.640
峰位置:	497.17	强度:	90.619
峰位置:	588.86	强度:	87.915
峰位置:	678.39	强度:	90.326
峰位置:	723.57	强度:	93.959
峰位置:	742.84	强度:	93.655
峰位置:	839.80	强度:	93.032
峰位置:	860.32	强度:	94.743
峰位置:	923.56	强度:	68.766
峰位置:	994.14	强度:	70.116
峰位置:	1048.76	强度:	59.646
峰位置:	1106.31	强度:	74.437
峰位置:	1141.87	强度:	78.734
峰位置:	1184.86	强度:	82.714
峰位置:	1223.79	强度:	84.083
峰位置:	1251.86	强度:	82.717
峰位置:	1306.16	强度:	83.359
峰位置:	1328.25	强度:	83.547
峰位置:	1378.91	强度:	66.225
峰位置:	1413.49	强度:	75.733
峰位置:	1450.00	强度:	69.520
峰位置:	1571.62	强度:	97.551
峰位置:	1642.03	强度:	94.060
峰位置:	1671.53	强度:	95.362
峰位置:	1715.85	强度:	91.580
峰位置:	1851.73	强度:	98.214
峰位置:	2729.30	强度:	97.386
峰位置:	2874.03	强度:	69.723
峰位置:	2926.57	强度:	52.769
峰位置:	2967.39	强度:	60.236
峰位置:	3087.52	强度:	95.102
峰位置:	3408.62	强度:	56.161

Figure S60. IR spectrum of compound **6**

Rudolph Research Analytical

This sample was measured on an Autopol VI, Serial #91058
Manufactured by Rudolph Research Analytical, Hackettstown, NJ, USA.

Measurement Date : Thursday, 26-NOV-2020

Set Temperature : OFF

Time Delay : Disabled

Delay between Measurement : Disabled

n	Average	Std.Dev.	% RSD	Maximum	Minimum
5	0.32	0.19	59.37	0.53	0.13

S.No	Sample ID	Time	Result	Scale	OR °Arc	WLG.nm	Lg.mm	Conc.g/100ml	Temp.
1	YMX-11-12-6-6-3	03:01:40 PM	0.13	SR	0.0002	589	100.00	0.150	22.5
2	YMX-11-12-6-6-3	03:01:48 PM	0.47	SR	0.0007	589	100.00	0.150	22.5
3	YMX-11-12-6-6-3	03:01:56 PM	0.13	SR	0.0002	589	100.00	0.150	22.5
4	YMX-11-12-6-6-3	03:02:05 PM	0.33	SR	0.0005	589	100.00	0.150	22.5
5	YMX-11-12-6-6-3	03:02:12 PM	0.53	SR	0.0008	589	100.00	0.150	22.5

Figure S61. $[\alpha]^{25}_{D}$ of compound **6**

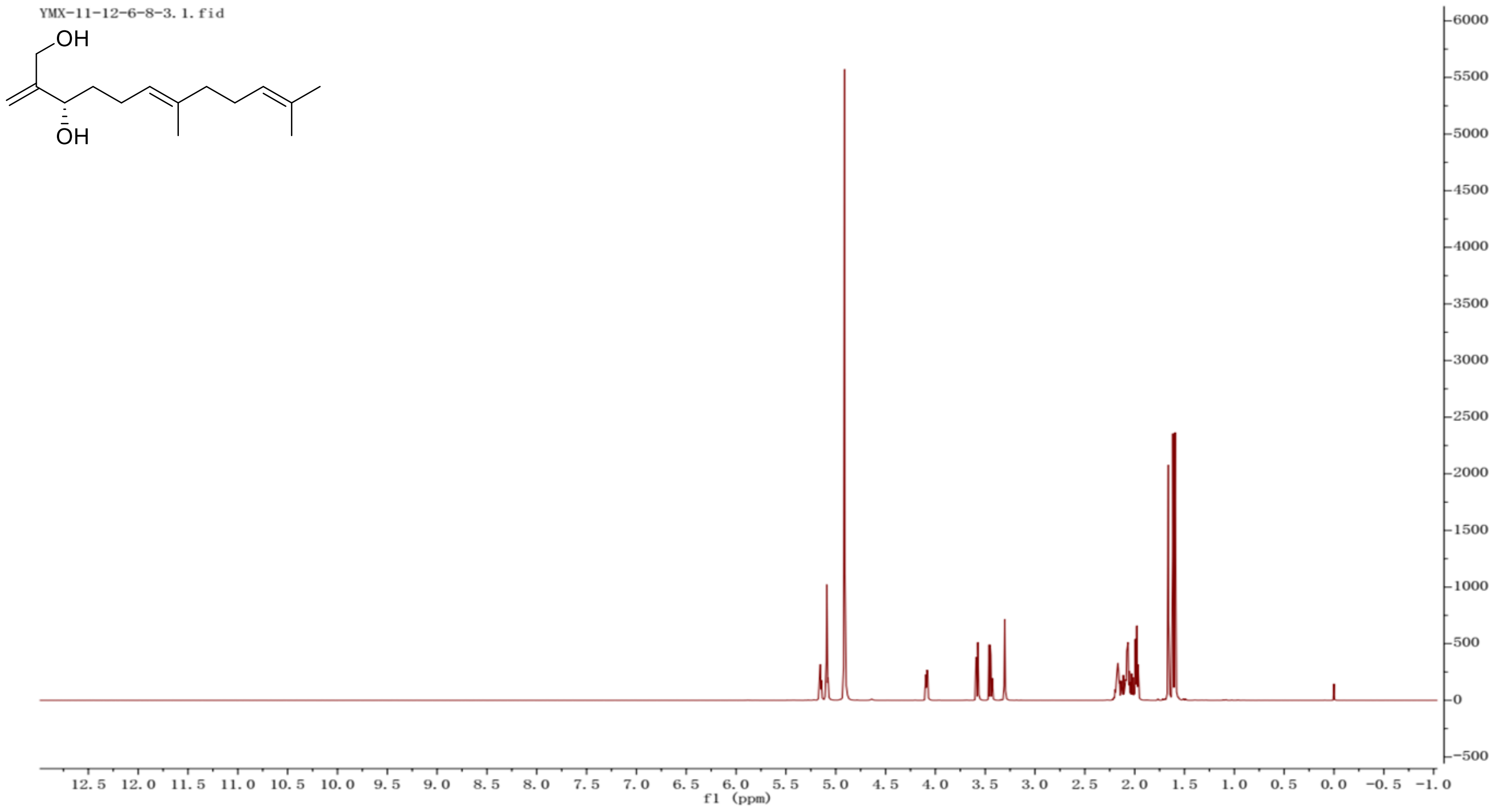

Figure S62. ^{1}H NMR spectrum of compound **7** (CD_3OD, 600 MHz)

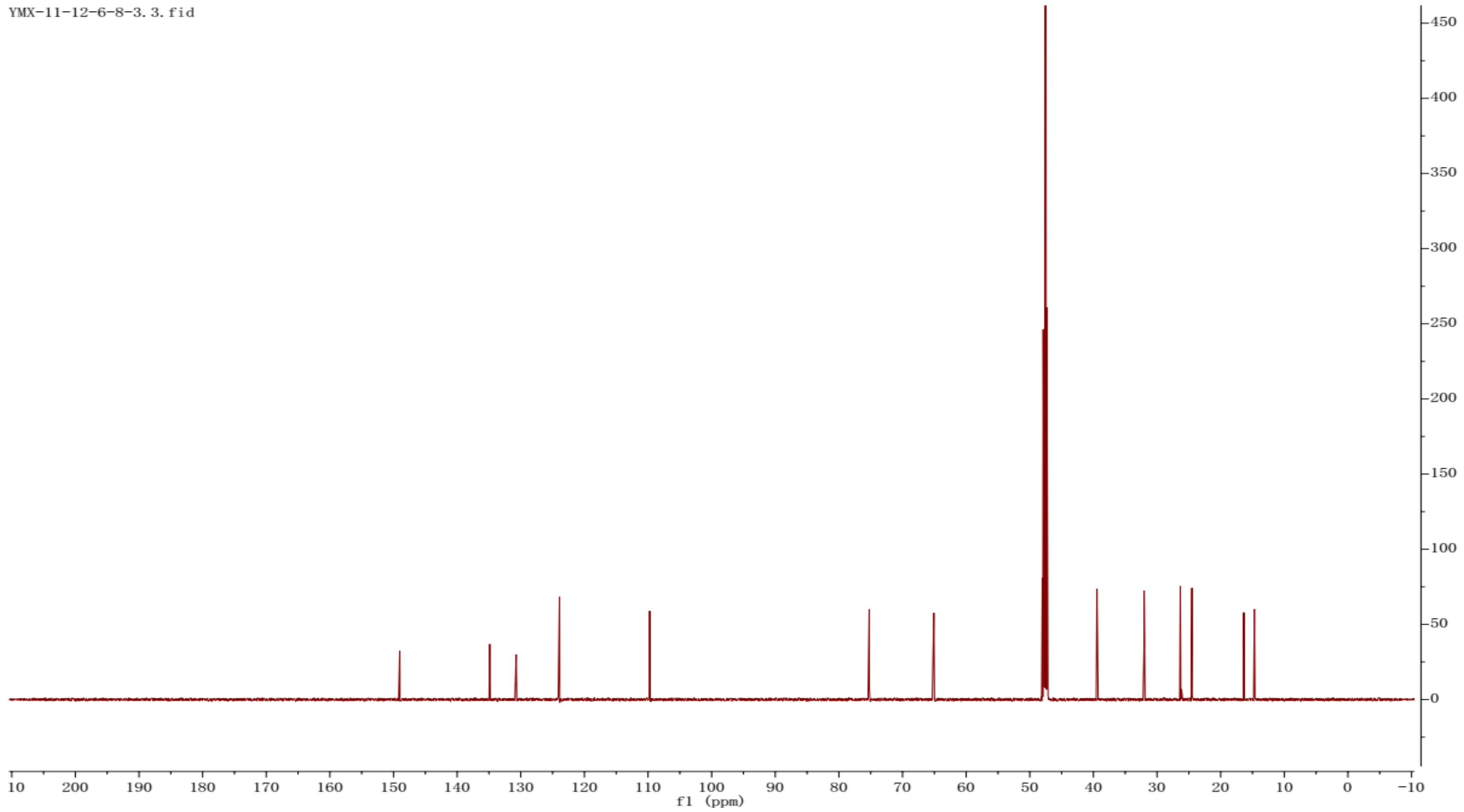

Figure S63. ^{13}C NMR spectrum of compound **7** (CD_3OD, 150 MHz)

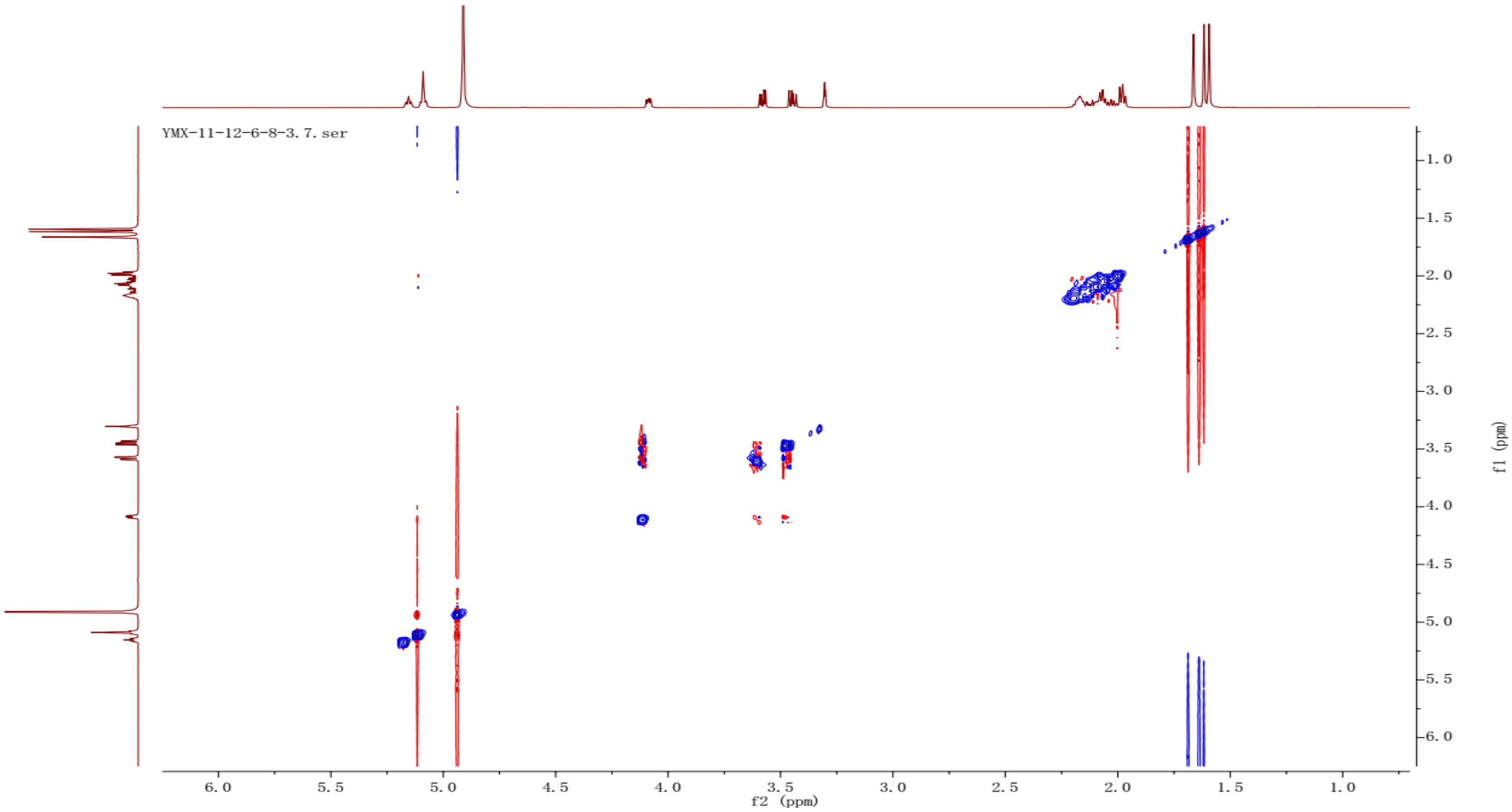

Figure S64. ROESY spectrum of compound **7** (CD_3OD, 600 MHz)

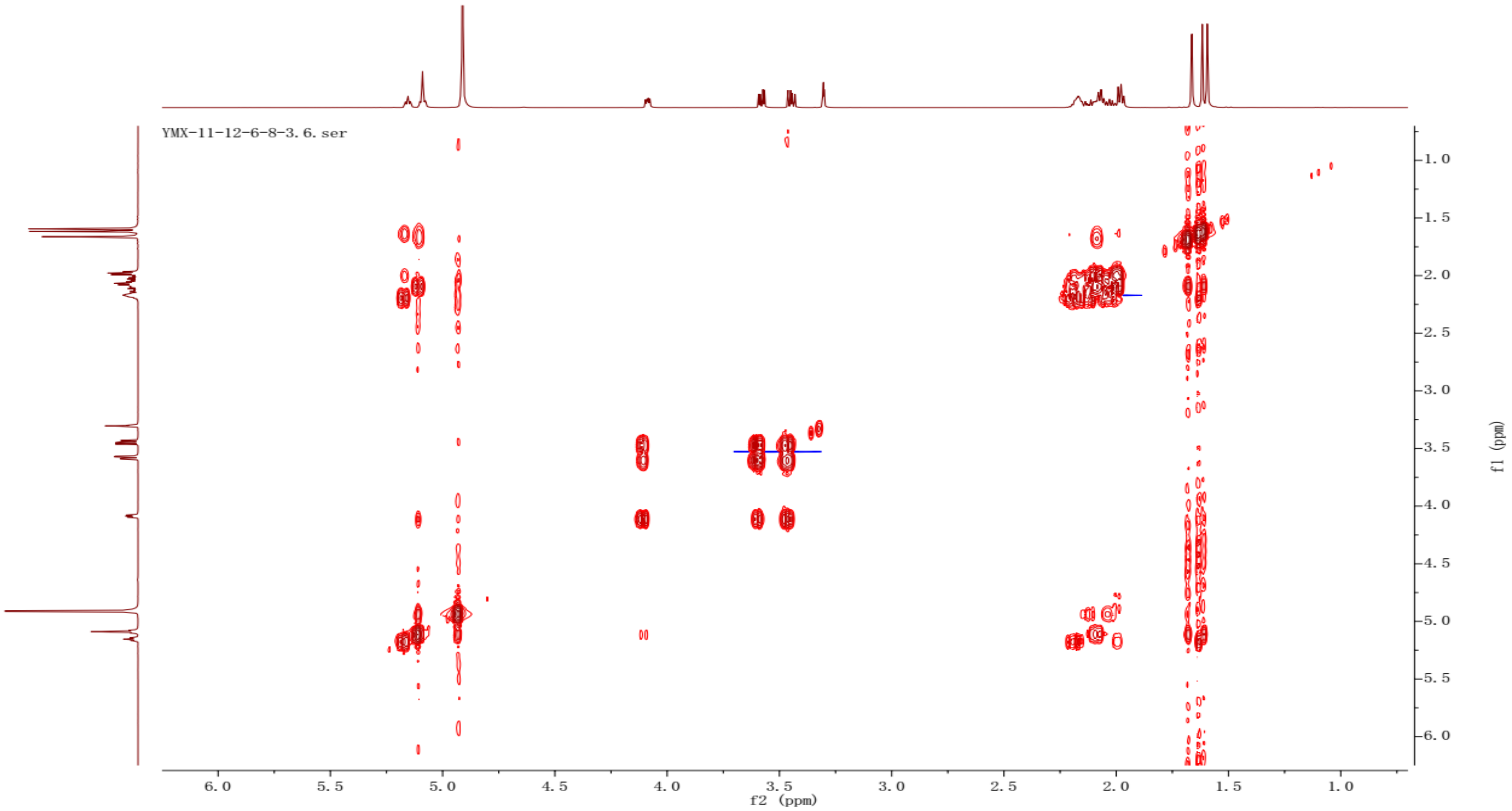

Figure S65. ^{1}H-^{1}H COSY spectrum of compound **7** (CD_3OD, 600 MHz)

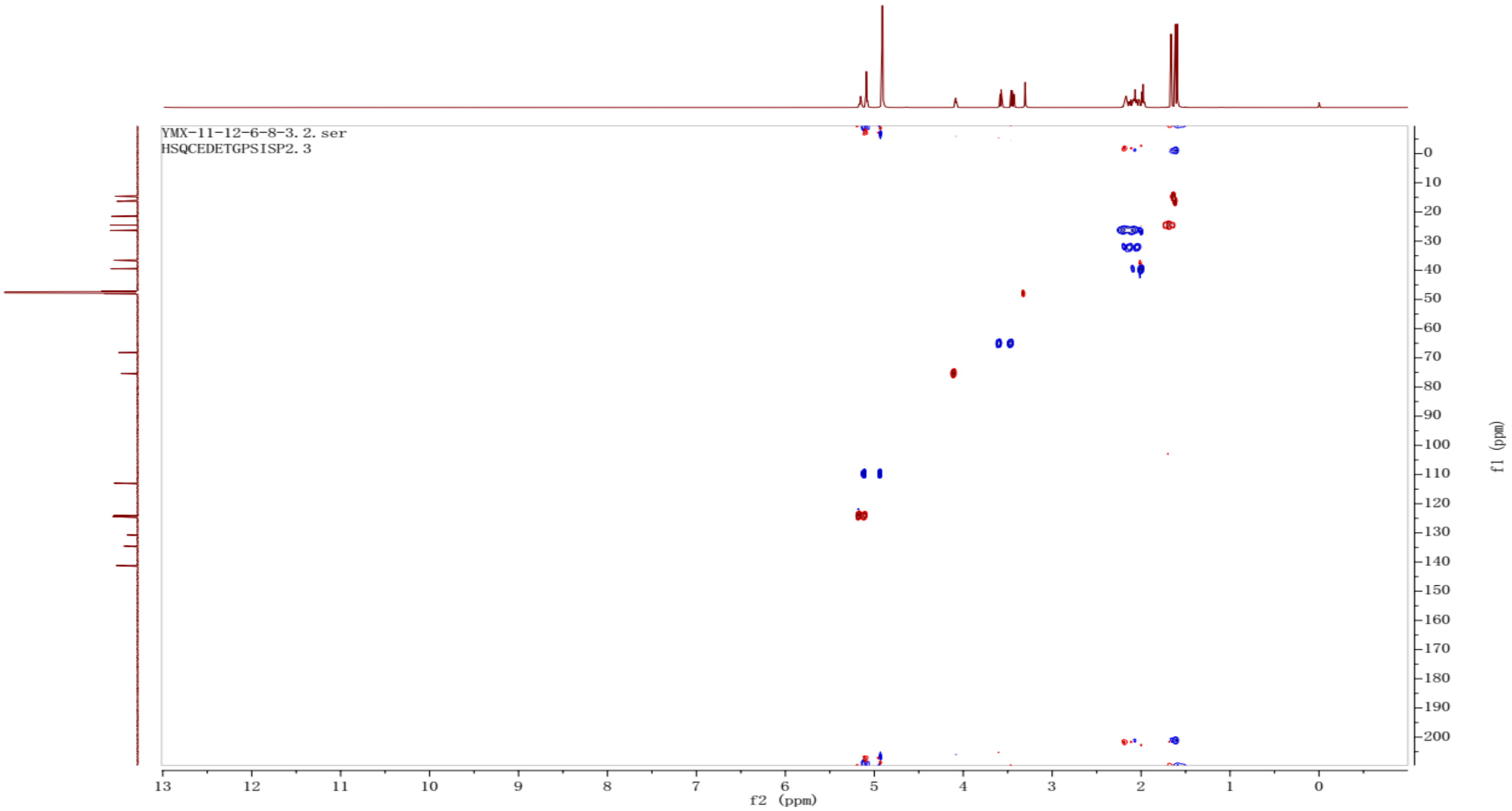

Figure S66. HSQC spectrum of compound **7** (CD_3OD, 600 MHz)

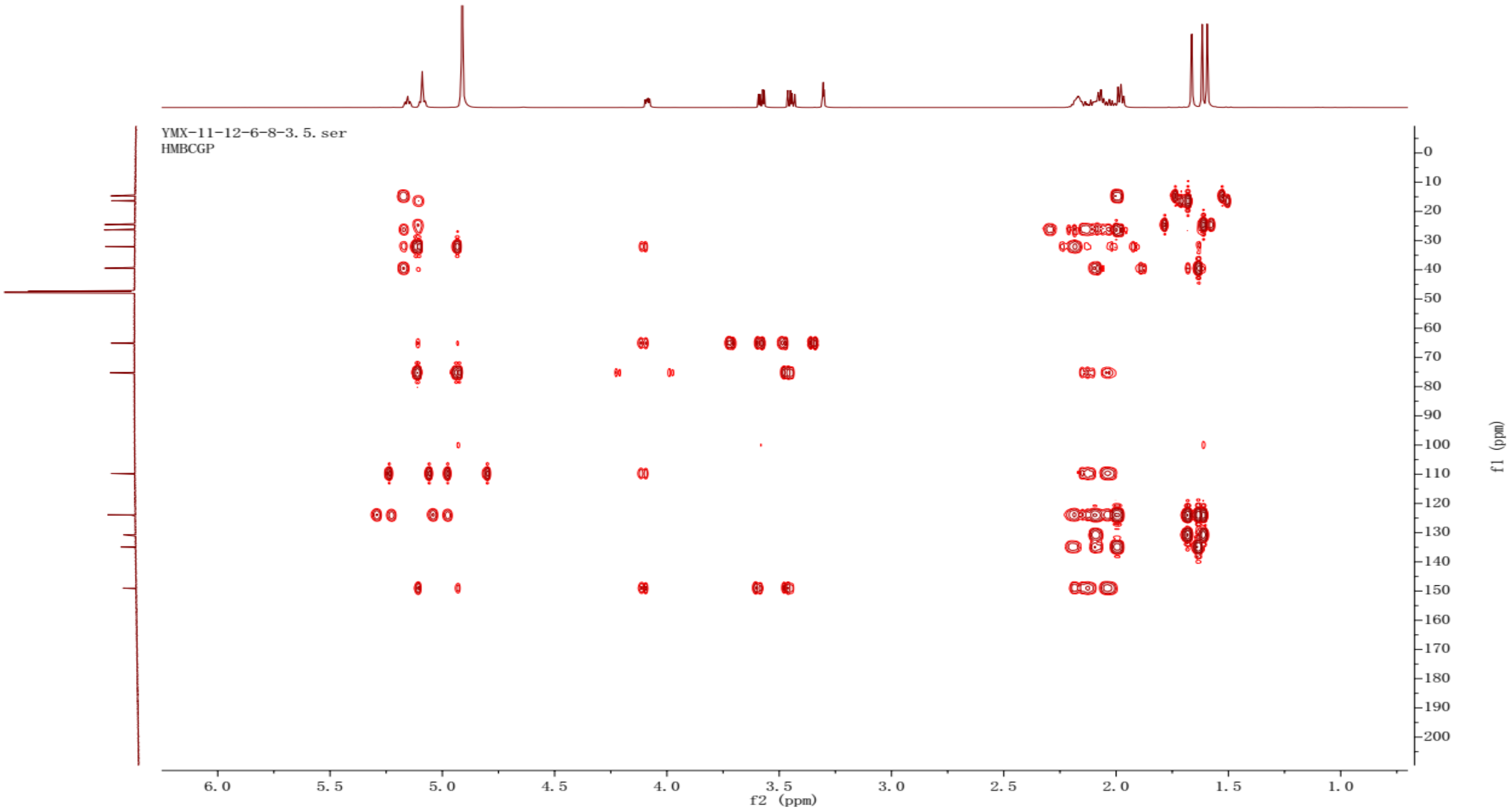

Figure S67. HMBC spectrum of compound **7** (CD_3OD, 600 MHz)

Qualitative Analysis Report

**Data Filename**	YMX-11-12-6-8-3.d	**Sample Name**	YMX-11-12-6-8-3
**Sample Type**	Sample	**Position**	p1c2
**Instrument Name**	Instrument 1	**User Name**	Q-TOF-HP\Q-TOF
**Acq Method**	default20190111-ms.m	**Acquired Time**	1/12/2020 4:11:00 PM
**IRM Calibration Status**	Success	**DA Method**	1.m
**Comment**			

Sample Group **Info.**
Acquisition SW Version 6200 series TOF/6500 series Q-TOF B.05.01 (B5125.3)

User Chromatograms

Fragmentor Voltage 155 **Collision Energy** 0 **Ionization Mode** ESI

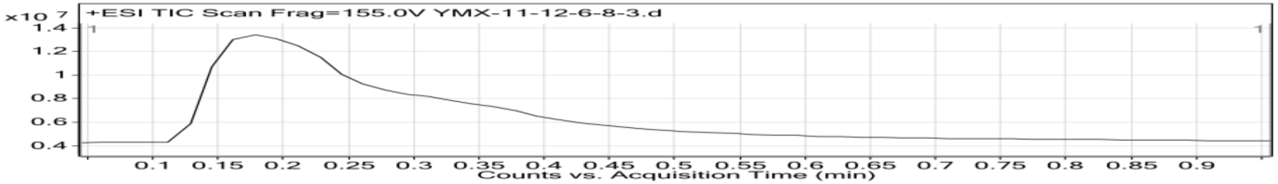

User Spectra

Fragmentor Voltage	Collision Energy	Ionization Mode
155	0	ESI

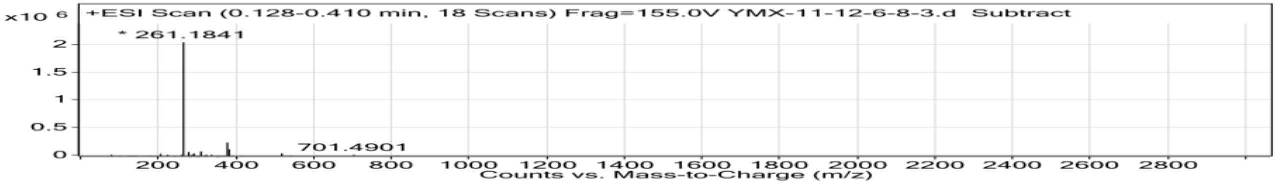

Peak List

m/z	z	Abund
261.1841	1	2048351.13
261.3211	1	247079.53
261.3652		79286.84
262.1848	1	765918.75
263.1873	1	64784.36
277.154	1	69289
287.1966	1	53615.05
305.2071	1	83044.34

Figure S68. HRESI spectrum of compound **7**

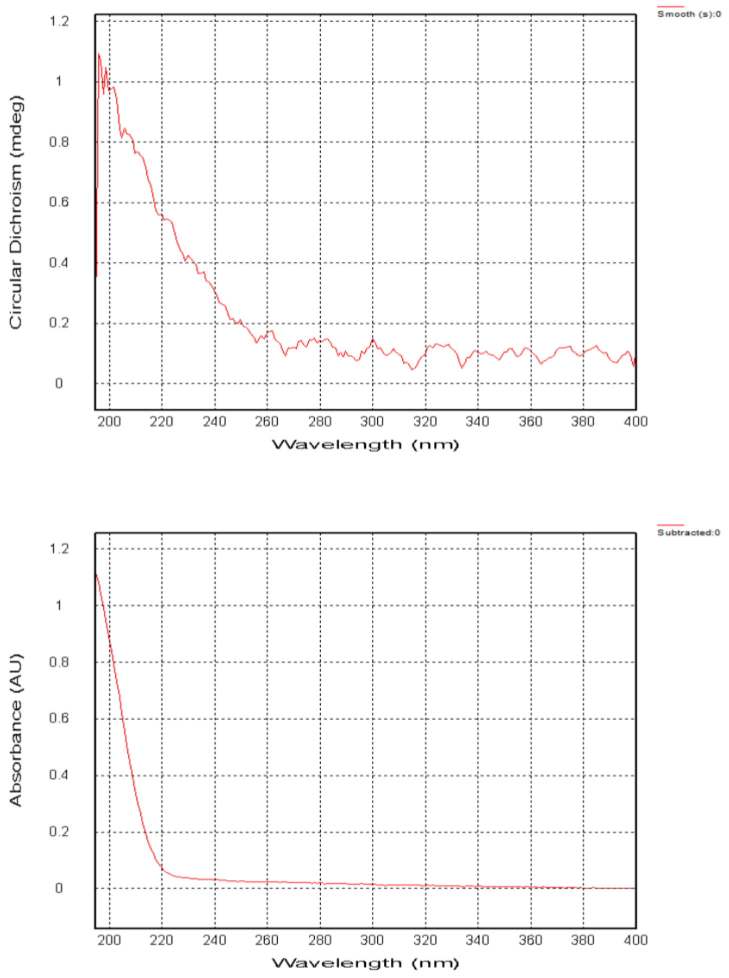

File: YMX-11-12-6-8-3 (195-400 nm) 20112721.dsx
ProBinaryX
Attributes :
- Time Stamp :Fri Nov 27 21:06:34 2020

- File ID : {B308C0DA-C265-4041-837A-C29D563B6A3C}
- Is CFR Compliant : false
- Original data has not been modified.

Remarks:
- User: CD
- Date: 2020/11/27
- Instrument: 0547
- DetectorType: LAAPD
- DichOS Calibration Correction Curve: 0547/2
- HV (CDDC channel): 0 v
- Time per point: 1 s
- Description: Sample 1
- Concentration: 0.0300mg/mL MeOH
- Pathlength: 1 mm
- Temperature: 20℃

Settings:
- Time-per-point: 1s (25us x 40000)
- SE
- Wavelength: 195nm - 400nm
- Step Size: 1nm
- Bandwidth: 1nm

Figure S69. CD and UV spectrum of compound **7**

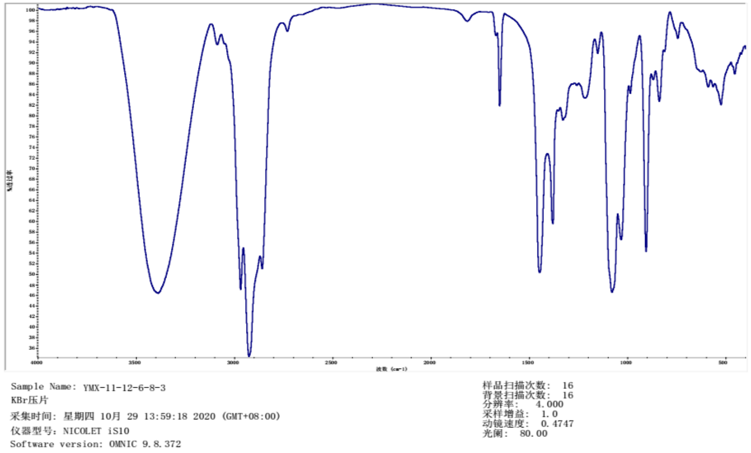

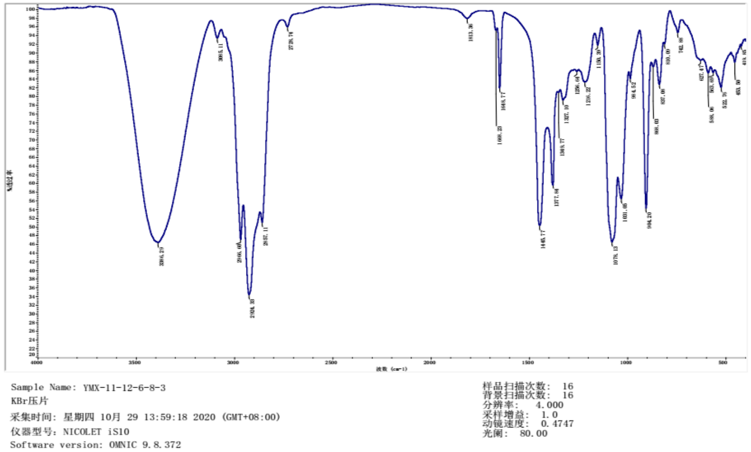

星期四 10月 29 14:08:54 2020 (GMT+08:00)
标峰:
谱图: YMX-11-12-6-8-3
范围: 4000.00 400.00
(绝对)阈值: 99.048
灵敏度: 99
峰表:

峰位置:	419.85	强度:	91.589
峰位置:	453.50	强度:	87.820
峰位置:	522.76	强度:	82.009
峰位置:	563.69	强度:	85.413
峰位置:	588.06	强度:	85.353
峰位置:	627.47	强度:	88.267
峰位置:	742.88	强度:	94.593
峰位置:	810.09	强度:	92.055
峰位置:	837.08	强度:	82.668
峰位置:	868.03	强度:	86.752
峰位置:	904.20	强度:	54.157
峰位置:	984.52	强度:	84.050
峰位置:	1031.05	强度:	56.415
峰位置:	1078.13	强度:	46.430
峰位置:	1150.39	强度:	91.709
峰位置:	1216.22	强度:	83.269
峰位置:	1256.64	强度:	85.690
峰位置:	1327.10	强度:	79.070
峰位置:	1349.77	强度:	80.820
峰位置:	1377.84	强度:	59.485
峰位置:	1445.77	强度:	50.201
峰位置:	1648.77	强度:	81.777
峰位置:	1668.23	强度:	95.109
峰位置:	1813.36	强度:	97.836
峰位置:	2728.74	强度:	95.955
峰位置:	2857.11	强度:	50.853
峰位置:	2924.33	强度:	34.298
峰位置:	2966.60	强度:	46.976
峰位置:	3085.11	强度:	93.374
峰位置:	3386.29	强度:	46.275

Figure S70. IR spectrum of compound **7**

Rudolph Research Analytical

This sample was measured on an Autopol VI, Serial #91058
Manufactured by Rudolph Research Analytical, Hackettstown, NJ, USA.

Measurement Date : Thursday, 26-NOV-2020

Set Temperature : OFF

Time Delay : Disabled

Delay between Measurement : Disabled

n	Average	Std.Dev.	% RSD	Maximum	Minimum
5	3.18	0.09	2.83	3.28	3.04

S.No	Sample ID	Time	Result	Scale	OR °Arc	WLG.nm	Lg.mm	Conc.g/100ml	Temp.
1	YMX-11-6-8-3	03:24:21 PM	3.20	SR	0.0080	589	100.00	0.250	23.4
2	YMX-11-6-8-3	03:24:30 PM	3.16	SR	0.0079	589	100.00	0.250	23.4
3	YMX-11-6-8-3	03:24:38 PM	3.28	SR	0.0082	589	100.00	0.250	23.4
4	YMX-11-6-8-3	03:24:46 PM	3.20	SR	0.0080	589	100.00	0.250	23.4
5	YMX-11-6-8-3	03:24:54 PM	3.04	SR	0.0076	589	100.00	0.250	23.4

Figure S71. $[\alpha]^{25}_{D}$ of compound **7**

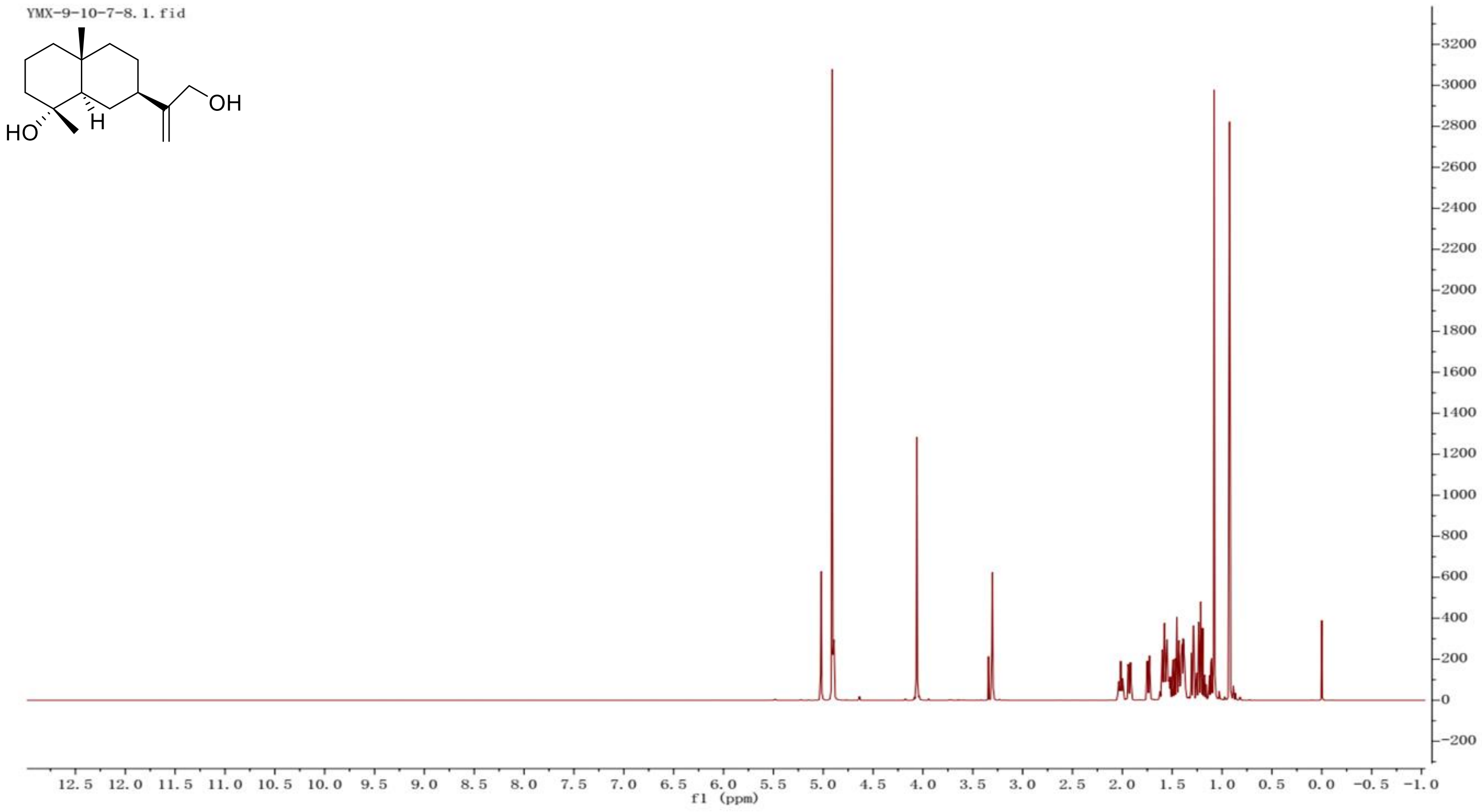

Figure S72. ^{1}H NMR spectrum of compound **10** (CD_3OD, 600 MHz)

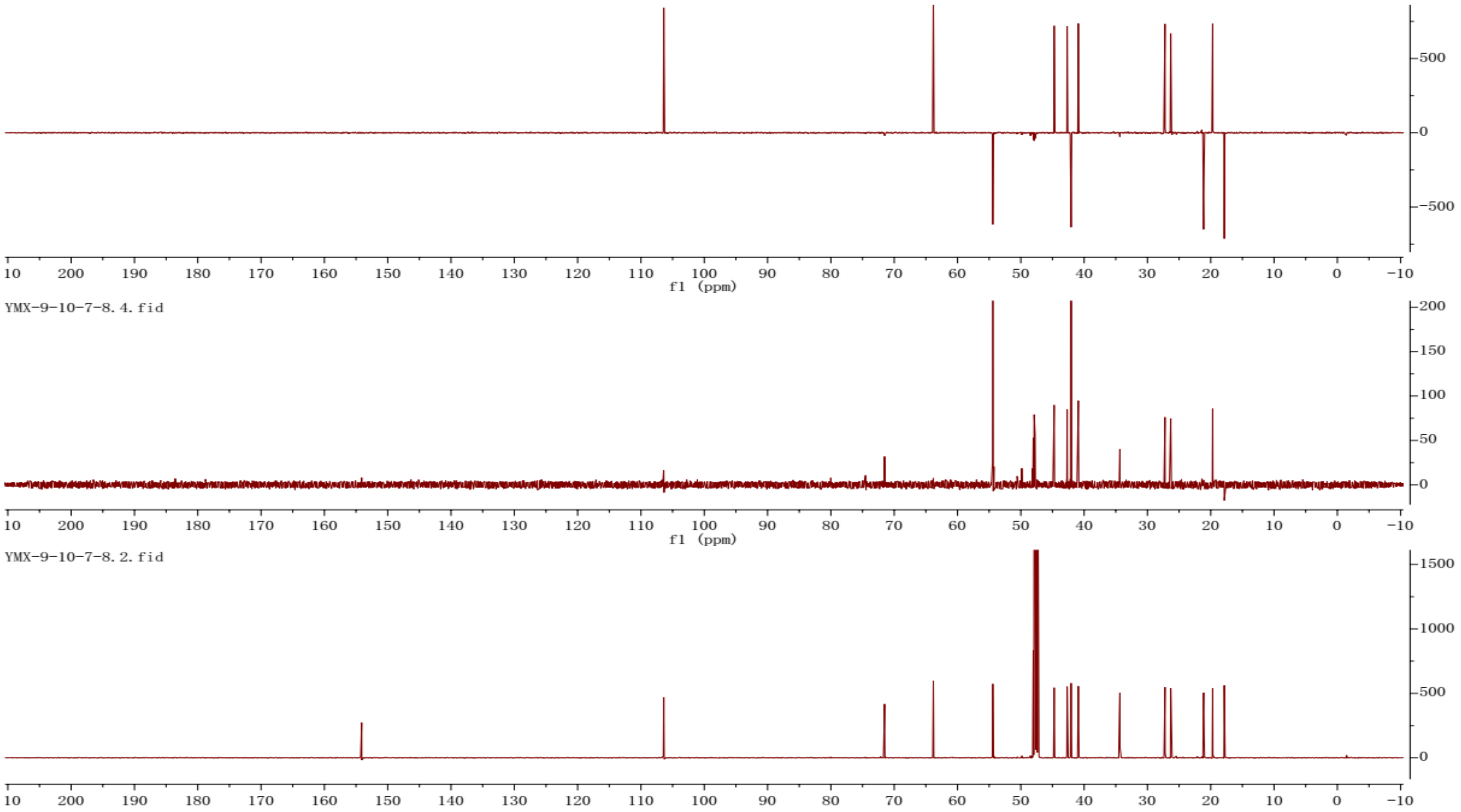

Figure S73. ^{13}C NMR and DEPT spectrum of compound **6** (CD_3OD, 150 MHz)

**Sample Name**	YMX-9-10-7-8	**Position**	P1-B9	**Instrument Name**	Instrument 1	**User Name**	
**Inj Vol**	1	**InjPosition**		**SampleType**	Sample	**IRM Calibration Status**	Success
**Data Filename**	YMX-9-10-7-8-.d	**ACQ Method**	s-.m	**Comment**		**Acquired Time**	10/25/2019 2:35:31 PM

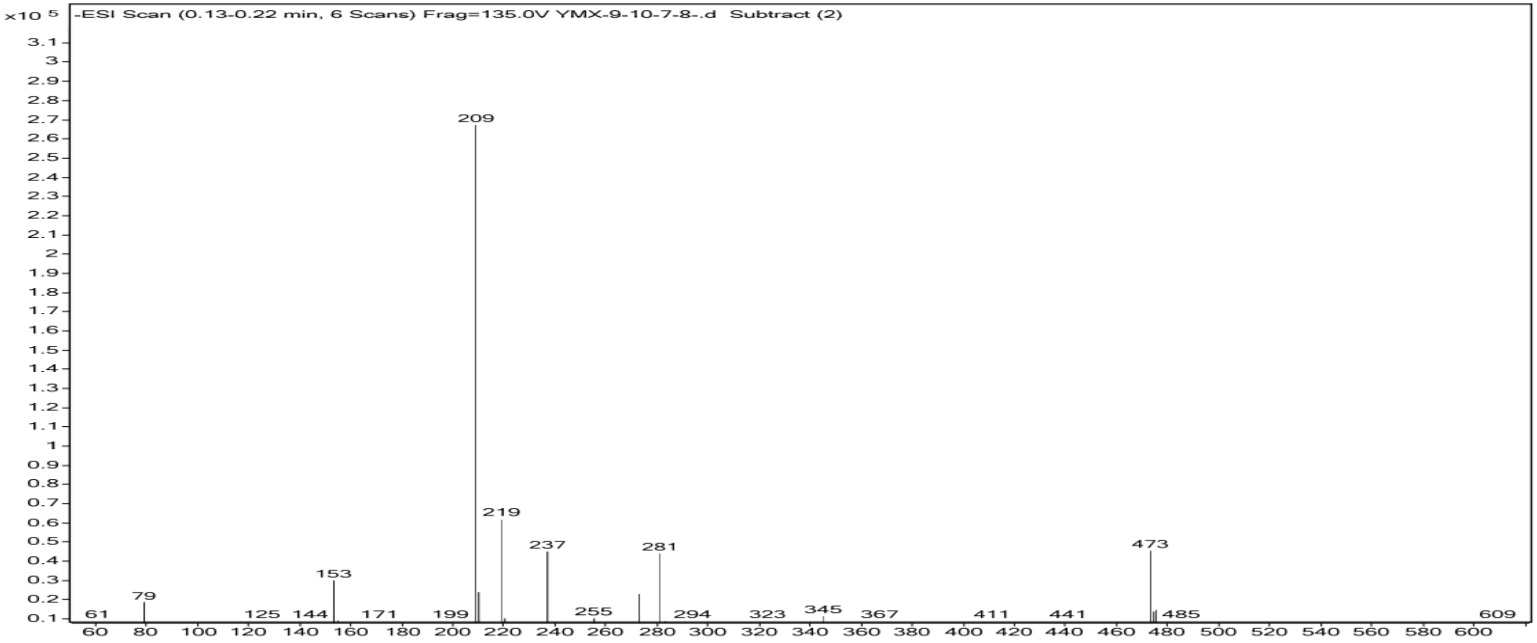

Figure S74. ESI+ spectrum of compound **10**

Crystal data for ymx-9-10-7-8: $C_{15}H_{26}O_2$, M = 238.36, a = 6.36380(10) Å, b = 9.3648(2) Å, c = 23.8658(4) Å, α = 90°, β = 90°, γ = 90°, V = 1422.30(4) Å^3, T = 100.(2) K, space group P212121, Z = 4, μ(Cu Kα) = 0.555 mm^{-1}, 11628 reflections measured, 2807 independent reflections (R_{int} = 0.0377). The final R_1 values were 0.0292 ($I > 2\sigma(I)$). The final $wR(F^2)$ values were 0.0730 ($I > 2\sigma(I)$). The final R_1 values were 0.0304 (all data). The final $wR(F^2)$ values were 0.0741 (all data). The goodness of fit on F^2 was 1.059. Flack parameter = -0.01(7).

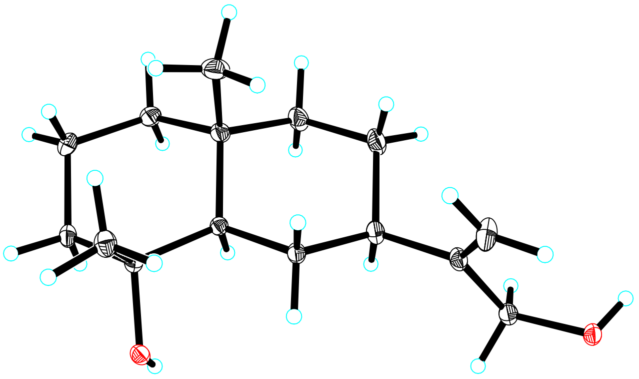

View of a molecule of ymx-9-10-7-8 with the atom-labelling scheme.

Displacement ellipsoids are drawn at the 30% probability level.

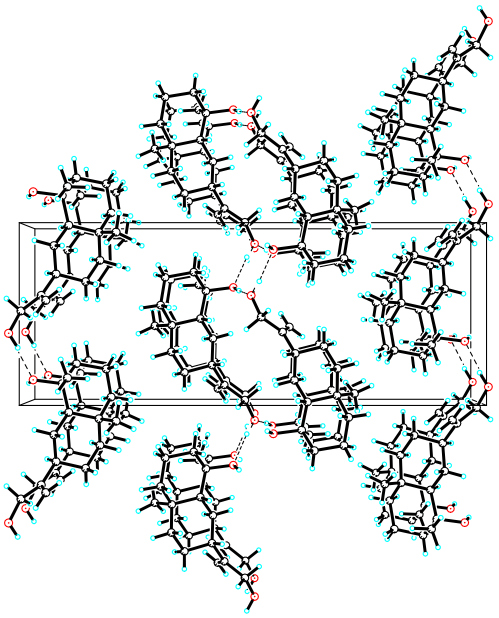

View of the pack drawing of ymx-9-10-7-8.

Hydrogen-bonds are shown as dashed lines.

Table 1. Crystal data and structure refinement for ymx-9-10-7-8.

Identification code	global	
Empirical formula	C15 H26 O2	
Formula weight	238.36	
Temperature	100(2) K	
Wavelength	1.54178 Å	
Crystal system	Orthorhombic	
Space group	$P2_12_12_1$	
Unit cell dimensions	a = 6.36380(10) Å	= 90°.
	b = 9.3648(2) Å	= 90°.
	c = 23.8658(4) Å	= 90°.
Volume	1422.30(4) Å^3	
Z	4	
Density (calculated)	1.113 Mg/m^3	
Absorption coefficient	0.555 mm^{-1}	
F(000)	528	
Crystal size	0.230 x 0.180 x 0.140 mm^3	
Theta range for data collection	3.70 to 72.17°.	
Index ranges	-6<=h<=7, -11<=k<=11, -29<=l<=29	
Reflections collected	11628	

Independent reflections	2807 [R(int) = 0.0377]
Completeness to theta = 72.17°	99.8 %
Absorption correction	Semi-empirical from equivalents
Max. and min. transmission	0.93 and 0.77
Refinement method	Full-matrix least-squares on F^2
Data / restraints / parameters	2807 / 0 / 160
Goodness-of-fit on F^2	1.059
Final R indices [I>2sigma(I)]	R1 = 0.0292, wR2 = 0.0730
R indices (all data)	R1 = 0.0304, wR2 = 0.0741
Absolute structure parameter	-0.01(7)
Largest diff. peak and hole	0.188 and -0.168 e.$Å^{-3}$

Figure S75. X-ray crystallographic analysis for compound **10**
